# Phytochemical Investigation of *Carex praecox* Schreb. and ACE-Inhibitory Activity of Oligomer Stilbenes of the Plant

**DOI:** 10.3390/molecules29143427

**Published:** 2024-07-22

**Authors:** Csilla Zsuzsanna Dávid, Norbert Kúsz, Orinamhe Godwin Agbadua, Róbert Berkecz, Annamária Kincses, Gabriella Spengler, Attila Hunyadi, Judit Hohmann, Andrea Vasas

**Affiliations:** 1 Department of Pharmacognosy, University of Szeged, 6720 Szeged, Hungary; davidzsuzsanna88@gmail.com (C.Z.D.); kusznorbert@gmail.com (N.K.); orinamhe.agbadua@uniben.edu (O.G.A.); kincses.annamaria@szte.hu (A.K.); hunyadi.attila@szte.hu (A.H.); hohmann.judit@szte.hu (J.H.); 2 Institute of Pharmaceutical Analysis, University of Szeged, 6720 Szeged, Hungary; berkecz.robert@szte.hu; 3 HUN-REN-USZ Biologically Active Natural Products Research Group, University of Szeged, Eötvös u. 6, 6720 Szeged, Hungary; 4 Department of Medical Microbiology, Albert Szent-Györgyi Health Center, Albert Szent-Györgyi Medical School, University of Szeged, 6725 Szeged, Hungary; spengler.gabriella@med.u-szeged.hu

**Keywords:** Cyperaceae, *Carex praecox*, lignans, stilbenes, flavonoids, ACE-inhibitory activity

## Abstract

Phenolic compounds are the main special metabolites of Cyperaceae species from phytochemical, pharmacological, and chemotaxonomical points of view. The present study focused on the isolation, structure determination, and pharmacological investigation of constituents from *Carex praecox*. Twenty-six compounds, including lignans, stilbenes, flavonoids, megastigmanes, chromenes, and phenylpropanoids, were identified from the methanol extract of the plant. Five of these compounds, namely, carexines A–E, are previously undescribed natural products. All compounds were isolated for the first time from *C. praecox*. The ACE-inhibitory activity of seven stilbenoid compounds was tested, and (–)-hopeaphenol proved to be the most active (IC_50_ 7.7 ± 0.9 μM). The enzyme–kinetic studies revealed a mixed-type inhibition; therefore, domain-specific studies were also conducted. The in silico docking of (–)-hopeaphenol to the ACE affirmed some favorable interactions. In addition, the antiproliferative and antibacterial effects of some compounds were also evaluated.

## 1. Introduction

Cardiovascular diseases (ischaemic heart disease and stroke) are still the leading causes of death responsible for approximately 27% of the total deaths worldwide [1,2,3]. The angiotensin I-converting enzyme (ACE), a zinc-dependent dipeptidyl carboxypeptidase, is one of the main targets in treating hypertension, heart failure, myocardial infarction, and other related diseases. The ACE is composed of two independent catalytic domains: The C-domain is mainly responsible for the conversion of angiotensin I to angiotensin II and, thus, regulates blood pressure and hydrolyzing bradykinine, while the N-domain hydrolyzes other peptides, including the hemoregulatory peptide, AcSDKP [4,5]. There are several available inhibitors of the ACE; however, most of them cause unpleasant adverse effects (e.g., dry cough, angioedema). Selective domain inhibitors might have potency in the treatment of hypertension without the undesirable adverse effects and in utilizing the different physiological effects of each ACE domain in clinical use [6].

The *Carex* genus, belonging to the family Cyperaceae (sedges), comprises approximately 2000 species that dominate wetlands, pastures, prairies, tundra, and the herb layer of temperate forests [7]. Sedges are rich sources of phenolic secondary metabolites, like stilbenes, flavonoids, and lignans, but other types of plant metabolites, such as coumarins, quinones, alkaloids, and terpenoids have also been isolated from species of this plant family [8,9,10,11]. However, up to now only a limited number of species (approx. 20) have been investigated from phytochemical and pharmacological points of view [12]. Previous studies revealed that *Carex* species are an abundant source of stilbene-type metabolites, among them monomers, dimers, trimers, and tetramers [12,13,14,15]. Cyperaceae stilbenes are mainly oligomers of piceatannol and resveratrol. A 1,2-diaryl-dihydro benzofuran skeleton with *trans*-oriented aryl rings is the most important framework in stilbene oligomers of this family, and it is considered to be biosynthesized by regio- and stereoselective pathways [16]. Stilbenes possess noteworthy biological activities and have been isolated from other heterogeneous and phylogenetically unrelated plant families, e.g., Dipterocarpaceae, Gnetaceae, Leguminosae, Polygonaceae, and Vitaceae, etc. [12,17]. Our study aimed to isolate and identify specialized metabolites, especially stilbenes, from *Carex praecox*. *C. praecox* Schreb. (early sedge, spring sedge) is a perennial, 8–30 cm plant native to Europe and western Asia and is commonly found in moist to wet habitats, forests, or mountain grasslands. There is no available information on the ethnomedicinal importance of *C. praecox* and, according to the literature, it has not been investigated either from phytochemical or pharmacological points of view.

We describe here the isolation and structure determination of carexins A–E (**1**–**5**) and the identification of 21 other compounds (**6**–**26**), among them lignans, flavonoids, stilbenes, and megastigmanes, as well as the ACE-inhibitory activity of the isolated stilbenes.

## 2. Results

### 2.1. Isolation and Structure Determination of the Compounds

**Figure 1:**
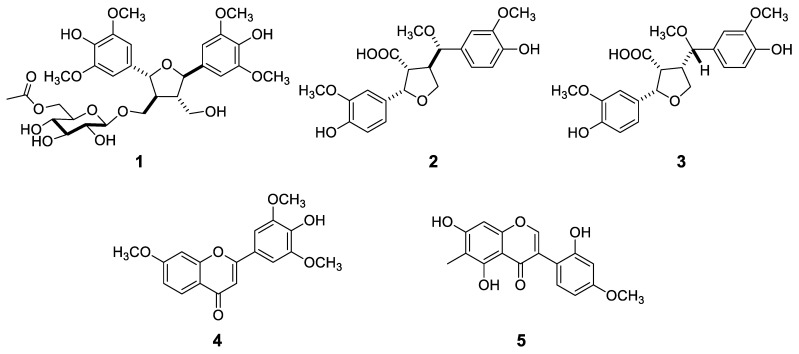
Structures of carexines A–E (**1**–**5**).

Dried and ground *C. praecox* plant material was extracted with methanol at room temperature. After evaporation, the extract was dissolved in 50% aqueous methanol and subjected to solvent–solvent partitioning with *n*-hexane, chloroform, and ethyl acetate. Both the chloroform and the ethyl acetate phases of the plant were further purified by different chromatographic techniques, including column chromatography (CC), vacuum liquid chromatography (VLC), flash chromatography (FC), rotational planar chromatography (RCP), preparative thin-layer chromatography (TLC), and HPLC to afford altogether 26 compounds, among them 5 previously undescribed natural products (carexines A–E, **1**–**5**) (Figure 1).

The structure elucidation of the isolated compounds was carried out by extensive spectroscopic analysis, applying 1D (^1^H and JMOD) and 2D (^1^H–^1^H COSY, HSQC, HMBC, and NOESY) NMR and HRMS measurements, and a comparison of the spectral data with the values in the literature.

Compound **1** was isolated as yellowish oil and exhibited a brownish-purple color on the TLC plate by spraying with vanillin sulfuric acid and then heating. The molecular formula was determined as C_30_H_40_O_15_ from molecular ion peaks [M + NH_4_]^+^ at *m/z* 658.2711 (calcd for C_30_H_44_NO_15_^+^ 658.2711) and [M + Na]^+^ *m/z* 663.2258 (calcd for C_30_H_40_O_15_Na^+^ 663.2264) in the HRESIMS. The ^1^H NMR spectrum displayed signals due to four aromatic protons [*δ*_H_ 6.74 (2H, s, H-2, H-6), 6.71 (2H, s, H-2′, H-6′)]; two methines [*δ*_H_ 2.44 (1H, m, H-8), 2.32 (1H, m, H-8′)]; two oxymethylenes [*δ*_H_ 3.96 (1H, dd, *J* = 10.1, 5.0 Hz, H-9a), 3.65 (1H, dd, *J* = 10.1, 5.0 Hz, H-9b), 3.71 (1H, dd, *J* = 11.6, 4.4 Hz, H-9′a), 3.59 (1H, dd, *J* = 11.6, 4.4 Hz, H-9′b)]; two oxymethines [*δ*_H_ 5.05 (1H, d, *J* = 8.4 Hz, H-7), 4.98 (1H, d, *J* = 8.5 Hz, H-7′)]; four methoxy groups [*δ*_H_ 3.83 (12H, s, CH_3_O-3, CH_3_O-5, CH_3_O-3′, CH_3_O-5′)]; one methyl group [*δ*_H_ 1.94 (3H), s]; and an anomeric proton [*δ*_H_ 4.25 (1H, d, *J* = 7.9 Hz, H-1″)] (Table 1). The ^13^C NMR spectrum showed signals due to a glucopyranosyl group [*δ*_C_ 64.6 (C-6″), 71.6 (C-4″), 75.1 (C-2″), 78.0 (C-3″), 75.3 (C-5″), 104.7 (C-1″)]; one methyl group (*δ*_C_ 20.6); and a carbonyl group (*δ*_C_ 172.7). The relatively large ^3^*J*_1″–2″_ value (7.8 Hz) of the anomeric proton of this glucopyranosyl group indicated a *β*-orientation.

**Figure 2:**
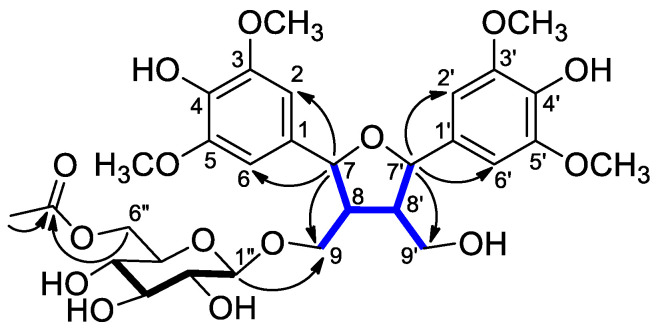
Diagnostic ^1^H–^1^H COSY (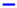) and HMBCs (H → C) of compound **1**.

From these data, the aglycone suggested a 2,5-diaryl-tetrahydrofuranoid-type lignan, and based on the comparison of its NMR data with the literature values, it was identified as icariol A_2_ [18]. HMBC spectrum determined the position of the *β*-d-glucopyranosyl group to be at C-9 by showing correlations between the anomeric proton (*δ*_H_ 4.25) and C-9 (*δ*_C_ 69.9) of the aglycone, as shown in Figure 2. This derivative is also known in nature [19]. Moreover, the HMBC between the methyl protons (*δ*_H_ 1.94) and methylene protons of H_2_-6″ (*δ*_H_ 4.33 and 4.15) with carbonyl carbon (*δ*_C_ 172.7) determined that the glucose moiety was acetylated at C-6″.

The NOESY correlations confirmed the position of the *β*-d-glucopyranosyl group to be at C-9 by showing correlations between H-1″ and H-9a and H-9b. The NOESY correlations were detected between H-7 and H-9a/9b and H-7′ and H-9′a/9′b, proving H-7 to be on the same side as 9-methylene, and H-7′ on the same side as 9′-methylene. This is in agreement with the coupling constants of H-7 (*J*_7,8_ = 8.4 Hz) and H-7′ (*J*_7′,8′_ = 8.5 Hz) in *trans* position with H-8 and H-8′, respectively. Thus, the structure of **1** was assigned as icariol A_2_ 9-*O*-*β*-d-(6″-acetyl)-glucopyranoside, a new natural compound, and named carexine A (Figure 1).

**Figure 3:**
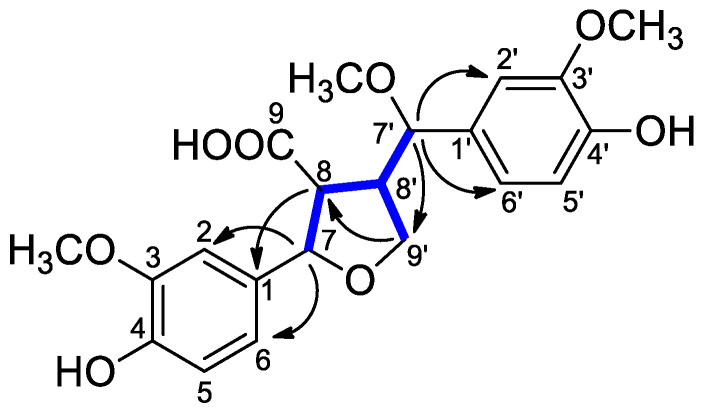
Diagnostic ^1^H-^1^H COSY (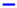) and HMBCs (H → C) of compound **2**.

The molecular formula of carexine B (**2**) was determined as C_21_H_24_O_8_ from a molecular ion peak at *m/z* 403.1443 [M − H]^−^ (calcd for C_21_H_23_O_8_^−^ 403.1441) in the HRESIMS. The ^1^H NMR spectrum of compound **2** displayed six olefin methine proton signals at *δ*_H_ 6.91 (1H, d, *J* = 1.8 Hz, H-2); 6.78 (1H, d, *J* = 8.1 Hz, H-5); 6.77 (1H, dd, *J* = 8.1, 1.8 Hz, H-6); 6.92 (1H, d, *J* = 1.8 Hz, H-2′); 6.80 (1H, d, *J* = 8.0 Hz, H-5′); and 6.78 (1H, dd, *J* = 8.0, 1.8 Hz, H-6′) due to two 1,3,4-trisubstituted benzene rings (Table 2). The proton signals at *δ*_H_ 5.07 (1H, d, *J* = 7.2 Hz, H-7) and 4.26 (1H, d, *J* = 10.4 Hz, H-7′) were interpreted as two oxygenated methines, and the proton signals at *δ*_H_ 3.63 (1H, t, *J* = 8.6, 7.6 Hz, H-9′a) and 3.56 (1H, t, *J* = 8.6 Hz, H-9′b) were due to an oxygenated methylene showing geminal (^2^*J*) coupling. Moreover, the proton signals at *δ*_H_ 3.84, 3.87, and 3.06 (3 × 3H, s) indicated the presence of three methoxy groups in the molecule. The remaining proton signals at *δ*_H_ 3.07 (1H, m, H-8) and 3.00 (1H, m, H-8′) were determined as two methines, suggesting the presence of a furanolignan skeleton. In the JMOD spectrum, 18 carbon signals, including a carboxyl group [*δ*_C_ 176.6 (C-9)] were observed, confirming **2** to be a lignan substituted with three methoxy (*δ*_C_ 2 × 56.4 and 56.0) and two hydroxyl groups. The partial structures of compound **2** were determined by a ^1^H–^1^H COSY experiment (Figure 3). Correlations were observed between *δ*_H_ 3.00 (H-8′), with the oxygenated methylene proton signals at *δ*_H_ 3.63 (H-9′a) and 3.56 (H-9′b), and the methine proton signals at *δ*_H_ 3.07 (H-8) and *δ*_H_ 4.26 (H-7′). Moreover, *δ*_H_ 3.07 (H-8) correlated with *δ*_H_ 5.07 (H-7). The location of the phenyl, methoxy, and carboxyl groups of compound **2** was determined by the HMBC experiment (Figure 3). HMBCs were observed between the methine proton signal H-7′, with the aromatic methine carbon signals at *δ*_C_ 121.5 (C-6′) and 111.4 (C-2′), methoxy carbon signal at *δ*_C_ 56.0 (O*C*H_3_-7′) and methine signal at *δ*_C_ 51.2 (C-8′). The methine proton signal H-8 showed a correlation with the carboxy carbonyl signal at *δ*_C_ 176.6 (C-9) and the quaternary carbon signal at *δ*_C_ 133.9 (C-1). Finally, diagnostic HMBCs were observed between H-7 and aromatic ring signals at *δ*_C_ 110.6 (C-2) and 119.7 (C-6). Therefore, the structure of compound **2** was determined to be 8-carboxyl-4,4′-dihydroxy-3,3′,7′ trimethoxy-7,9′-epoxy-8,8′-lignan, and named carexine B.

**Figure 4:**
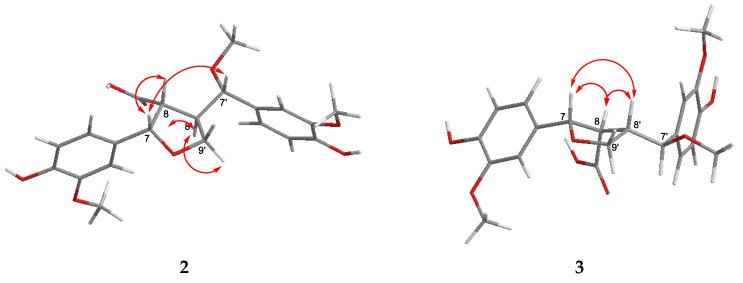
Key NOESY correlations (⟷) of compounds **2** and **3**.

In the NOESY spectrum, the cross peaks between H-7/H-8, H-7/H-9′b, H-7/H-7′, and H-7′/H-9′b indicated the *β*-configuration of these protons, while the NOE correlation between H-9′a and H-8′ indicated the *α*-configuration of H-9′a and H-8′ (Figure 4).

The molecular formula and molecular weight of carexine C (**3**) were found to be the same as those of compound 2, based on the HRESIMS data. Only slight differences could be observed in the 1D and 2D NMR spectra (Table 2). The only difference between the two compounds was the *cis* orientation of H-8/H-8′, as shown by the NOESY correlation between H-8′ and H-8 and H-8′ and H-7 (Figure 4). Previously, a lignan (vibruresinol) with the same skeleton was reported from the stems of *Viburnum erosum* [20].

**Figure 5:**
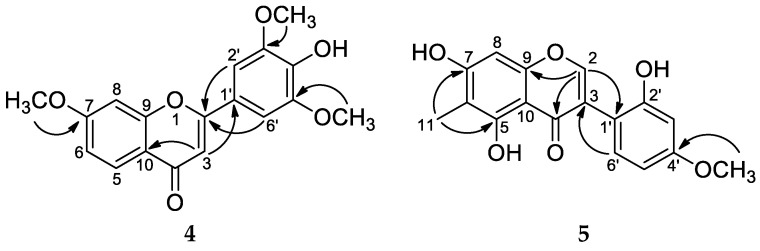
Diagnostic HMBCs (H → C) of compounds **4** and **5**.

Carexine D (**4**) was obtained as a pale yellow amorphous powder, and its molecular formula was established as C_18_H_16_O_6_ from the HRESIMS peak at *m/z* 329.1018 [M + H]^+^ (calcd for C_18_H_17_O_6_, 329.1020). The ^1^H NMR spectrum of **4** exhibited six aromatic proton signals, among them an ABX system at *δ*_H_ 8.04 (1H, d, *J* = 8.9 Hz, H-5); 7.07 (1H, dd, *J* = 8.9, 2.4 Hz, H-6); 7.28 (1H, d, *J* = 2.4 Hz, H-8)]; two singlet protons at *δ*_H_ 7.33 (2H, s, H-2′, H-6′); and one methine proton [*δ*_H_ 6.78 (1H, s, H-3)]. Besides the aromatic protons, signals of three methoxy groups at *δ*_H_ 3.98 (3H, OCH_3_) and 3.97 (6H, 2 × OCH_3_) could be detected. In the JMOD spectrum, signals assigned to a 15-carbon-containing flavonoid skeleton were detected (Table 3). The aromatic singlet at *δ*_H_ 6.78 was assigned to H-3 as it showed an HMBC with C-10 (*δ*_C_ 118.2), C-2 (*δ*_C_ 166.1), and C-1′ (*δ*_C_ 123.0) (Figure 5). The three methoxy groups were assigned to be attached to C-7, C-3′, and C-5′, which were determined by HMBC cross-peaks from *δ*_H_ 3.98 to *δ*_C_ 166.4 (C-7), and *δ*_H_ 3.97 to *δ*_C_ 149.8 (C-3′ and C-5′). The connection of ring B to ring C was proved by the HMBC correlation detected between H-2′,6′, and C-2. Interestingly, ring A is substituted only with a methoxy group at C-7. Hence, compound **4** was determined as 4′-hydroxy-7,3′,5′-trimethoxyflavone, a new flavonoid named carexine D.

Carexine E (**5**) has the molecular formula C_17_H_14_O_6_ compatible with the protonated molecular peak at *m/z* 315.0865 [M + H]^+^ (calcd for C_17_H_15_O_6_^+^ 315.0869) in the HRESIMS. In the ^1^H NMR spectrum, five aromatic proton signals at *δ*_H_ 8.00 (1H, s, H-2); 7.13 (1H, d, *J* = 8.3 Hz, H-6′); 6.49 (1H, dd, *J* = 8.3, 2.5 Hz, H-5′); 6.48 (1H, br s, H-3′) and 6.41 (1H, s, H-8); one methoxy signal at *δ*_H_ 3.78 (3H, s, CH_3_O-4′); and one methyl signal (*δ*_H_ 2.07, s, CH_3_-11) were detected. The isoflavone nature of **5** was evident from the H-2 chemical shift of the singlet at *δ*_H_ 8.00 and from HMBCs detected between H-2 and C-9, C-4, and C-1′ and between H-6′ and C-3. Based on the ^1^H NMR and JMOD spectra, compound **5** is a penta-substituted isoflavone. The position of the substituents was determined by HMBC measurement, long-range correlations were observed between C*H*_3_-11 and C-5, C-6, and C-7 and between OC*H*_3_-4′ and C-4′, proving that the methyl group is joined at C-6, and the methoxy group is attached to C-4′. Therefore, compound **5** was determined as 5,7,2′-trihydroxy-4′-methoxy-6-methylisoflavone and named carexine E.

**Figure 6:**
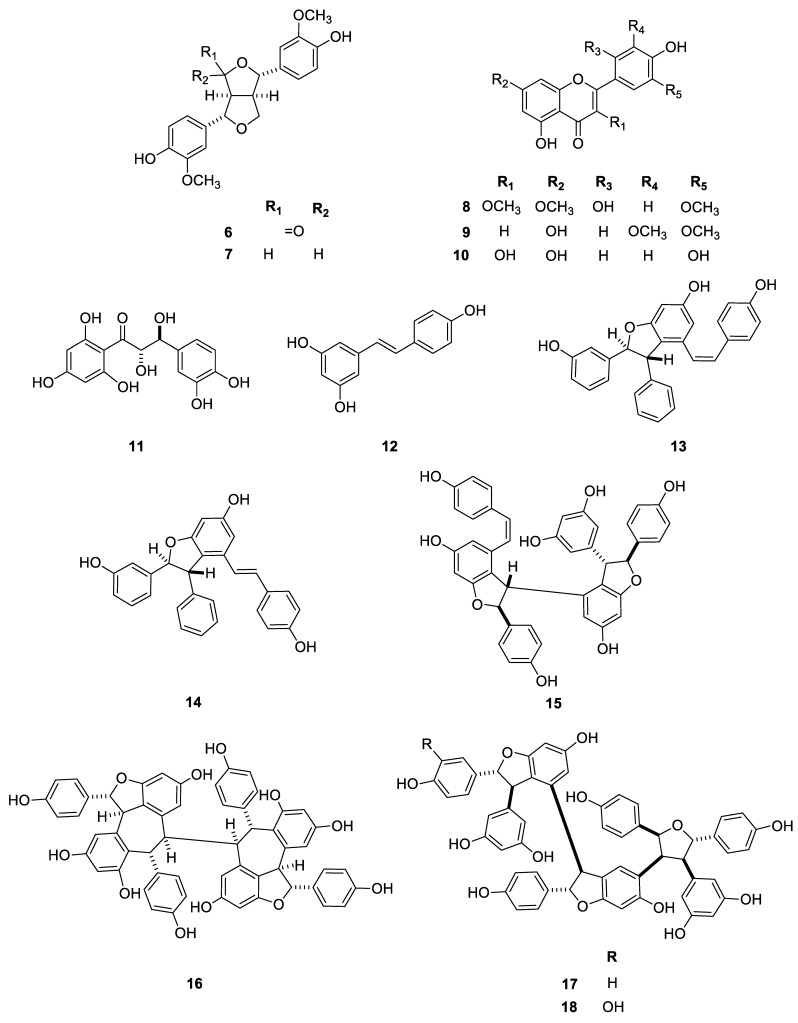

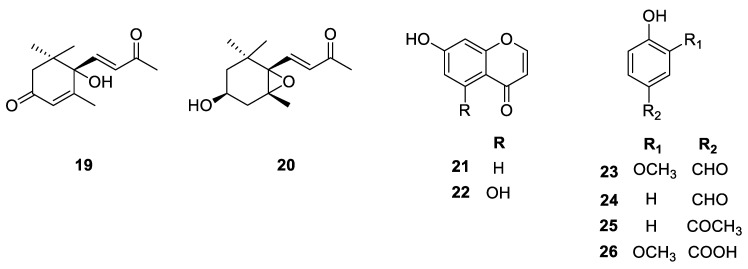
Structures of other compounds (**6**–**26**) isolated from *C. praecox*.

Besides the new compounds carexines A–E (**1**–**5**), 21 known compounds were also isolated from *C. praecox*. The structural characterization was performed through HRESIMS, 1D, and 2D NMR experiments and then by comparison of the ^1^H and ^13^C assignations with reported literature data. All compounds were isolated for the first time from this plant. The compounds were identified as two known lignans [4-ketopinoresinol **6**) [21] and (+)-pinoresinol (**7**)] [22], three flavonoids [chrysosplenol F (**8**) [23], tricin (**9**) [24], quercetin (**10**) [25], the chalcone cilicicone b (**11**) [26], seven stilbenes [*trans*-resveratrol (**12**) [27], *cis*-*ε*-viniferin (**13**) [28], *trans*-*ε*-viniferin (**14**) [28], *Z*-miyabenol C (**15**) [29], (–)-hopeaphenol (**16**) [30], kobophenol A (**17**) [29], carexinol A (**18**) [15], two megastigmanes, namely, (*S*)-(+)-dehydrovomifoliol (**19**) [31] and 5α,6α-epoxy-3β-hydroxy-7-megastimen-9-one (**20**) [32], two chromones [7-hydroxychromone (**21**) [33] and 5,7-dihydroxychromone (**22**) [34], and other phenolic compounds [vanillin (**23**) [35], *p*-hydroxybenzaldehyde (**24**), piceol (**25**) [36], and vanillic acid (**26**) [35] (Figure 6). Previously unpublished ^1^H and ^13^C NMR data of compound **8** (chrysosplenol F) and ^1^H NMR data of **9** in methanol are given in the experimental section.

Based on the isolated compounds, the main constituents of *C. praecox* are stilbenoids. Among the stilbenoids, monomers [*trans*-resveratrol (**12**)], dimers [*cis*- and *trans*-*ε*-viniferin (**13**, **14**)], trimers [*Z*-miyabenol C (**15**)], tetramers [(–)-hopeaphenol (**16**), kobophenol A (**17**), and carexinol A (**18**)] were identified. Compounds **12**, **13**, **15**, **17,** and **18** were isolated previously from other Cyperaceae species [12]. The lignans identified from *C. praecox* are tetrahydrofuranoid-type ones. The chromones are known as flavonoid degradation products.

According to our phytochemical results, *C. praecox* is a rich source of polyphenolic compounds. Polyphenols, including flavonoids, stilbenes, phenolic acids, lignans, and others, possess different health benefits [37]. They are secondary plant metabolites implicated in protection against pathogens and ultraviolet radiation and have allelopathic effects [38]. Due to their known antioxidant activity, they have been attributed a probable role in preventing various diseases associated with oxidative stress, such as cancer and cardiovascular and neurodegenerative diseases [39]. It is hypothesized that the increasing concentration of complex stilbenes often occurs in response to plant stresses (via unknown mechanisms) and potentially enhances antioxidant activity and antifungal capacities [40].

Megastigmanes are identified as phytotoxic compounds. Several compounds inhibited the germination of *Lactuca sativa* seeds [41], e.g., 5,7-dihydroxy chromone (**22**) inhibited the germination of velvetleaf seeds; therefore, it has allelopathic activity [42]. *p*-Cresol (**25**) also possessed an allelopathic effect [43]. Tricin (**9**) has been previously isolated from other Cyperaceae species, such as *Cyperus exaltatus* var. *iwasakii* [44], *Rhynchospora corymbose* [45], and *Cyperus rotundus* [46]. Tricin (**9**) exerts unique biological activities over other flavonoids, such as antileishmanial [47], and antihistaminic [48] effects, and has a protective effect against UV-B-irradiation-caused skin damage [49].

Hopeaphenol (**16**) was the first oligostilbene to be isolated in 1951 from *Hopea odorata* (Dipterocarpaceae) [50]. (–)-Hopeaphenol (**16**), was identified as a selective inhibitor of HIV transcription that targets, in part, PKC- and NF-κB-mediated HIV transcription and CDK9 activity in T cells, resulting in the inhibition of virus production in vitro and infectious virus replication in peripheral blood mononuclear cells (PBMCs) [51]. The compound also inhibited cellular entry of SARS-CoV-2 USA-WA1/2020, B.1.1.7, and B.1.351 variants [52].

### 2.2. Pharmacological Assays

The isolated stilbenes (**12**–**18**) were subjected to different pharmacological studies. To investigate the potential cardioprotective effect of the isolated stilbenes, an ACE-inhibitory assay was performed, and the IC_50_ values of the compounds were determined. All the tested stilbenes (except resveratrol (**12**)) exerted notable activity at a concentration of 90 μM; among them, the tetramer (–)-hopeaphenol (**16**) was the most active, with an IC_50_ value of 7.7 ± 0.9 μM (Table 4).

For a better understanding of the actual interaction between (–)-hopeaphenol (**16**) and the ACE, a domain-specific assay was performed. The inhibitory activity of compound **16** compared to bradykinin-potentiating peptide b (BPPb) on the C- and N-domain of a rabbit lung ACE using the FRET substrates was investigated. Based on the results, (–)-hopeaphenol (**16**) inhibits the N-domain favorably (IC_50_ = 35.67 ± 2.3 μM), while it has a 10 times lower affinity for the C-domain (IC_50_ > 300 μM) (Table 5).

Since selective inhibition of the N-domain will result in the accumulation of AcSDKP, it might be promising for treating fibrosis without affecting blood pressure [53]. Furthermore, inhibition of the N-terminal ACE-active site may have important clinical applications in facilitating hematopoietic recovery after aggressive cancer chemotherapy by controlling the hematopoietic cycle and stem cell proliferation [54].

### 2.3. Molecular Docking

**Figure 7:**
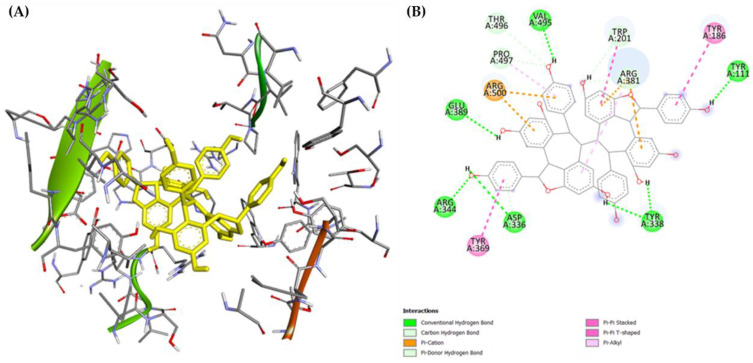
Binding of (–)-hopeaphenol (**16**) to the N-domain. (**A**): 3-D interactions of (–)-hopeaphenol (**16**) in yellow, with the active site amino acids. (**B**): 2-D interaction showing the type of bonding interactions with the active site residues.

In silico docking was applied to characterize the binding behavior of (–)-hopeaphenol (**16**). The energy-minimized model of compound (**16**) was docked using AutoDock4 into both domains of the ACE crystal structure retrieved from the Protein Data Bank (PDB ID: 1O86 for the C-domain and 2C6N for the N-domain) to explain the chemical interactions between (–)-hopeaphenol (**16**) and both ACE active binding sites. This program uses the Lamarckian genetic algorithm (LGA) to generate a range of potential conformations from a starting ligand in an arbitrary conformation and then searches for favorable dockings at the protein-binding site [55]. The docking results revealed a network of hydrogen bonds and electrostatic and hydrophobic interactions between (–)-hopeaphenol (**16**) and the N-domain of the ACE with a strong binding energy (E = −9.83 kcal/mol). Importantly, interaction with residues Tyr^369^, Arg^381^, and Thr^496^ could be detected; these residues were previously connected to the N-domain selectivity [56]. The most important interactions are the π–π interactions between (–)-hopeaphenol (**16**) and Tyr^369^ and the carbon–hydrogen bond with Arg^381^ (Figure 7).

**Figure 8:**
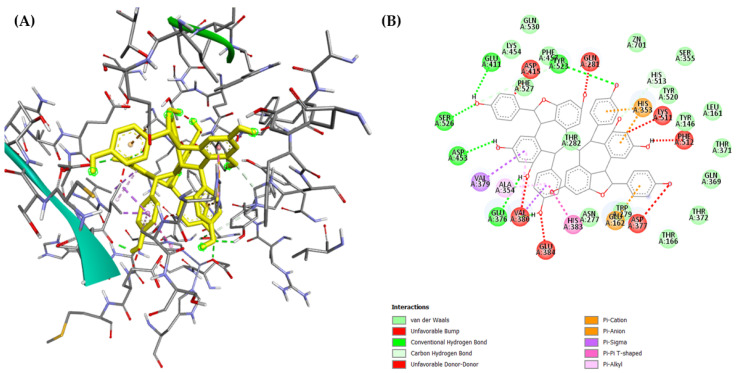
Binding of (–)-hopeaphenol (**16**) to the C-domain. (**A**): 3-D interactions of (–)-hopeaphenol (**16**) in yellow, with the active site amino acid residues. (**B**): 2-D interaction showing the type of bonding interactions, with several unfavorable interactions observed.

On the other hand, (–)-hopeaphenol (**16**) bound to the C-domain with a very poor affinity (binding energy: E = +41.42 kcal/mol). Several unfavorable interactions were identified as the reason for the low binding efficiency (Figure 8). This is well in line with the results of the in vitro domain-specific studies, suggesting that (–)-hopeaphenol (**16**) is an N-domain-selective ACE inhibitor.

### 2.4. Other Pharmacological Assays

The antiproliferative capacity of compounds **12**–**18** was tested against human colon adenocarcinoma cells (Colo 205 sensitive and the resistant Colo 320/MDR-LRP expressing ABCB1). A thiazolyl blue tetrazolium bromide (MTT) assay was used for each compound to assess the concentration required for 50% inhibition of viability of the cell population (IC_50_), and cisplatin and doxorubicin were used as positive controls. Interestingly, only the monomer resveratrol (**12**) and the tetramer (–)-hopeaphenol (**16**) exerted notable antiproliferative activity against both cell lines with IC_50_ values comparable to those of the two positive controls, while the other stilbenes were found inactive (Table 6).

The antibacterial effect of the *n*-hexane, chloroform, and ethyl acetate fractions of the methanol extract of *C. praecox* was investigated against *Bacillus subtilis*, *Escherichia coli*, *Klebsiella pneumoniae*, *Moraxella catarrhalis*, MRSA, *Pseudomonas aeruginosa*, *Staphylococcus aureus*, *S. epidermidis*, and *Streptococcus pyogenes* using the disk diffusion method. The ethyl acetate fraction showed remarkable activity against *S. epidermidis* (inhibitory zone 18 mm), *S. aureus* (14 mm), MRSA (14 mm), *M. catarrhalis* (9 mm), and *B. subtilis* (12 mm), respectively. Therefore, the antibacterial effect of the isolated stilbenes (**12**–**18**) was also investigated against these bacterial strains, but none of the compounds possessed remarkable antibacterial activity (data not indicated).

## 3. Materials and Methods

### 3.1. General Experimental Procedures

NMR spectra were recorded in methanol-*d*_4_, chloroform (CDCl_3_), and DMSO-*d*_6_ on a Bruker Avance DRX 500 spectrometer (Bruker, Ettlingen, Germany) at 500 MHz (^1^H) and 125 MHz (^13^C). The signals of the deuterated solvents were chosen as references. The chemical shift values (*δ*) were given in ppm, and the coupling constants (*J*) are in Hz. The two-dimensional (2D) experiments were conducted using standard TopSpin 3.6.1 Bruker software. Gradient-enhanced versions were applied in correlation spectroscopy (^1^H–^1^H COSY), nuclear Overhauser effect spectroscopy (NOESY), heteronuclear single quantum coherence spectroscopy (HSQC), and heteronuclear multiple bond correlation (HMBC) experiments. The high-resolution MS spectra were acquired with a Thermo Scientific Q-Exactive Plus Orbitrap mass spectrometer (Thermo Fisher Scientific, Waltham, MA, USA) equipped with an ESI ion source in positive ionization mode. The data were acquired and processed with MassLynx software 4.1 (SCN805). Optical rotation measurements were carried out by a Jasco-P2000 digital polarimeter (JASCO Corporation, Tokyo, Japan).

The normal and reversed-phase vacuum liquid chromatography (VLC) were carried out on a silica gel (Kieselgel 60 GF_254_, 15 μm, Merck, Darmstadt, Germany) and on a reversed-phase silica gel [RediSep C-18, 40–60 μm, Teledyne Isco, Lincoln, NE, USA]. The thin-layer chromatography was performed on Kieselgel 60 RP-18 F_254_ and Kieselgel 60 F_254_ (Merck, Darmstadt, Germany). The TLC plates were detected under a UV light at 254 nm and by spraying with a vanillin–sulfuric acid reagent, followed by heating. The flash chromatography (FC) was processed with a Combi Flash Rf^+^ Lumen instrument (Teledyne Isco) on a reversed-phase RediSep Rf HP Gold (50 g) column. Sephadex LH-20 (25–100 μm, Sigma-Aldrich, St. Louis, MO, USA) was used for gel filtration. The rotation planar chromatography (RPC) was carried out using a Chromatotron instrument (Model 8924, Harrison Research, T-Squared Technology, Inc., San Bruno, CA, USA). The high-performance liquid chromatographic (HPLC) separation was carried out on a Waters HPLC (Waters 600 controller, Waters 600 pump, and Waters 2998 photodiode array detector, Waters, Milford, MA, USA) and a Shimadzu HPLC (Shimadzu LC40, Shimadzu, Tokyo, Japan) using normal phase [LiChrospher Si 100 (250 × 4 mm, 5 μm, Merck)] and reversed-phase [Kinetex C18 (150 × 4.6 mm, 5 μm, 100 Å, Phenomenex, Torrance, CA, USA) and Kinetex Phenyl-hexyl] columns. The flow rate was 1 mL/min, and the injection volume was 10 μL. The data were acquired and processed with Empower 3 and LabSolutions software 5.111. All solvents used for CC were of at least analytical grade (VWR Ltd., Szeged, Hungary). Ultrapure water was prepared with a Milli-Q water purification system (Millipore, Molsheim, France).

### 3.2. Plant Material

The whole plants of *Carex praecox* Schreb. (1.8 kg of dried plant material) were collected during the flowering period in Besenyőtelek, Hungary (GPS coordinates: 47.691670, 20.438270) in July 2019 and were identified by László Bakacsy (Department of Plant Biology, University of Szeged, 6726 Szeged, Hungary). A voucher specimen (No. 899) was deposited in the Herbarium of the Department of Pharmacognosy, University of Szeged, Szeged, Hungary.

### 3.3. Extraction and Isolation

The dried and ground whole *C. praecox* plant (1.8 kg) was percolated with methanol (MeOH, 50 L) at room temperature. The methanolic extract was concentrated in vacuo (310 g), dissolved in 50% aqueous methanol (1500 mL), and subjected to solvent–solvent partition with *n*-hexane (10 × 500 mL), chloroform (CHCl_3_, 10 × 500 mL), and ethyl acetate (EtOAc, 10 × 500 mL).

The concentrated CHCl_3_-soluble fraction (11 g) was further separated by the normal phase (NP) vacuum liquid chromatography (VLC) applying a gradient solvent system of CHCl_3_–MeOH [from 100:0 to 8:2, and finally MeOH (500 mL/eluent)] resulting in 11 main fractions (C/A-K). Fraction C/B (354 mg) was further separated by a reversed-phase (RP) MPLC, affording 10 subfractions (C/B/1–10). From fraction C/B/1, compound **23** (1.4 mg) and from fraction C/B/7, compound **8** (3.8 mg) were isolated using an NP and RP preparative thin-layer chromatography (prep TLC), and by NP-HPLC. Fraction C/C (482 mg) was further purified by an RP flash chromatography (FC) to yield 12 subfractions (C/C/1–12). Fraction C/C/1 was chromatographed using RP-VLC, RP-prep TLC, and RP-HPLC methods to yield compounds **6** (3 mg), **7** (2.7 mg), **2** (1.6 mg), and **3** (1.5 mg), while subfraction C/C/4 was purified by an NP-prep TLC and afforded compounds **5** (18.7 mg) and **4** (2.8 mg). Fraction C/D (203 mg) was subjected to gel chromatography using Sephadex LH-20 gel as a stationary phase and a mixture of MeOH–CH_2_CL_2_ (1:1) as an eluent, which resulted in seven subfractions (C/D/1–7). Fraction C/D/4 was purified by RP-prep TLC and RP-HPLC, and compounds **19** (1.4 mg) and **20** (4.5 mg) were isolated, while fraction C/D/6 afforded compound **22** (4.6 mg). Fraction C/E (564 mg) was subjected to an RP-VLC, applying gradient elution with MeOH–H_2_O [from 1:9 to 9:1, and finally MeOH (300 mL/eluent)], resulting in 8 subfractions (C/E/1–8). From fraction C/E/6, compounds **24** (4.1 mg) and **25** (2.4 mg) were isolated through RP- and NP-VLC and an NP-HPLC. Fraction C/F (710 mg) was separated by an RP-MPLC, providing 14 subfractions (C/F/1–14). Using the RP-HPLC (gradient elution with acetonitrile–H_2_O), the purification of fraction C/F/2 afforded compound **21** (2.6 mg), while C/F/13 yielded compound **9** (4.0 mg).

The concentrated EtOAc-soluble fraction (10 g) was subjected to VLC on NP silica gel, with a gradient system of CHCl_3_–MeOH [from 98:2 to 7:3, and finally MeOH (800 mL/eluent)], to yield 11 main fractions (E/A-K). Fractions E/B (77 mg), E/D (339 mg), E/F (538 mg), E/G (2020 mg), and E/H (526 mg) were separated by gel chromatography on the Sephadex LH-20 stationary phase using MeOH–CH_2_Cl_2_ (1:1) as an eluent, yielding 4 (E/B/1–4), 8 (E/D/1–8), 9 (E/F/1–9), 8 (E/G/1–8), and 8 (E/H/1–8) subfractions, respectively. From fraction E/B/3, compound **26** (1.2 mg) was isolated via RP-HPLC. Fraction E/D/6 was purified via RP-prep TLC and RP-HPLC, and compounds **11** (12.9 mg) and **12** (1.2 mg) were obtained, while fraction E/D/8 proved to be pure for compound **10** (25.0 mg). From fraction E/F/2, compound **1** (8.8 mg) was obtained by RP-prep TLC (MeOH–H_2_O 6:4). Fraction E/F/7 was subjected to rotational planar chromatography (RPC) [gradient system of CHCl_3_–MeOH from 99:1 to 7:3, and finally MeOH (100 mL/eluent)] affording 5 subfractions. After purification by RP-HPLC (MeOH–H_2_O gradient system), fraction E/F/7/3 yielded compounds **13** (10.7 mg) and **14** (27.1 mg). From fraction E/F/7/4, compound **15** (5.9 mg) was isolated by RP-HPLC (MeOH–H_2_O gradient system). Fraction E/H/6 was further purified by RP-prep TLC, and RP-HPLC to obtain compounds **17** (16.4 mg) and **18** (3.6 mg). Fraction E/I (1631 mg) was subjected to RP-VLC, applying MeOH–H_2_O gradient elution [from 1:9 to 8:2, and finally MeOH (300 mL/eluent)] to afford 5 subfractions. After purification by RP-prep TLC (MeOH–H_2_O 1:1), fraction E/I/3 yielded compound **16** (4.7 mg).

*Carexine A* (**1**): yellowish oil; [α${]}_{D}^{26}$ −6.0 (*c* 0.20, MeOH); ^1^H and ^13^C NMR data (CD_3_OD, see Table 1); HRESIMS *m/z* 658.2711 [M + NH_4_]^+^ (calcd for C_30_H_44_NO_15_ 658.2711), 663.2258 [M + Na]^+^ (calcd for C_30_H_40_O_15_Na 663.2264).

*Carexine B* (**2**): white amorphous solid; [α${]}_{D}^{26}$−14.4 (*c* 0.05, MeOH); ^1^H and ^13^C NMR data (CD_3_OD, see Table 2); HRESIMS *m/z* 403.1443 [M − H]^−^ (calcd for C_21_H_23_ O_8_ 403.1441).

*Carexine C* (**3**): pale yellow amorphous solid; [α${]}_{D}^{26}$ +7.6 (*c* 0.05, MeOH); ^1^H and ^13^C NMR data (CD_3_OD, see Table 2); HRESIMS *m/z* 403.1443 [M −H]^−^ (calcd for C_21_H_23_ O_8_ 403.1441).

*Carexine D* (**4**): pale yellow amorphous solid; ^1^H and ^13^C NMR data (CD_3_OD, see Table 3); HRESIMS *m/z* 329.1018 [M + H]^+^ (calcd for C_18_H_17_O_6_ 329.1020).

*Carexine E* (**5**): light brown amorphous solid; ^1^H and ^13^C NMR data (CD_3_OD, see Table 3); HRESIMS *m/z* 315.0865 [M + H]^+^ (calcd for C_17_H_14_O_6_ 315.0869).

Compound **8** (*chrysosplenol F*): ^1^H NMR (CD_3_OD) *δ*_H_ 7.02 (1H, s, H-6′), 6.55 (1H, d, *J* = 2.2 Hz, H-8), 6.48 (1H, s, H-3′), 6.34 (1H, d, *J* = 2.2 Hz, H-6), 3.87 (3H, s, 7-OMe), 3.83 (3H, s, 5′-OMe), 3.74 (3H, s, 3-OMe); *δ*_C_ 180.0 (C-4), 167.3 (C-7), 162.9 (C-5), 159.2* (C-2), 159.0* (C-9), 152.3^#^ (C-2′), 152.1^#^ (C-4′), 142.8 (C-5′), 140.4 (C-3), 114.6 (C-6′), 109.1 (C-1′), 107.1 (C-10), 93.3 (C-8), 105.3 (C-3′), 99.0 (C-6), 56.5 (OMe-7), 57.3 (OMe-5′), 61.2 (OMe-3); *^,#^ are interchangeable.

Compound **9** (*tricin*): ^1^H NMR (CD_3_OD) *δ*_H_ 7.25 (2H, s, H-2′,6′), 6.63 (1H, s, H-3), 6.45 (1H, d, *J* = 2.0 Hz, H-8), 6.2 (1H, d, *J* = 2.0 Hz, H-6), 3.95 (6H, s, 2 × OMe, 3′,5′).

### 3.4. Pharmacological Assays

#### 3.4.1. ACE-Inhibitory Assay

Angiotensin-converting enzyme inhibition was determined using the Angiotensin-I Converting Enzyme (ACE) Activity Assay Kit (CS0002, Sigma-Aldrich, USA) with modification of the volume of enzyme and samples added to the wells. In summary, a 96-well black plate (655096, F-bottom, Grenier bio-one, Frickenhausen, Germany) containing 25 μL of samples diluted in methanol-assay buffer was filled with 25 μL of enzyme solution (each 25 μL contained 1.5 mU of the ACE: ACE from rabbit lung, A6778, Sigma-Aldrich, USA). The solution was incubated for 5 min at 37 °C with shaking, and then 50 μL of the substrate (a 100-fold dilution of the substrate provided) was added. A plate reader (BMG Lambtech GmbH, Ortenberg, Germany) was used to monitor the fluorescence for 5 min in kinetic mode at Ex/Em 290/450 nm as soon as the substrate was added. At the end of the measurement, the percentage inhibition by each compound was calculated as follows:

% inhibition = (values without samples − sample values)/(values without samples) × 100.(1)

Dose–effect studies on the compounds **12**–**18** were used to determine the concentration that inhibits 50% of the ACE. ACE-inhibitory kinetic studies were performed on compound **16**, the most potent compound, to determine its inhibition mechanism. Similarly, 25 μL of enzyme was added to plate wells containing 25 μL of several concentrations of compound **16** (0–10 μM). Following a 5 min incubation period at 37 °C, 50 μL of the substrate Abz-Gly-Phe (NO_2_)-Pro (4003531, Bachem, Bubendorf, Switzerland) was added at varying concentrations (125–500 μM). The plate reader was then used to monitor the fluorescence in kinetic mode at extinction values of Ex/Em 290/450 nm. The Michaelis constant (Km) and maximal velocity (Vmax) of ACE were determined via Lineweaver–Burk plots, using the pharmacological and biochemistry transform and simple linear regression functions of the software, GraphPad Prism 8.0 (La Jolla, CA, USA).

#### 3.4.2. Domain-Specific Studies

ACE domain-specific inhibition studies were performed based on previously reported methods (Carmona et al., 2006 [57]; Lunow et al., 2015 [58]). Fluorescence resonance energy transfer (FRET) substrates, Abz-SDK(Dnp)P-OH and Abz-LFK(Dnp)-OH were used for the N-domain and C-domain, respectively. The initial velocity of the reaction was determined using various concentrations (1–128 μM) of the FRET substrates. Briefly, 40 μL of assay buffer and 60 μL of the FRET substrate solutions were preincubated for 10 min at 37 °C, with the reaction started by adding 20 μL of diluted ACE solution (5 μL ACE + 15 μL 0.1 mol/L TRIS buffer) and fluorescence measured at λex/λem = 290/450 nm every minute at 37 °C for 30 min. The corresponding K_M_ of the FRET substrates, determined using the Michaelis–Menten equation, was used as the substrate concentration of the FRET substrates used in percent inhibition studies. The inhibitory activity of 16 on both domains was determined as described above with the 40 μL solution containing the inhibitor (inhibitor in DMSO–Assay buffer, 1:9). The control samples, which correspond to 100% enzyme activity, were prepared by replacing the inhibitor solution with TRIS buffer. Dose–effect studies on 16 using the FRET substrates were used to determine the IC_50_ of this compound on both ACE domains. All experiments were performed in triplicates. The ACE-inhibitory activity was calculated using the following equation:

(%) = (Ab − Aa) − (Cb − Ca)/(Ab − Aa) × 100(2)

where Aa is the absorbance of control wells at 0 min; Ab is the absorbance of control wells at 15 min; Ca is the absorbance of the inhibitor wells at 0 min; and Cb is the absorbance of inhibitor wells at 15 min.

#### 3.4.3. Molecular Docking

The structure of the compound was drawn using a ChemDraw 12.0.2 software (ACD/LABS, Advanced Chemistry Development, Inc., Toronto, ON, Canada), and the energy of the compound (**16**) was minimized at the default mode, using a minimum RMS gradient of 0.010 in the software, Chem3D Pro 12.0 (ACD/LABS, Advanced Chemistry Development, Inc.). The energy-minimized compound was subsequently saved in PDB format before using it in the docking procedure. The X-ray crystallographic structures of the C- and N-domains of the human angiotensin I-converting enzyme complexed with lisinopril were obtained from the RCSB Protein Data Bank (PDB ID: 1O86 and 2C6N, respectively) [56,59]. PDB files for the enzyme and compounds were converted to the PDBQT format using the graphical user interface, AutoDock4 (The Scripps Research Institute, La Jolla, CA, USA) [60].

Before the docking analysis, water molecules and the lisinopril were eliminated from the 1O86 ACE protein model (C-domain) using AutoDock 4.2 (The Scripps Research Institute), while the zinc and chlorine atoms were retained in the ACE protein model, as these have been reported to be essential for the activity of ACE. By adding polar hydrogens, combining non-polar hydrogens, and adding Kollman charge to 1O86 using AutoDockTools, the final receptor for docking was created. A grid box (X: 43.817, Y: 38.308, and Z: 46.652, with 50 × 70 × 50 grid points of 0.375 Å spacing) created to include all active residues around the Zn(II) prosthetic group was used to calculate the zinc-centered map for ACE 1O86 [61].

The N-domain enzyme, 2C6N, was prepared using an identical method to the one used in preparing the C-domain enzyme, except that the protein’s water molecules and sugar moieties were eliminated along with the lisinopril. To encompass all active residues and Zn(II) heteroatom in the A chain of this domain [62], a grid box (X: −28.034, Y: −24.612, and Z: −33.992; number of grid points in the three dimensions [npts]: X: 70, Y: 70, and Z: 60; spacing: 0.375 Å) was defined.

The docking procedure was performed with 10 docking runs. The Lamarckian algorithm was used to dock ligands once the docking parameters were set to their default settings. The binding energies were obtained from the resulting DLG files, and visualization of the interactions was achieved via Biovia (Discovery Studio visualizer version 21.1.0.20298; Dassault Systèmes, Vélizy-Villacoublay, France) after conversion of the docked PDBQT files into PDB files using OpenBabel GUI software version 2.4.1 [63].

#### 3.4.4. Cell Line Cultures

Two human colon adenocarcinoma cell lines, namely Colo 205 (ATCC-CCL-222) doxorubicin-sensitive parent cells and Colo 320/MDR-LRP (ATCC-CCL-220.1) doxorubicin-resistant cells expressing ABCB1, were purchased from LGC Promochem (Teddington, UK). The cells were cultured in an RPMI-1640 medium supplemented with 10% heat-inactivated fetal bovine serum (FBS), 2 mM of l-glutamine, 1 mM of sodium pyruvate, and 100 mM of HEPES. The cells were incubated at 37 °C, in a 5% CO_2_ and 95% air atmosphere.

#### 3.4.5. Antiproliferative Assay

The antiproliferative effects of the compounds were tested in decreasing serial dilutions (2-fold dilutions starting from 100 μM) of human cancer cell lines (Colo 205, Colo 320) in 96-well flat-bottomed microtiter plates. First, the compounds were diluted in 100 μL of the medium, and then 6 × 10^3^ cells in 100 μL of RPMI medium were added to each well, excluding the medium control wells. The culture plates were incubated at 37 °C for 72 h. Following incubation, 20 μL of the MTT solution (from a 5 mg/mL stock solution) was added to each well. After incubation at 37 °C for 4 h, 100 μL of sodium dodecyl sulfate (SDS) solution (10% SDS in 0.01 M HCl) was added to each well, and the plates were further incubated at 37 °C overnight. Cell growth was determined by measuring the optical density at 540 nm (ref.: 630 nm) using a Multiscan EX ELISA reader (Thermo Labsystems, Cheshire, WA, USA). The concentration that decreased cell viability by 50% was expressed as the IC_50_ (μM) ± SD for each compound, derived using the log (inhibitor) vs. response nonlinear fitting model of GraphPad Prism Version 9.4.0.

#### 3.4.6. Bacterial Strains and Culture Conditions for Antimicrobial Assays

The test microorganisms included the standard Gram-positive strains *Staphylococcus aureus* (ATCC 29213), methicillin-resistant *S. aureus* (MRSA) (ATCC 43300), *S. epidermidis* (ATCC 12228), *Streptococcus pyogenes* (ATCC 19615), and *Bacillus subtilis* (ATCC 6633). The standard Gram-negative strains were *Escherichia coli* (ATCC 35218), *Klebsiella pneumoniae* (ATCC 700603), *Pseudomonas aeruginosa* (ATCC 27853), and *Moraxella catarrhalis* (ATCC 25238). The bacterial cultures were grown on a standard Mueller–Hinton (MH) agar at 37 °C overnight in an aerobic environment.

#### 3.4.7. Determination of Antibacterial Activity Using the Disk Diffusion Method

The disk diffusion method was employed to screen compounds for their antibacterial activities against standard bacterial strains to determine their inhibitory zones. Briefly, the samples were dissolved in DMSO at a concentration of 10 mM. Sterile filter paper disks [6 mm in diameter, Whatman antibiotic paper disk (Cytiva, Marlborough, MA, USA)] coated with 10 μL of the sample solutions were placed on top of the bacterial suspension (inoculum: 0.5 McFarland, 1.5 × 10^8^ CFU·mL^−1^). The disks containing antibiotics (ciprofloxacin and ampicillin) were used as positive controls, and the disks containing DMSO served as negative controls. Under aerobic conditions, the plates were incubated at 37 °C for 20 h. The diameters of the zones of inhibition caused by the compounds, including the disk, were measured in triplicate. For each of the three repetitions, an average zone of inhibition was calculated [64].

## 4. Conclusions

The phytochemical investigation of a Hungarian sedge, *C. praecox*, resulted in the isolation and identification of 26 compounds, among which there are 5 new natural compounds, carexines A–E (**1**–**5**). Considering the chemical characteristics of the isolated compounds, the main constituents of *C. praecox* are phenolic compounds, mainly stilbenes (**12**–**18**). The ACE-inhibitory capacity of the isolated stilbenes was evaluated, the tetramer (–)-hopeaphenol (**16**) being the most potent inhibitor of the enzyme. The domain-specific assay revealed that hopeaphenol binds favorably to the N-domain of the ACE, which was also affirmed by the in silico docking studies. Selective inhibitors of the N-domain could be applied to treat tissue injury and fibrosis without affecting blood pressure and facilitate hematopoietic recovery after cancer chemotherapy.

## Figures and Tables

**Table 1:** ^1^H (500 MHz) and ^13^C (125 MHz) NMR Data of Compound **1**.

Position	^1^H	^13^C	Position	^1^H	^13^C
	*δ*_H_ (ppm), *J* (Hz)	*δ*_C_, Type		*δ*_H_ (ppm), *J* (Hz)	*δ*_C_, Type
1		134.3, C	1′		134.3, C
2,6	6.74 s	104.9, CH	2′,6′	6.71 s	104.9, CH
3		149.3, C	3′		149.3, C
4		136.1, * C	4′		136.2, * C
5		149.3, C	5′		149.3, C
7	5.05 d (8.4)	84.6, CH	7′	4.98 d (8.5)	84.3, CH
8	2.44 m	51.9, CH	8′	2.32 m	54.5, CH
9	3.96 dd (10.1, 5.0), 3.65 dd (10.1, 5.0)	69.9, CH_2_	9′	3.71 dd (11.6, 4.4), 3.59 dd (11.6, 4.4)	61.0, CH_2_
1″	4.25 d (7.9)	104.7, CH			
2″	3.16 br t (7.9)	75.1, CH			
3″	3.30 m	78.0, CH			
4″	3.25 m	71.6, CH			
5″	3.41 m	75.3, CH			
6″	4.33 dd (11.9, 2.1), 4.15 dd (11.9, 5.8)	64.6, CH_2_			
3/3′-OCH_3_	3.83 s	56.9, CH_3_			
5/5′-OCH_3_	3.83 s	56.9, CH_3_			
Ac-CO		172.7, C			
Ac-Me	1.94 s	20.6, CH_3_			

* interchangeable signals.

**Table 2:** ^1^H (500 MHz) and ^13^C (125 MHz) NMR Data of Compounds **2** and **3**.

	2		3	
Position	^1^H	^13^C	^1^H	^13^C
	*δ*_H_ (ppm), *J* (Hz)	*δ*_C_, Type	*δ*_H_ (ppm), *J* (Hz)	*δ*_C_, Type
1	-	133.9, C	-	134.9, C
2	6.91 d (1.8)	110.6, CH	6.90 d (1.6)	110.6, CH
3	-	149.1, C	-	148.9, C
4	-	147.9, * C	-	147.1, C
5	6.78 d (8.1)	116.1, CH	6.77 d (8.1)	116.2, CH
6	6.77 dd (8.1, 1.8)	119.7, CH	6.78 dd (8.1, 1.6)	119.6, CH
7	5.07 d (7.2)	86.0, CH	5.12 d (5.9)	85.5, CH
8	3.07 m	56.4, CH	2.72 dd (8.4, 5.9)	56.4, CH
9	-	176.6, C	-	176.8, C
1′	-	132.8, C	-	132.9, C
2′	6.92 d (1.8)	111.4, CH	6.85 d (1.6)	111.7, CH
3′	-	149.4, C	-	149.1, C
4′	-	147.4, * C	-	147.3, C
5′	6.80 d (8.0)	116.1, CH	6.73 d (8.1)	116.0, CH
6′	6.78 dd (8.0, 1.8)	121.5, CH	6.76 dd (8.1, 1.6)	121.0, CH
7′	4.26 d (10.4)	83.6, CH	4.51 d (7.1)	83.3, CH
8′	3.00 m	51.2, CH	2.90 m	50.3, CH
9′a (α)	3.63 t (8.6, 7.6)	71.4, CH_2_	4.15 d (7.9) 2H	71.6, CH_2_
9′b (β)	3.56 t (8.6)			
3-OCH_3_	3.84 s	56.4, CH_3_	3.80 s	56.4, CH_3_
3′-OCH_3_	3.87 s	56.4, CH_3_	3.84 s	56.4, CH_3_
7′-OCH_3_	3.06 s	56.0, CH_3_	3.15 s	56.6, CH_3_

* interchangeable signals.

**Table 3:** ^1^H (500 MHz) and ^13^C (125 MHz) NMR Data of Compounds **4** and **5**.

	4		5	
Position	^1^H	^13^C	^1^H	^13^C
	*δ*_H_ (ppm), *J* (Hz)	*δ*_C_, Type	*δ*_H_ (ppm), *J* (Hz)	*δ*_C_, Type
2		166.1, C	8.00 s	156.5, C
3	6.78 s	106.1, CH		122.3, C
4		180.2, C		182.6, C
5	8.04 d (8.9)	127.5, C		160.5, C
6	7.07 dd (8.9, 2.4)	116.1, CH		109.1, C
7		166.4, C		164.0, C
8	7.28 d (2.4)	101.7, CH	6.41 s	93.8, CH
9		159.7, C		157.5, C
10		118.2		105.9, C
11	-	-	2.07 s	7.4, CH_3_
1′		123.0, C		112.3, C
2′	7.33 s	105.5, CH		157.8, C
3′		149.8, C	6.48 br s	103.1, CH
4′		141.2, CH		162.7, C
5′		149.8, C	6.49 dd (8.3, 2.5)	106.6, CH
6′	7.33 s	105.5, CH_2_	7.13 d (8.3)	133.2, CH
7-OCH_3_	3.98 s	56.7, CH_3_		
3′-OCH_3_	3.97 s	57.2, CH_3_		
4′-OCH_3_			3.78 s	55.7, CH_3_
5′-OCH_3_	3.97 s	57.2, CH_3_		

**Table 4:** Results of the ACE-inhibitory assay.

Compound	Inhibition at 90 μM (%) ± SD	IC_50_ (μM) ± SD
**12**	35.4 ± 3.9	185.8 ± 12.8
**13**	95.5 ± 2.8	18.0 ± 1.2
**14**	106.7 ± 1.1	14.0 ± 0.7
**15**	96.7 ± 6.3	15.2 ± 0.4
**16**	102.8 ± 1.5	7.7 ± 0.9
**17**	98.5 ± 1.2	14.8 ± 0.8
**18**	98.7 ± 3.8	22.3 ± 0.9
**Captopril**	80.8 ± 1.2	0.2 ± 0.1

**Table 5:** Domain-specific inhibitory capacity of (–)-hopeaphenol (**16**) compared to BPPb (substrate concentration [S] = KM calculated from initial velocity studies of both substrates; Abz-SDK(Dnp)P-OH; [S] = 72 μM and Abz-LFK(Dnp)-OH; [S] = 21 μM).

	Inhibition (%) ± SD	
	N-Domain	C-Domain
BPPb (200 nM)	5.2 ± 0.2	83.20 ± 3.6
**16** (10 μM)	42.41 ± 0.8	18.17 ± 0.5
**16** (50 μM)	55.74 ± 3.2	23.39 ± 0.6

**Table 6:** Results of the antiproliferative assay (IC_50_ in μM ± SD).

	Colo 205	Colo 320
Compound	IC_50_ (μM) ± SD	IC_50_ (μM) ± SD
**12**	48.33 ± 1.54	40.4 ± 1.84
**13**	>100	>100
**14**	>100	>100
**15**	>100	>100
**16**	1.59 ± 0.11	6.08 ± 0.24
**17**	>100	>100
**18**	>100	>100
**DMSO**	>1%	>1%
**Cisplatin**	53.93 ± 3.92	64.68 ± 3.56
**Doxorubicin**	0.33 ± 0.03	0.67 ± 0.06

## Data Availability

The original contributions presented in the study are included in the article/Supplementary Materials, further inquiries can be directed to the corresponding author (A.V.).

## Supplementary Materials

The following supporting information can be downloaded at: https://www.mdpi.com/article/10.3390/molecules29143427/s1, Figures S1–S73: 1D and 2D NMR, and HRESIMS spectra of compounds **1**–**5**, ^1^H and JMOD spectra of **6**–**23**, and ^1^H NMR spectra of **24**–**26**.
