# Supplementary material for: Phytochemical Investigation of Carex praecox Schreb. and ACE-Inhibitory Activity of Oligomer Stilbenes of the Plant
Source: Molecules. 2024 Jul 22;29(14):3427. doi: 10.3390/molecules29143427 (PMC11280411; doi:10.3390/molecules29143427)
Supplement: Supplementary file 1 [file molecules-29-03427-s001.zip › molecules-3092299-supplementary.pdf]

Supporting Information

Phytochemical Investigation of *Carex praecox* Schreb. and ACE-Inhibitory Activity of Oligomer Stilbenes of the Plant

Zsuzsanna Csilla Dávid [1], Norbert Kúsz [1], Orinamhe Godwin Agbadua [1], Róbert Berkecz [2], Annamária Kincses [1,3], Gabriella Spengler [4], Attila Hunyadi [1,3], Judit Hohmann [1,3] and Andrea Vasas [1,3,*]

1 Department of Pharmacognosy, University of Szeged, 6720 Szeged, Hungary; davidzsuzsanna88@gmail.com (Z.C.D.); kusznorbert@gmail.com (N.K.); orinamhe.agbadua@uniben.edu (O.G.A.); kincses.annamaria@szte.hu (A.K.); hunyadi.attila@szte.hu (A.H.); hohmann.judit@szte.hu (J.H.)
2 Institute of Pharmaceutical Analysis, University of Szeged, 6720 Szeged, Hungary; berkecz.robert@szte.hu
3 HUN-REN-USZ Biologically Active Natural Products Research Group, University of Szeged, Eötvös u. 6, 6720 Szeged, Hungary
4 Department of Medical Microbiology, Albert Szent-Györgyi Health Center, Albert Szent-Györgyi Medical School, University of Szeged, 6725 Szeged, Hungary; spengler.gabriella@med.u-szeged.hu
* Correspondence: vasas.andrea@szte.hu

Table of Contents

Figure S1. Page S6
^{1}H-NMR spectrum of compound **1** (500 MHz, CD_3OD)

Figure S2. Page S6
JMOD spectrum of compound **1** (125 MHz, CD_3OD)

Figure S3. Page S7
HSQC spectrum of compound **1**

Figure S4. Page S7
^{1}H-^{1}H-COSY spectrum of compound **1**

Figure S5. Page S8
HMBC spectrum of compound **1**

Figure S6. Page S8
NOESY spectrum of compound **1**

Figure S7. Page S9
HRESIMS spectrum of compound **1**

Figure S8. Page S9
^{1}H-NMR spectrum of compound **2** (500 MHz, CD_3OD)

Figure S9. Page S10
JMOD spectrum of compound **2** (125 MHz, CD_3OD)

Figure S10. Page S10
HSQC spectrum of compound **2**

Figure S11. Page S11
^{1}H-^{1}H-COSY spectrum of compound **2**

Figure S12. Page S11
HMBC spectrum of compound **2**

Figure S13. Page S12
NOESY spectrum of compound **2**

Figure S14. Page S12
HRESIMS spectrum of compound **2**

Figure S15. Page S13
^{1}H-NMR spectrum of compound **3** (500 MHz, CD_3OD)

Figure S16. Page S13
JMOD spectrum of compound **3** (125 MHz, CD_3OD)

Figure S17. Page S14
HSQC spectrum of compound **3**

Figure S18. Page S14
^{1}H-^{1}H-COSY spectrum of compound **3**

Figure S19. Page S15
HMBC spectrum of compound **3**

Figure S20. Page S15
NOESY spectrum of compound **3**

Figure S21. Page S16
^{1}H-NMR spectrum of compound **4** (500 MHz, CD_3OD)

Figure S22. Page S16
JMOD spectrum of compound **4** (125 MHz, CD_3OD)

Figure S23. Page S17
HSQC spectrum of compound **4**

Figure S24. Page S17
^{1}H-^{1}H-COSY spectrum of compound **4**

Figure S25. Page S18
HMBC spectrum of compound **4**

Figure S26. Page S18
NOESY spectrum of compound **4**

Figure S27. Page S19
HRESIMS spectrum of compound **4**

Figure S28. Page S19
^{1}H-NMR spectrum of compound **5** (500 MHz, CD_3OD)

Figure S29. Page S20
JMOD spectrum of compound **5** (125 MHz, CD_3OD)

Figure S30. Page S20
HSQC spectrum of compound **5**

Figure S31. Page S21
^{1}H-^{1}H-COSY spectrum of compound **5**

Figure S32. Page S21
HMBC spectrum of compound **5**

Figure S33. Page S22
NOESY spectrum of compound **5**

Figure S34. Page S22
HRESIMS spectrum of compound **5**

Figure S35. Page S23
^{1}H-NMR spectrum of compound **6** (500 MHz, CD_3OD)

Figure S36. Page S23
JMOD spectrum of compound **6** (125 MHz, CD_3OD)

Figure S37. Page S24
^{1}H-NMR spectrum of compound **7** (500 MHz, CD_3OD)

Figure S38. Page S24
JMOD spectrum of compound **7** (125 MHz, CD_3OD)

Figure S39. Page S25
^{1}H-NMR spectrum of compound **8** (500 MHz, CD_3OD)

Figure S40. Page S25
JMOD spectrum of compound **8** (125 MHz, CD_3OD)

Figure S41. Page S26
^{1}H-NMR spectrum of compound **9** (500 MHz, CD_3OD)

Figure S42. Page S26
JMOD spectrum of compound **9** (125 MHz, CD_3OD)

Figure S43. Page S27
^{1}H-NMR spectrum of compound **10** (500 MHz, DMSO-d_6)

Figure S44. Page S27
JMOD spectrum of compound **10** (125 MHz, DMSO-d_6)

Figure S45. Page S28
^{1}H-NMR spectrum of compound **11** (500 MHz, CD_3OD)

Figure S46. Page S28
JMOD spectrum of compound **11** (125 MHz, CD_3OD)

Figure S47. Page S29
^{1}H-NMR spectrum of compound **12** (500 MHz, CD_3OD)

Figure S48. Page S29
JMOD spectrum of compound **12** (125 MHz, CD_3OD)

Figure S49. Page S30
^{1}H-NMR spectrum of compound **13** (500 MHz, CD_3OD)

Figure S50. Page S30
JMOD spectrum of compound **13** (125 MHz, CD_3OD)

Figure S51. Page S31
^{1}H-NMR spectrum of compound **14** (500 MHz, CD_3OD)

Figure S52. Page S31
JMOD spectrum of compound **14** (125 MHz, CD_3OD)

Figure S53. Page S32
^{1}H-NMR spectrum of compound **15** (500 MHz, CD_3OD)

Figure S54. Page S32
JMOD spectrum of compound **15** (125 MHz, CD_3OD)

Figure S55. Page S33
^{1}H-NMR spectrum of compound **16** (500 MHz, CD_3OD)

Figure S56. Page S33
JMOD spectrum of compound **16** (125 MHz, CD_3OD)

Figure S57. Page S34
^{1}H-NMR spectrum of compound **17** (500 MHz, CD_3OD)

Figure S58. Page S34
JMOD spectrum of compound **17** (125 MHz, CD_3OD)

Figure S59. Page S35
^{1}H-NMR spectrum of compound **18** (500 MHz, CD_3OD)

Figure S60. Page S35
JMOD spectrum of compound **18** (125 MHz, CD_3OD)

Figure S61. Page S36
^{1}H-NMR spectrum of compound **19** (500 MHz, CD_3OD)

Figure S62. Page S36
JMOD spectrum of compound **19** (125 MHz, CD_3OD)

Figure S63. Page S37
^{1}H-NMR spectrum of compound **20** (500 MHz, CD_3OD)

Figure S64. Page S37
JMOD spectrum of compound **20** (125 MHz, CD_3OD)

Figure S65. Page S38
^{1}H-NMR spectrum of compound **21** (500 MHz, CD_3OD)

Figure S66. Page S38
JMOD spectrum of compound **21** (125 MHz, CD_3OD)

Figure S67. Page S39
^{1}H-NMR spectrum of compound **22** (500 MHz, CD_3OD)

Figure S68. Page S39
JMOD spectrum of compound **22** (125 MHz, CD_3OD)

Figure S69. Page S40
^{1}H-NMR spectrum of compound **23** (500 MHz, $CDCl_3$)

Figure S70. Page S40
JMOD spectrum of compound **23** (125 MHz, $CDCl_3$)

Figure S71. Page S41
^{1}H-NMR spectrum of compound **24** (500 MHz, CD_3OD)

Figure S72. Page S41
^{1}H-NMR spectrum of compound **25** (500 MHz, CD_3OD)

Figure S73. Page S42
^{1}H-NMR spectrum of compound **26** (500 MHz, CD_3OD)

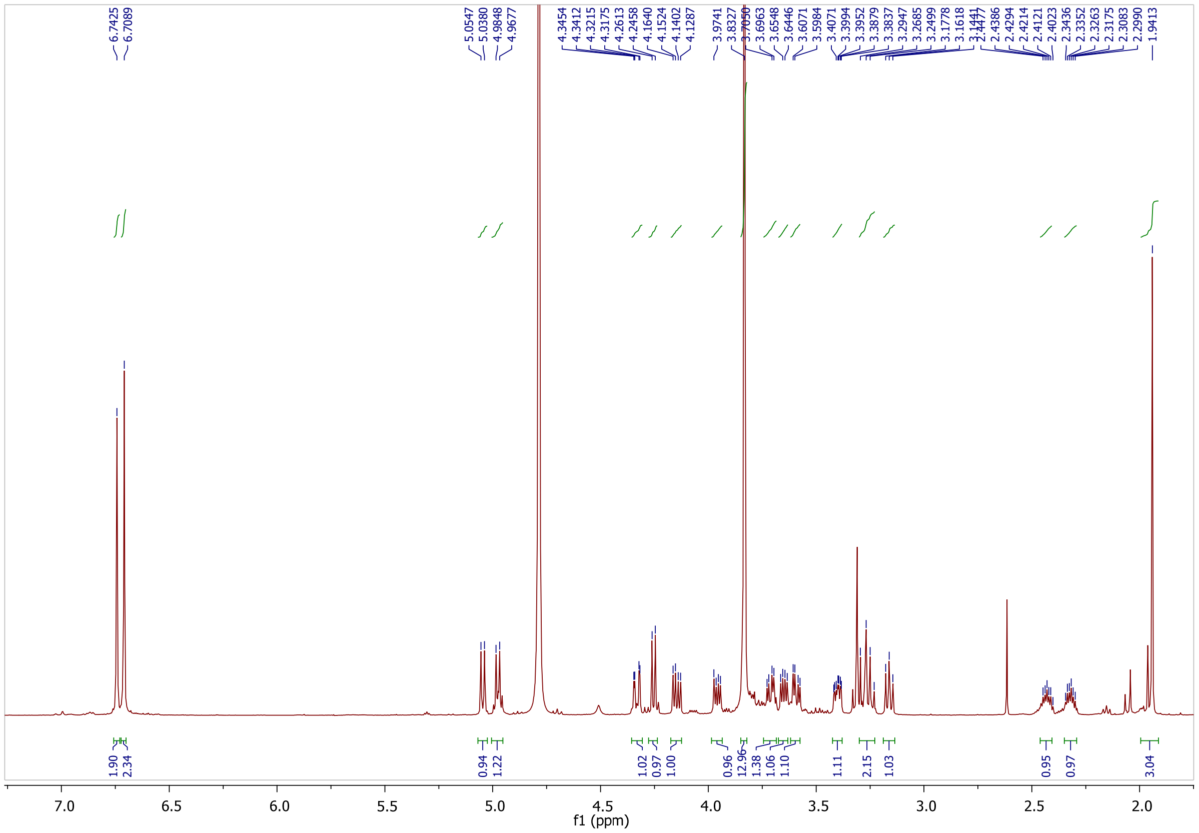

Figure S1. ^{1}H-NMR spectrum of compound **1** (500 MHz, CD_3OD)

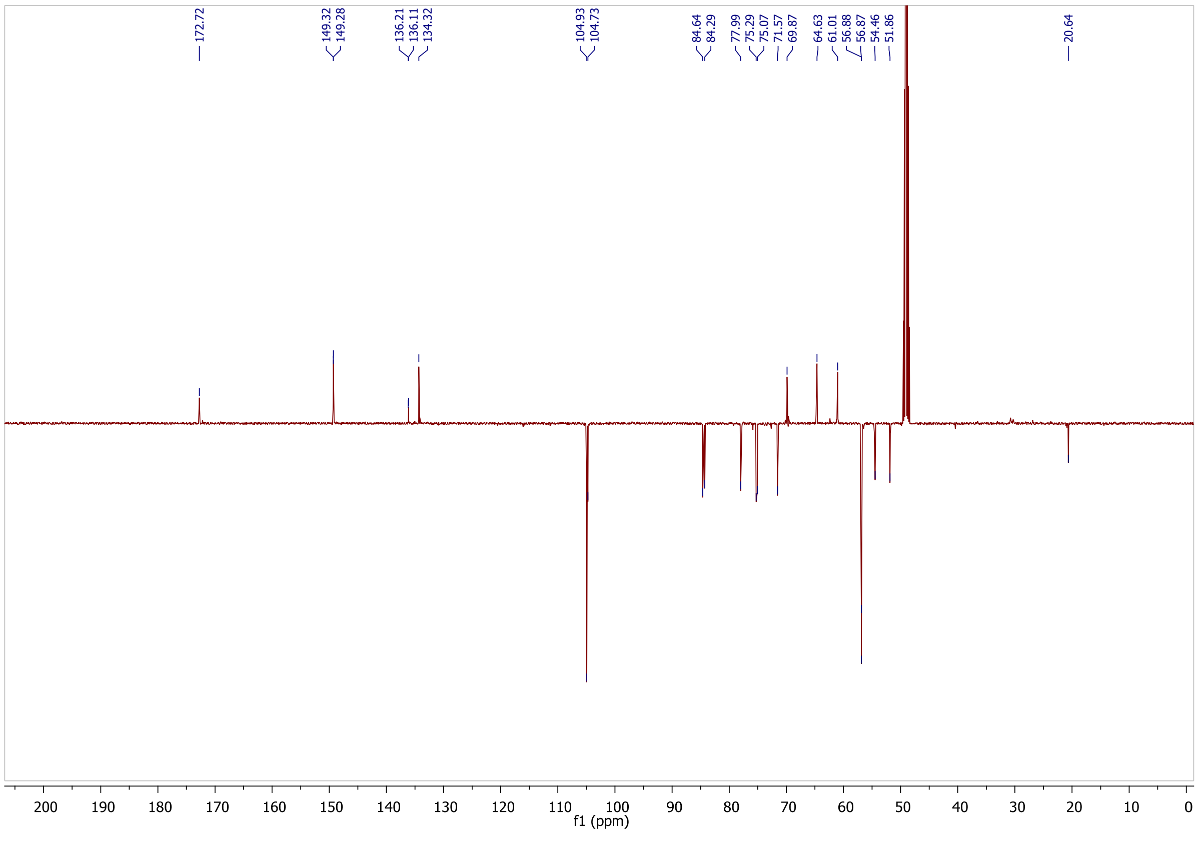

Figure S2. JMOD spectrum of compound **1** (125 MHz, CD_3OD)

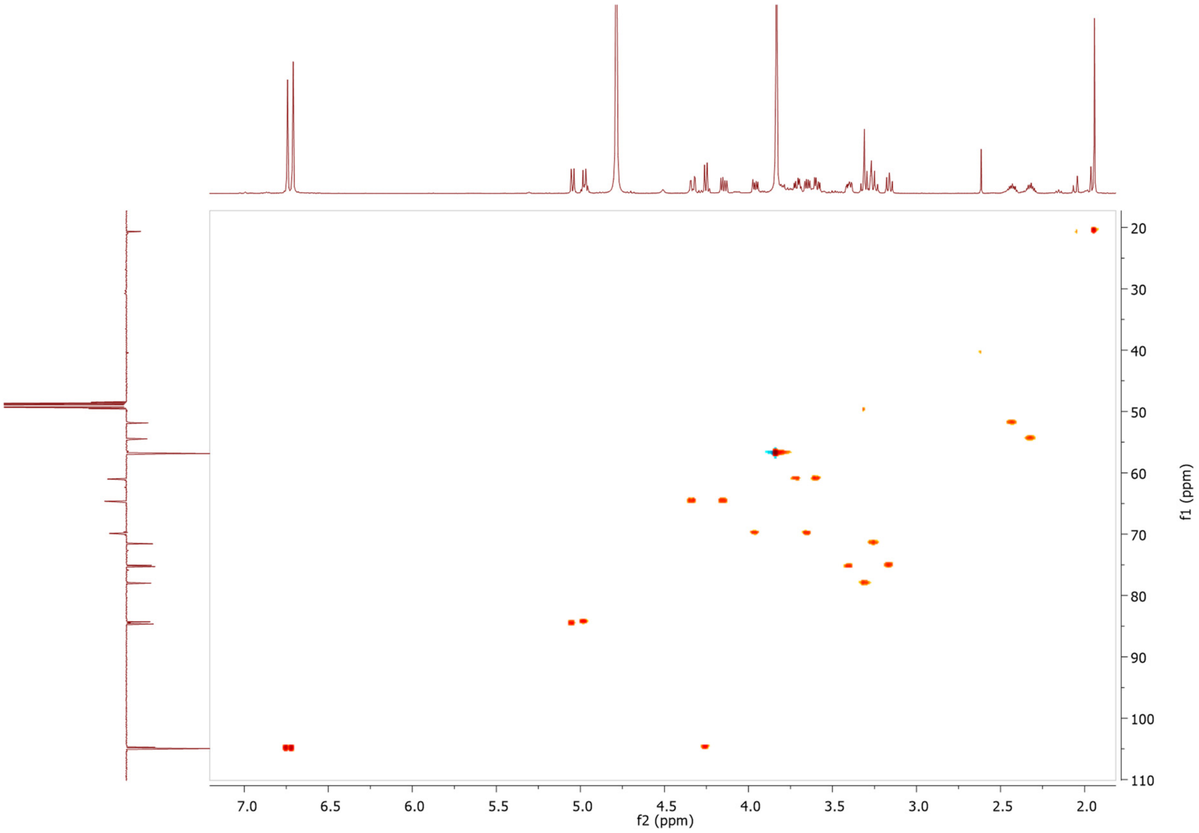

Figure S3. HSQC spectrum of compound **1**

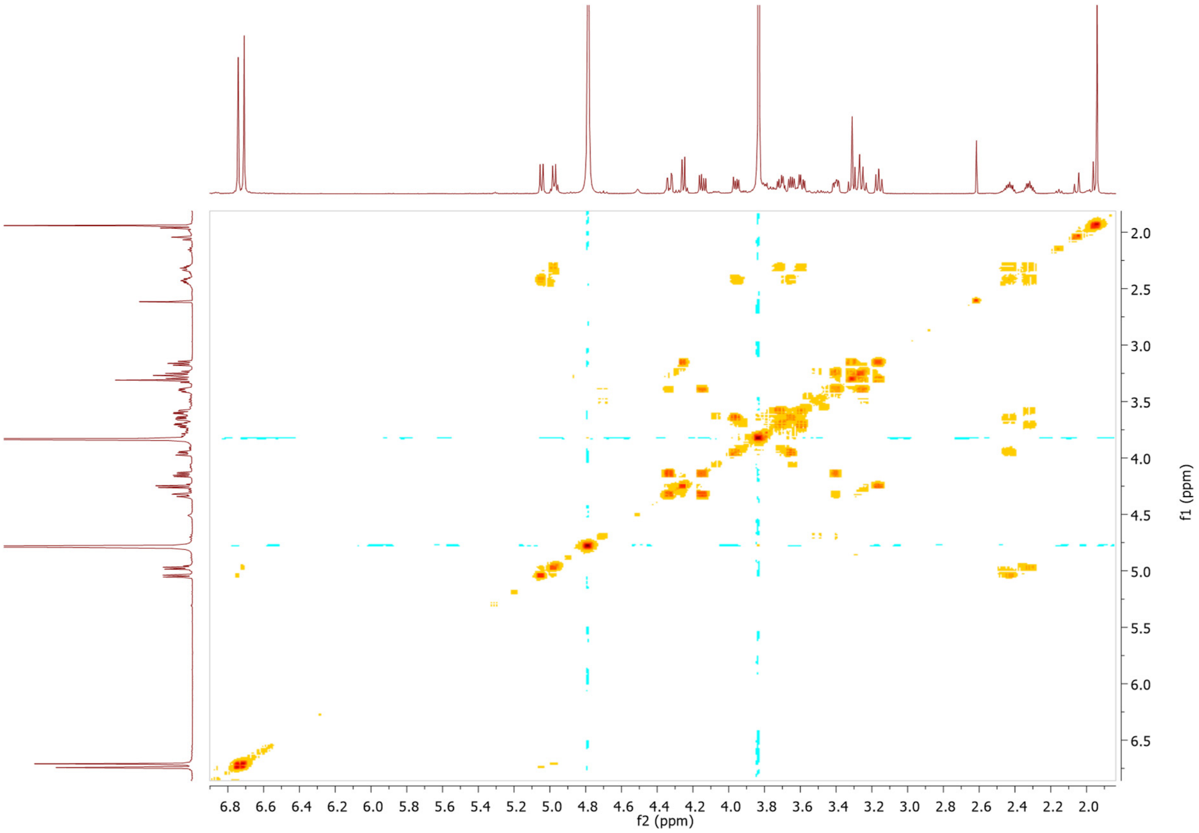

Figure S4. ¹H-¹H-COSY spectrum of compound **1**

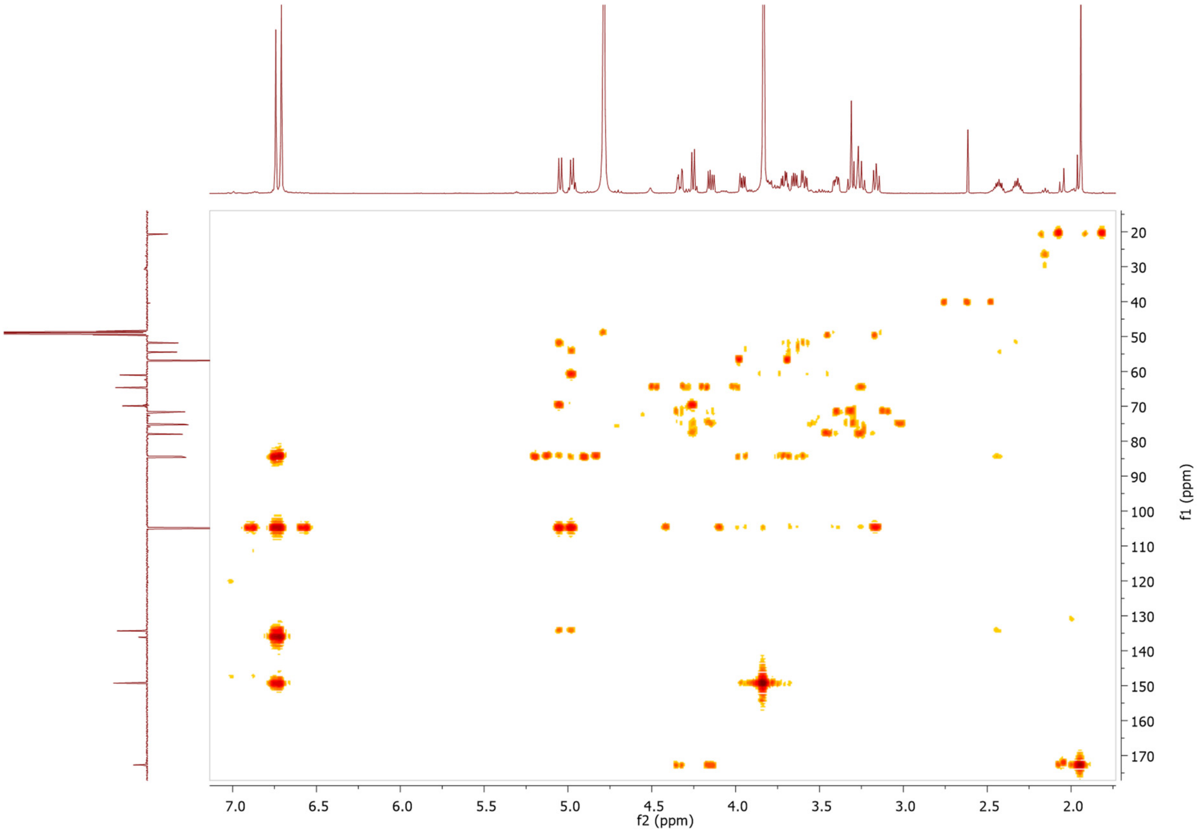

Figure S5. HMBC spectrum of compound **1**

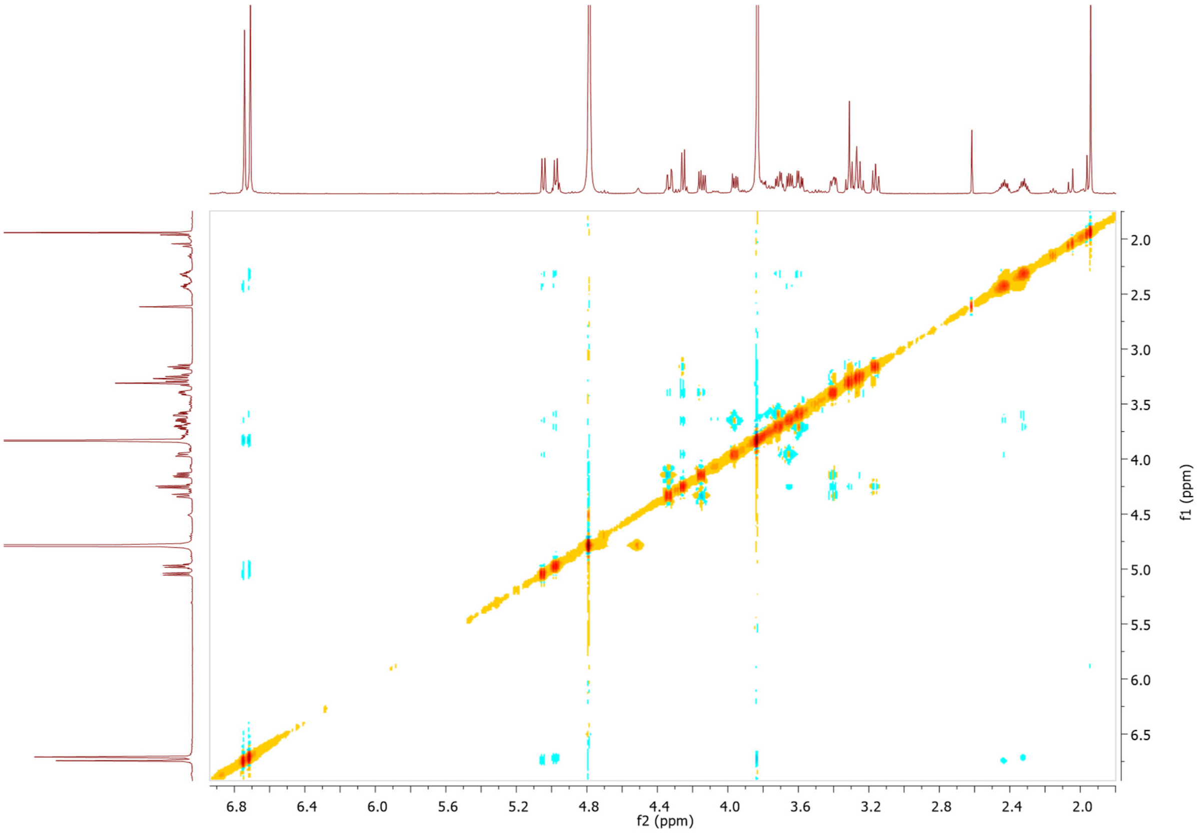

Figure S6. NOESY spectrum of compound **1**

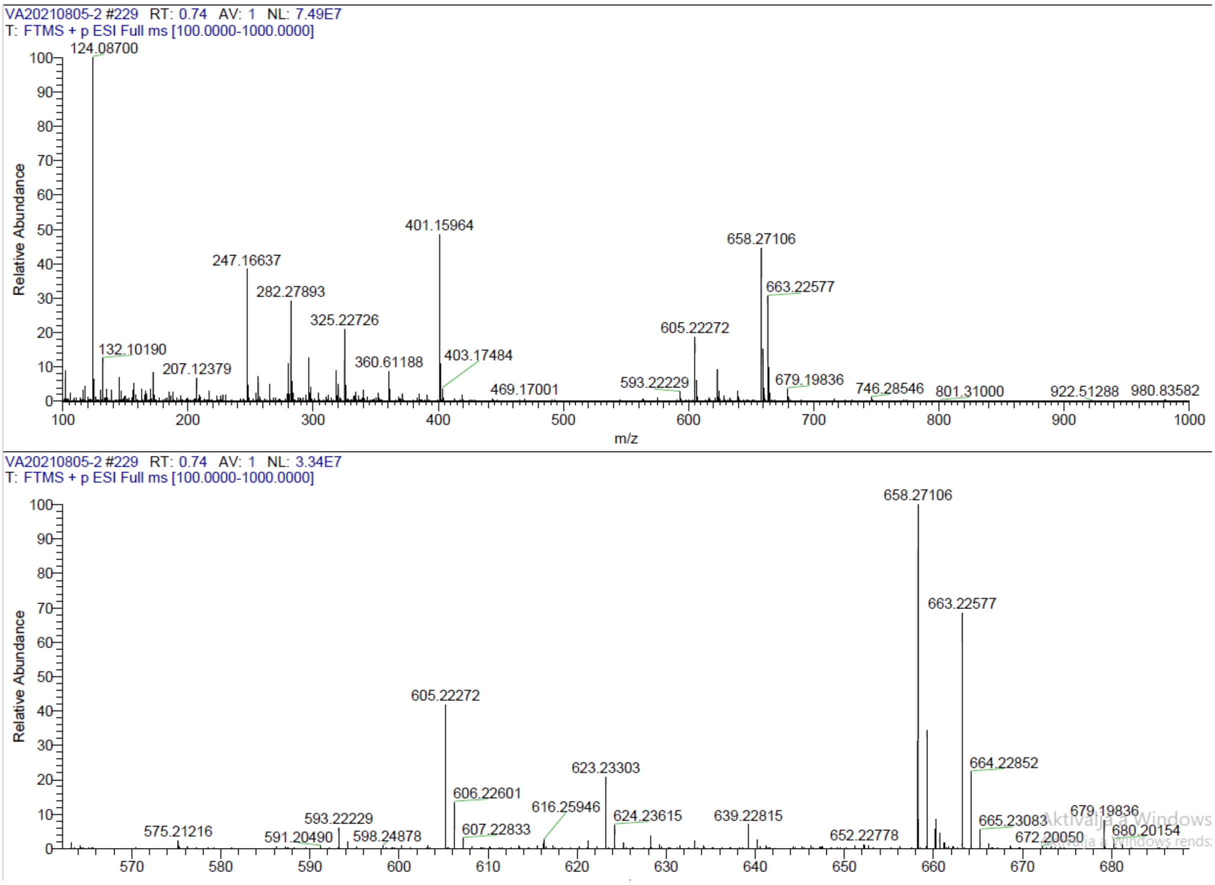

Figure S7. HRESIMS spectrum of compound **1**

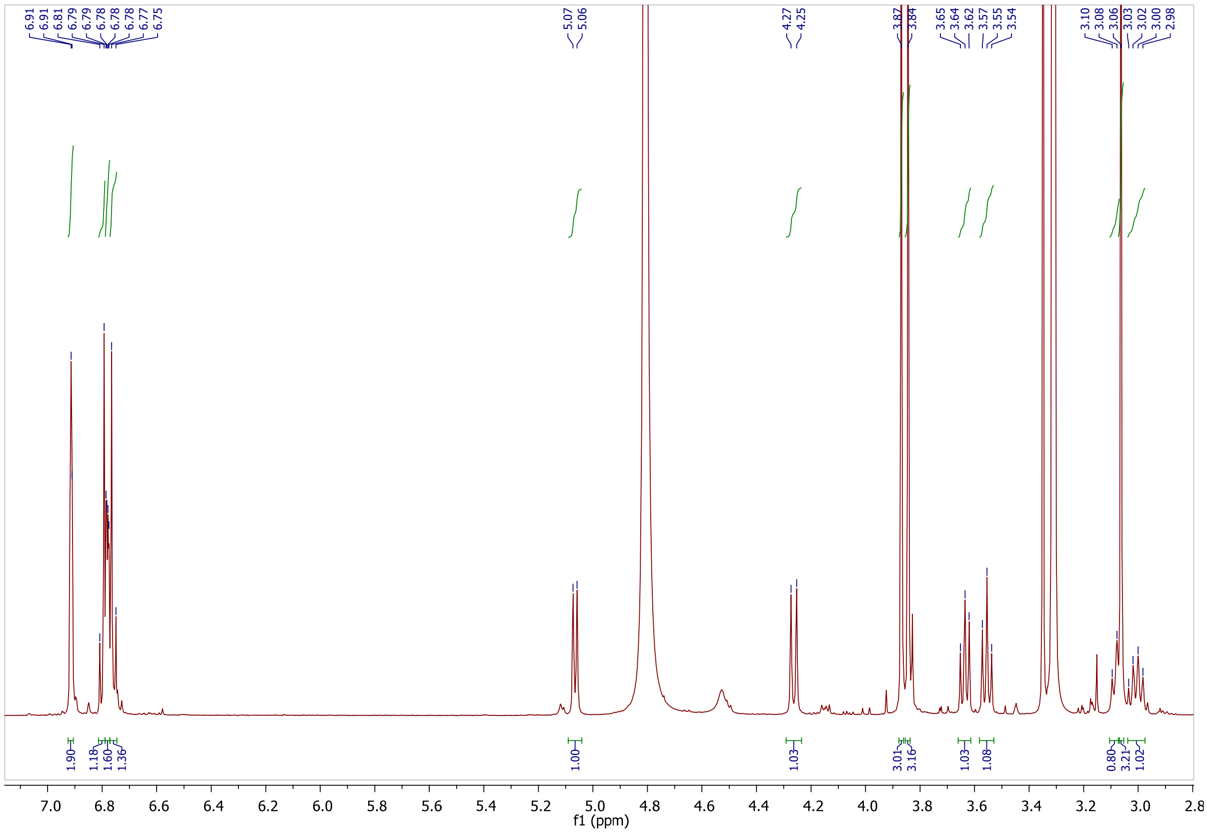

Figure S8. ^{1}H-NMR spectrum of compound **2** (500 MHz, CD_3OD)

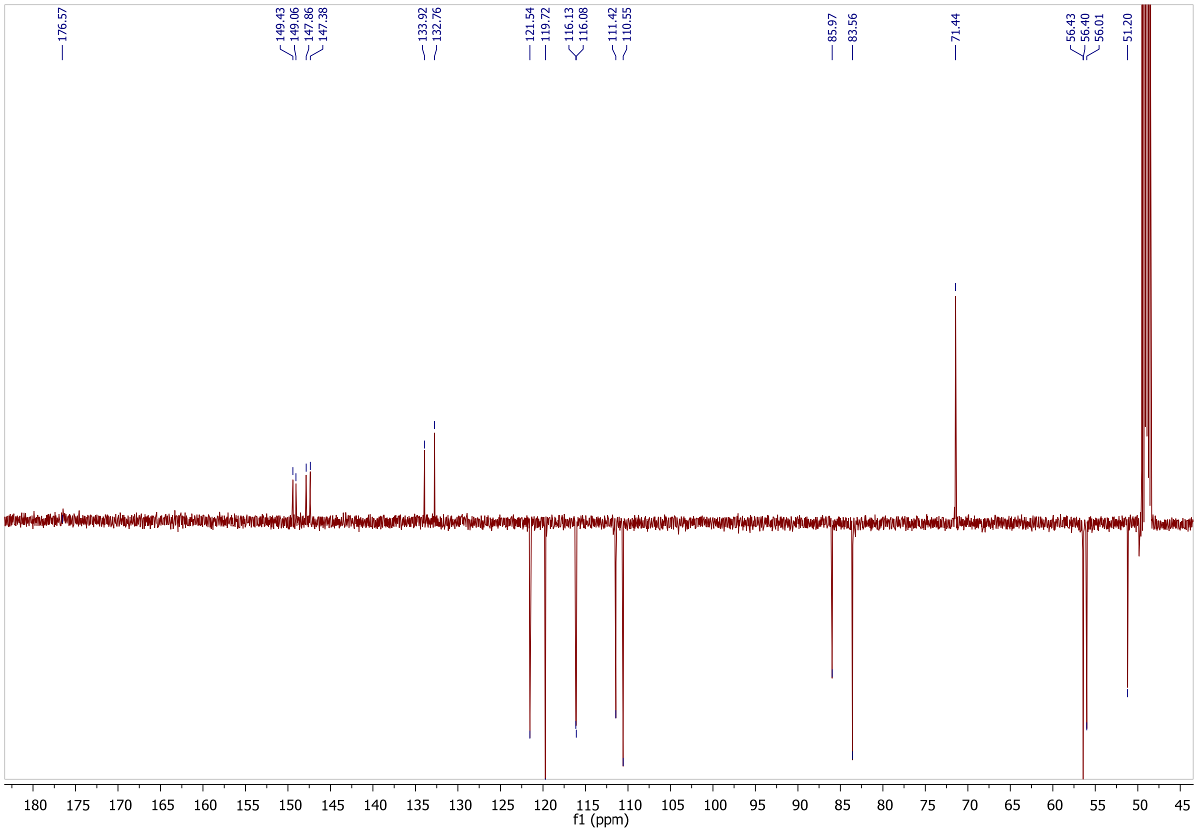

Figure S9. JMOD spectrum of compound **2** (125 MHz, CD_3OD)

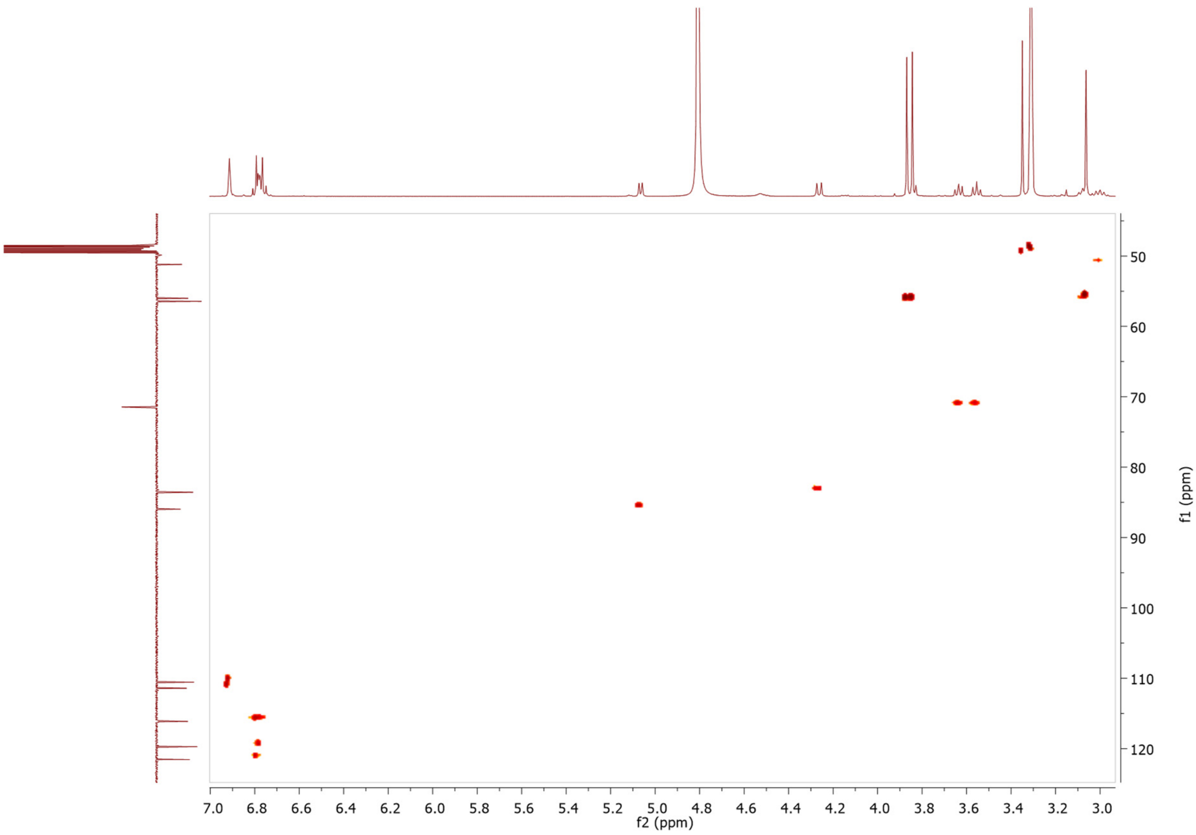

Figure S10. HSQC spectrum of compound **2**

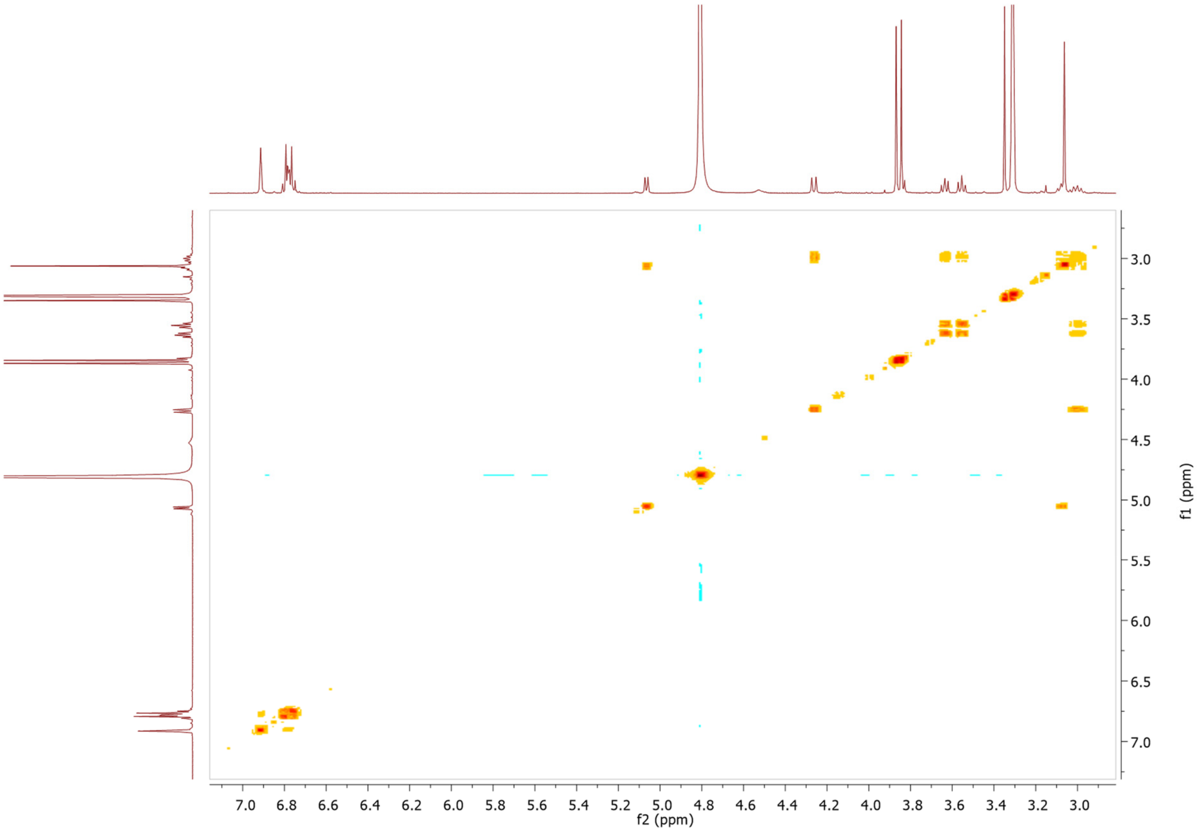

Figure S11. ^{1}H-^{1}H-COSY spectrum of compound **2**

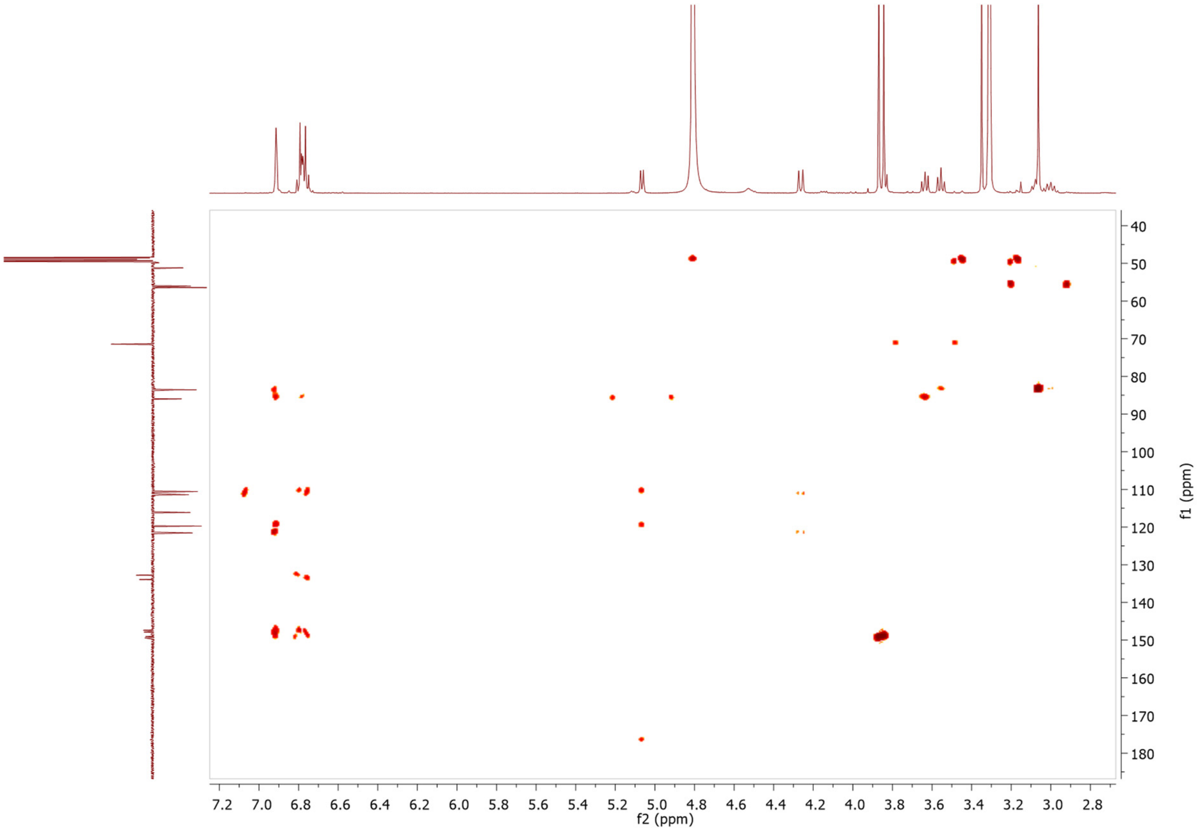

Figure S12. HMBC spectrum of compound **2**

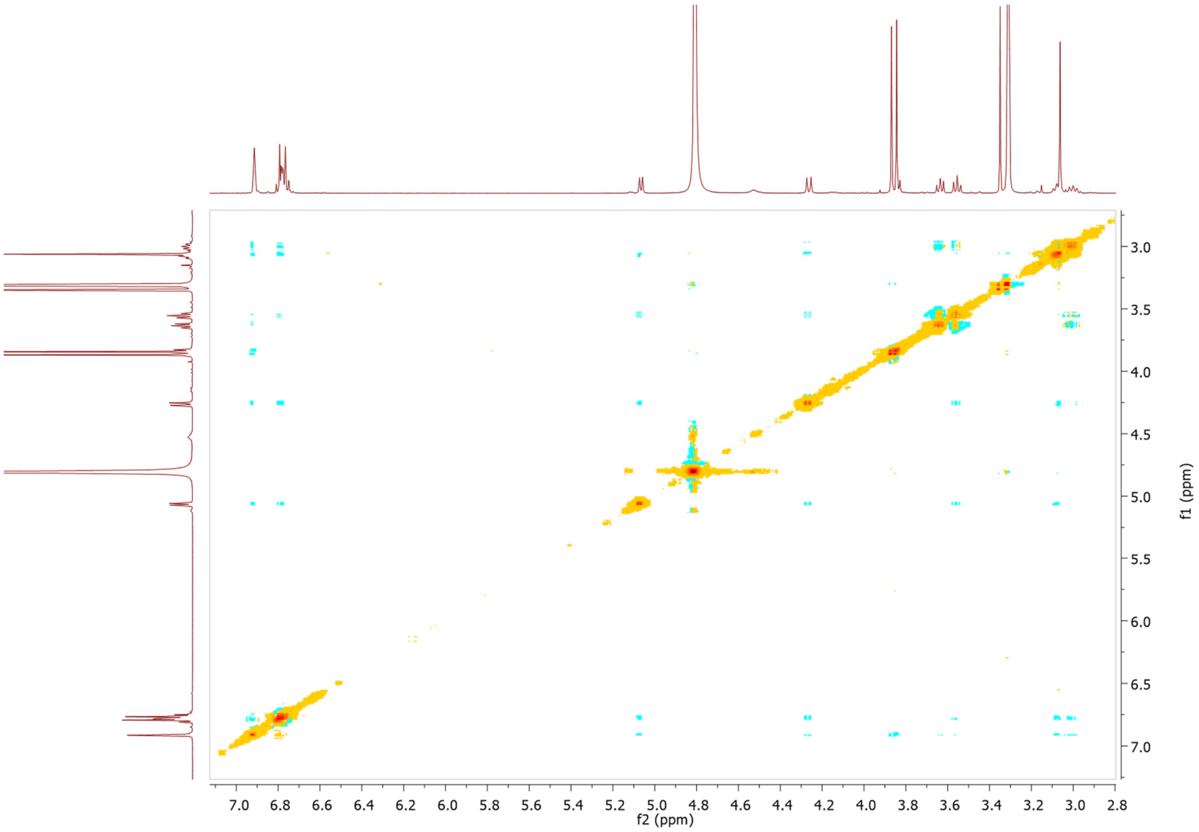

Figure S13. NOESY spectrum of compound **2**

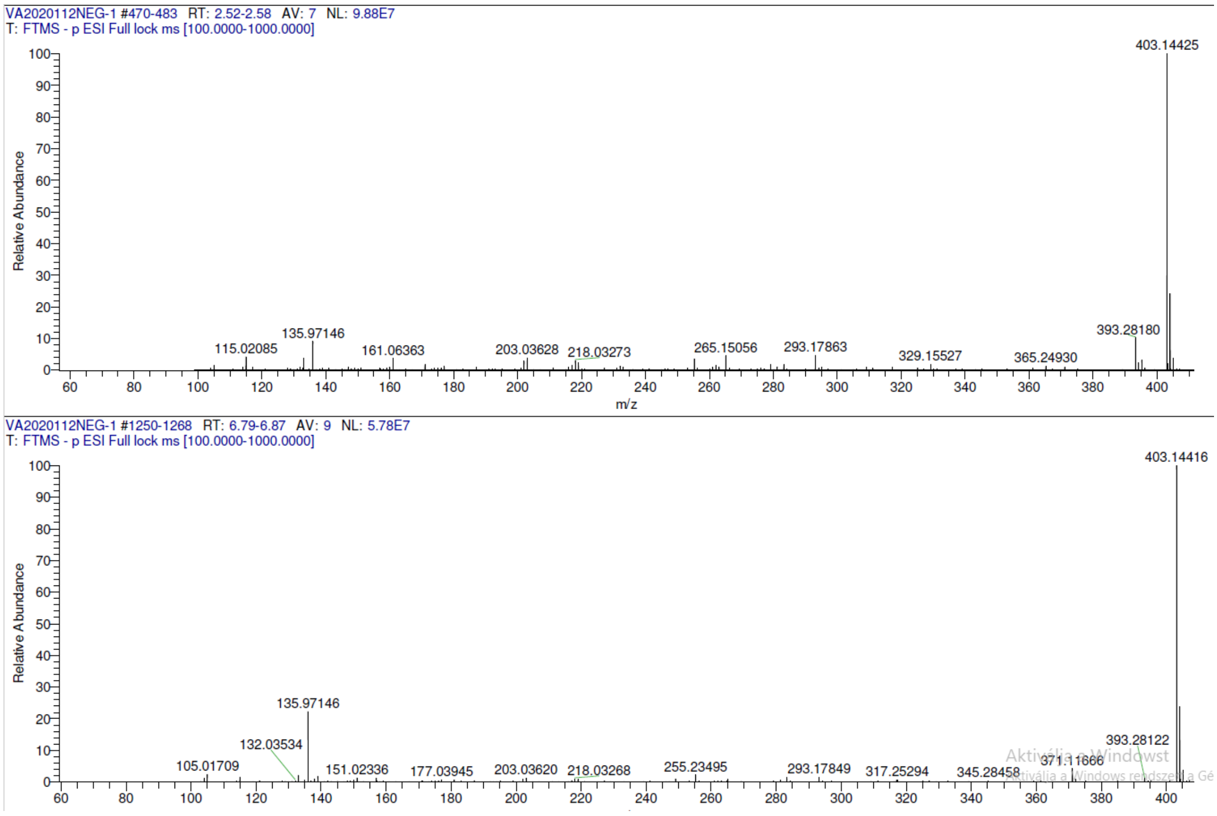

Figure S14. HRESIMS spectrum of compound **2**

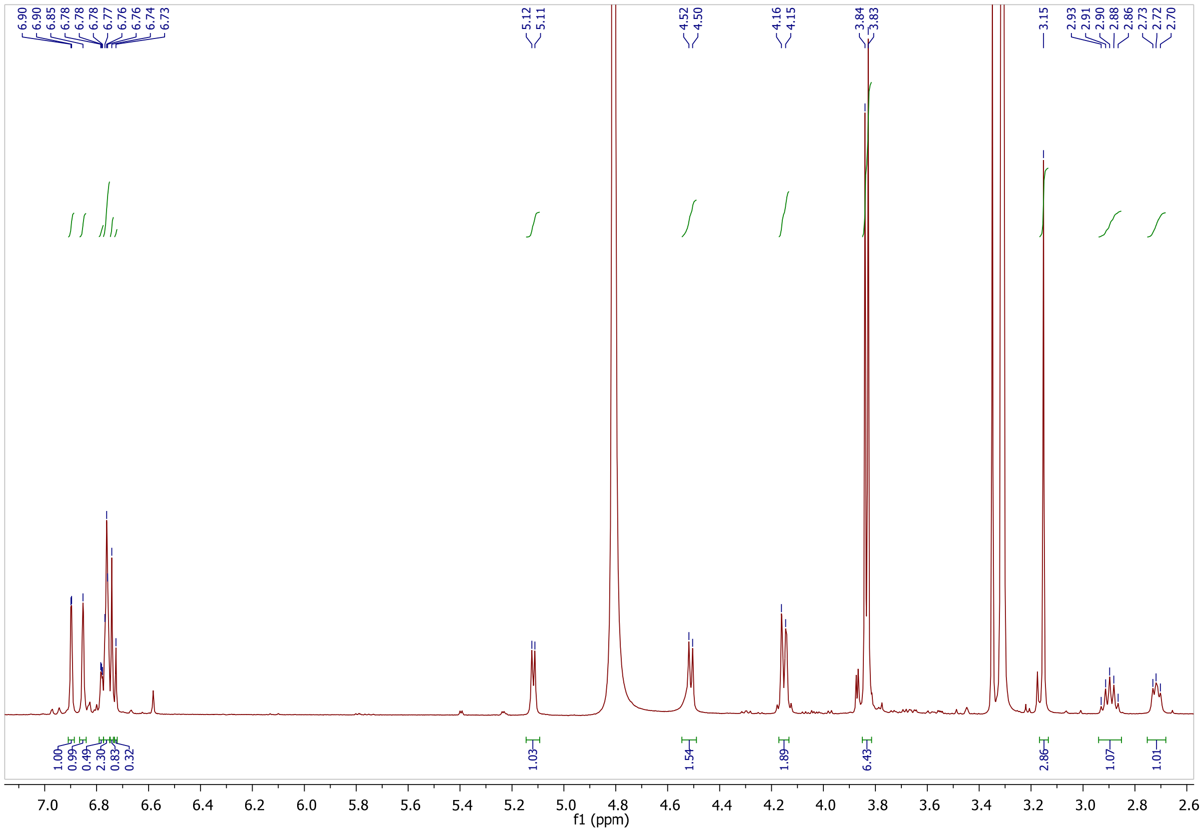

Figure S15. ^{1}H-NMR spectrum of compound **3** (500 MHz, CD_3OD)

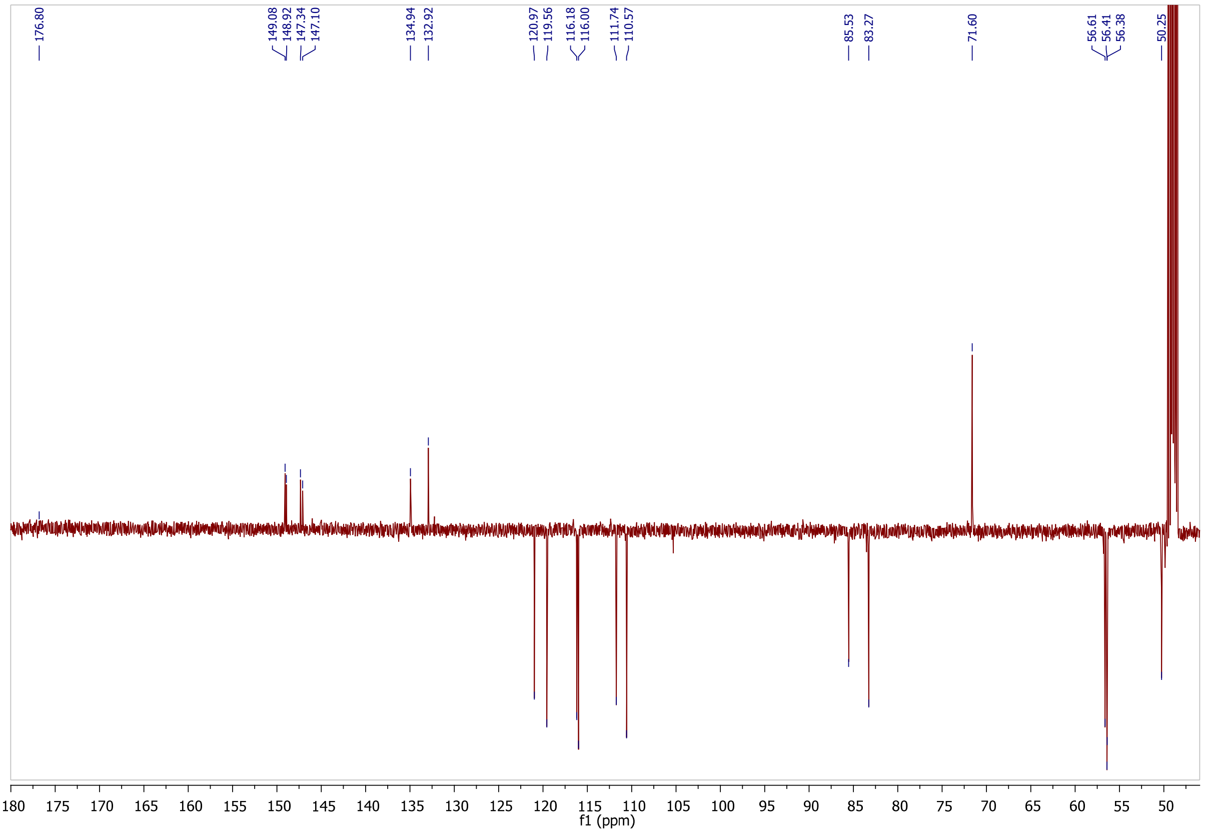

Figure S16. JMOD spectrum of compound **3** (125 MHz, CD_3OD)

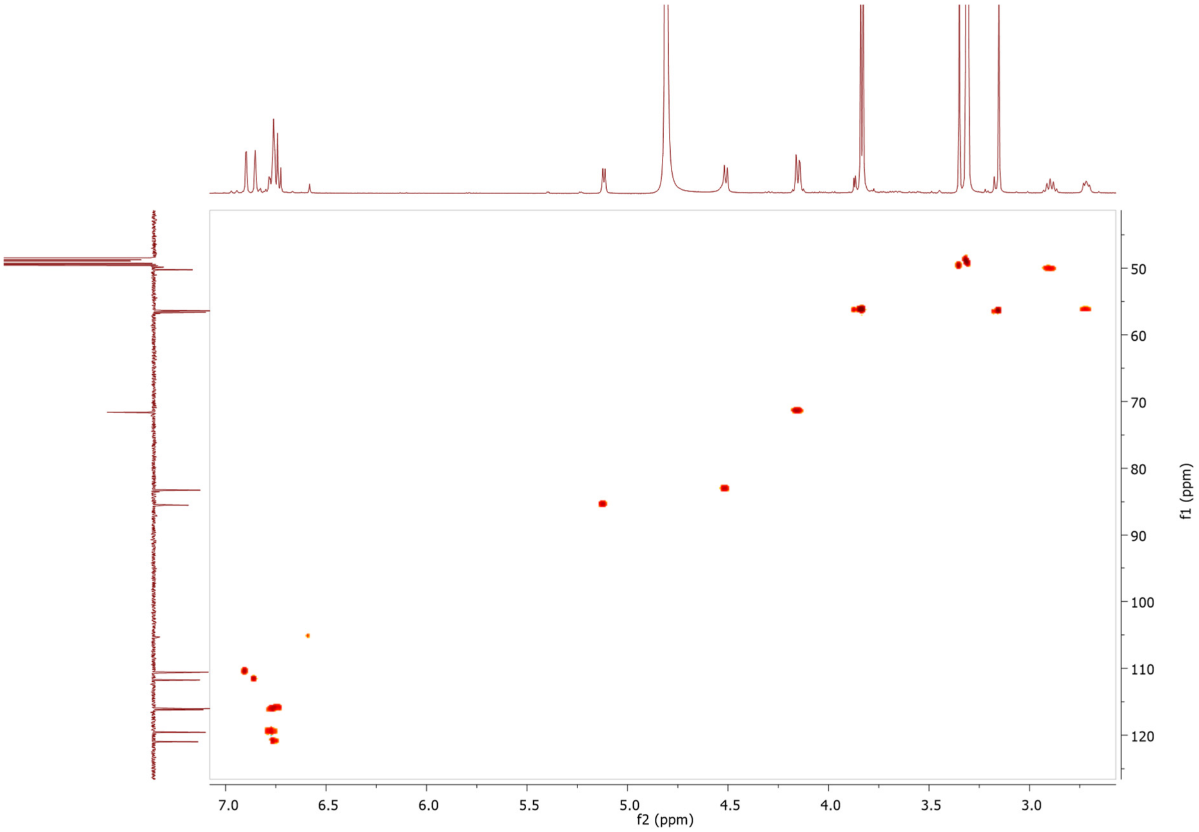

Figure S17. HSQC spectrum of compound **3**

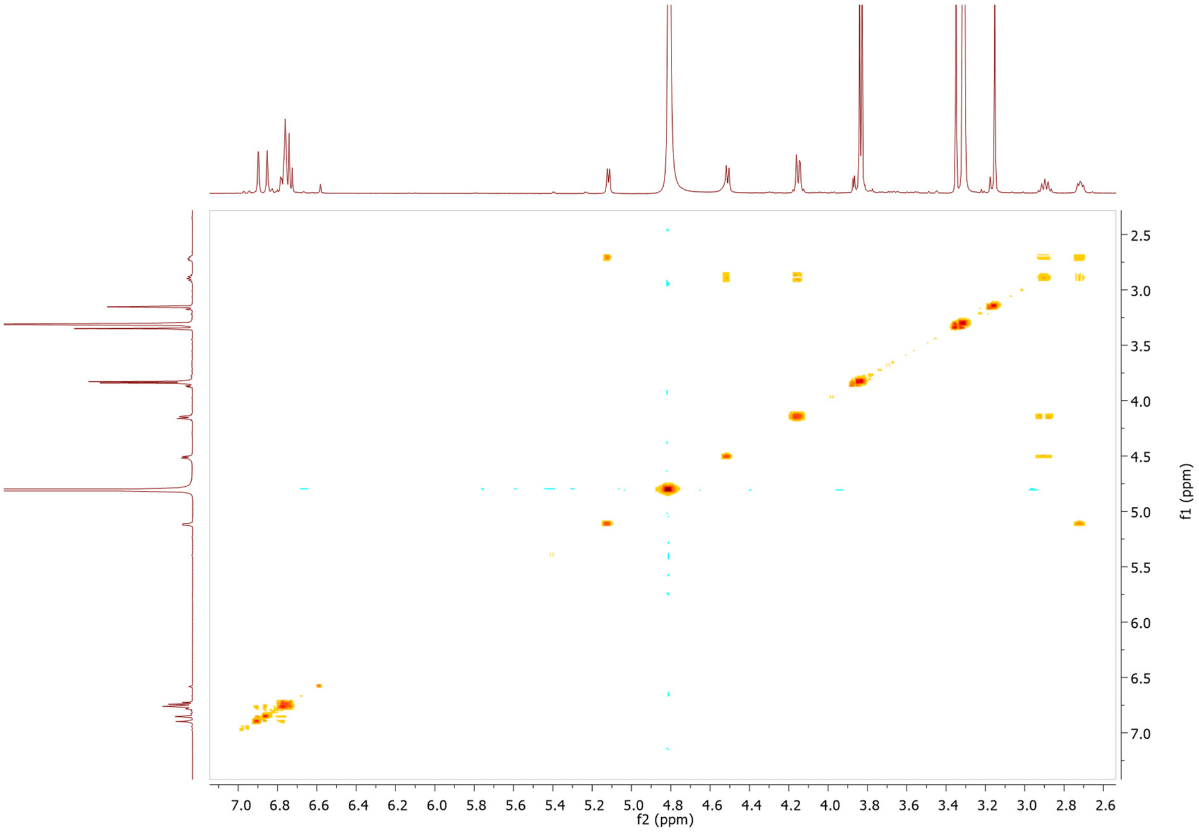

Figure S18. ^{1}H-^{1}H-COSY spectrum of compound **3**

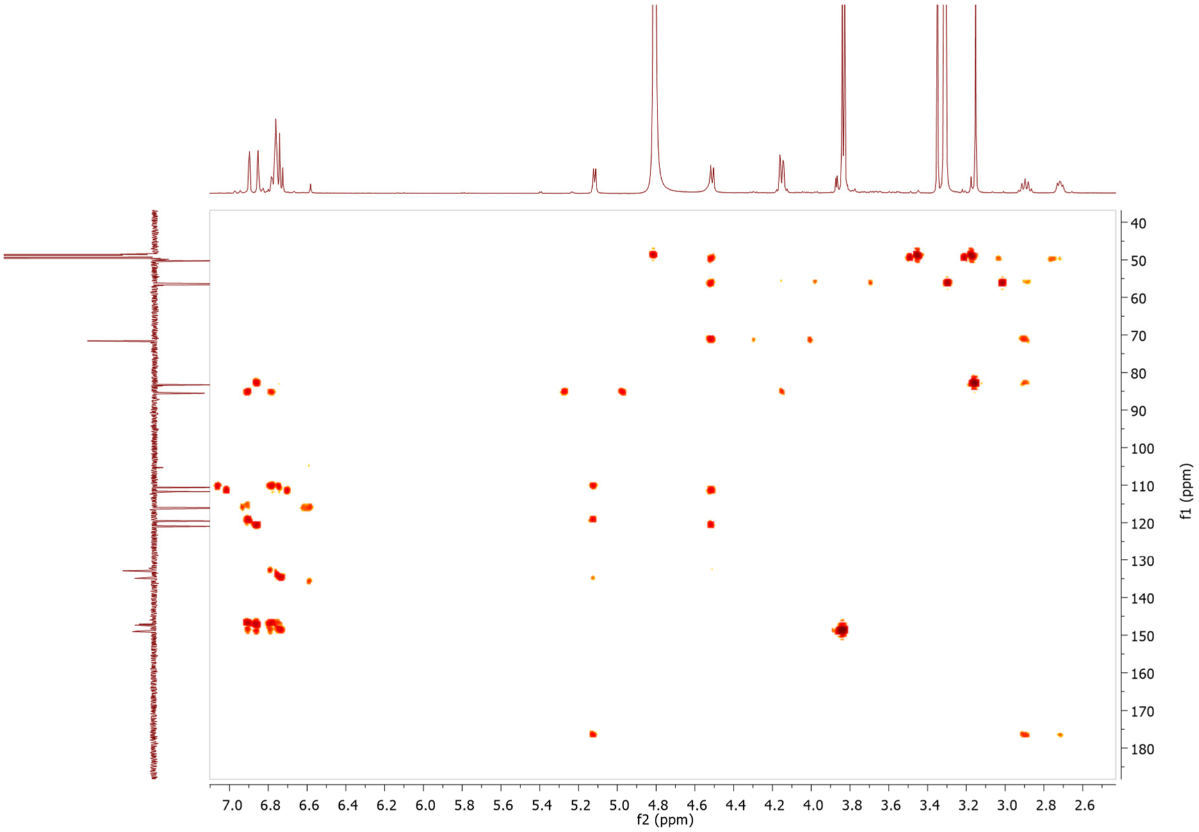

Figure S19. HMBC spectrum of compound **3**

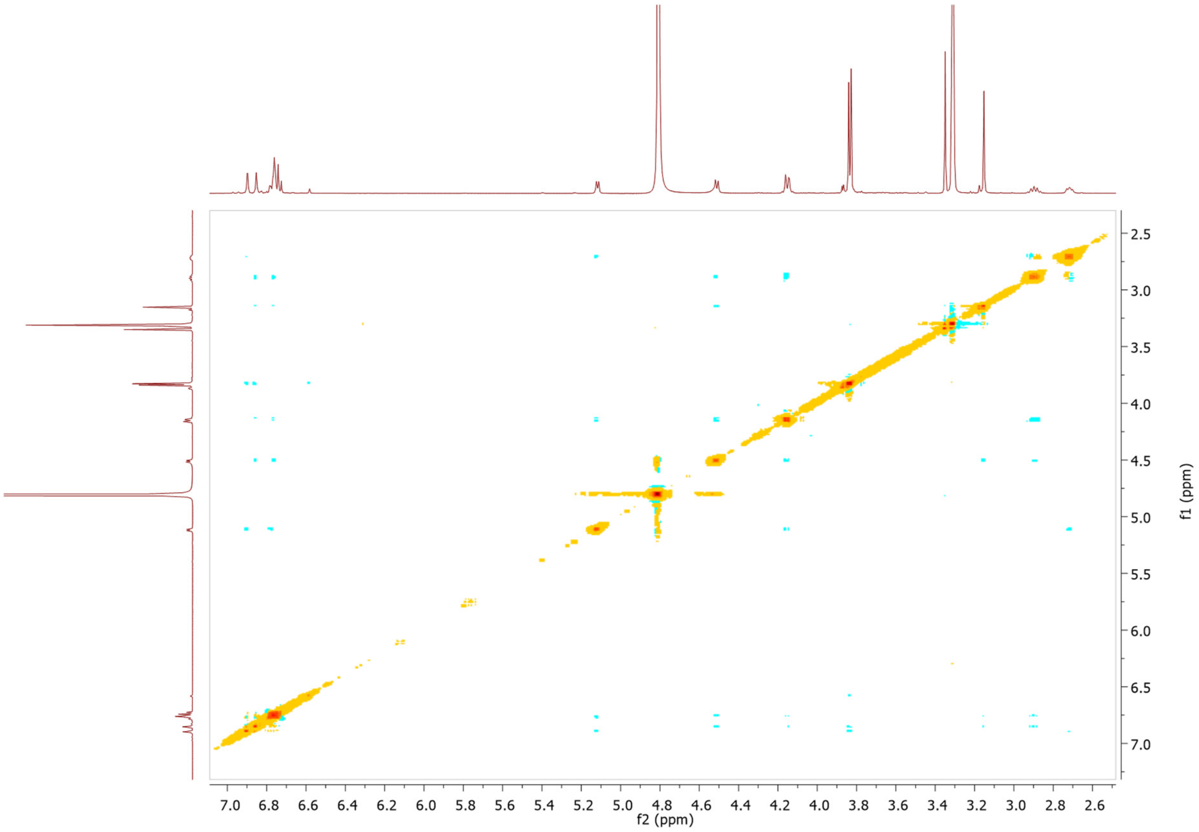

Figure S20. NOESY spectrum of compound **3**

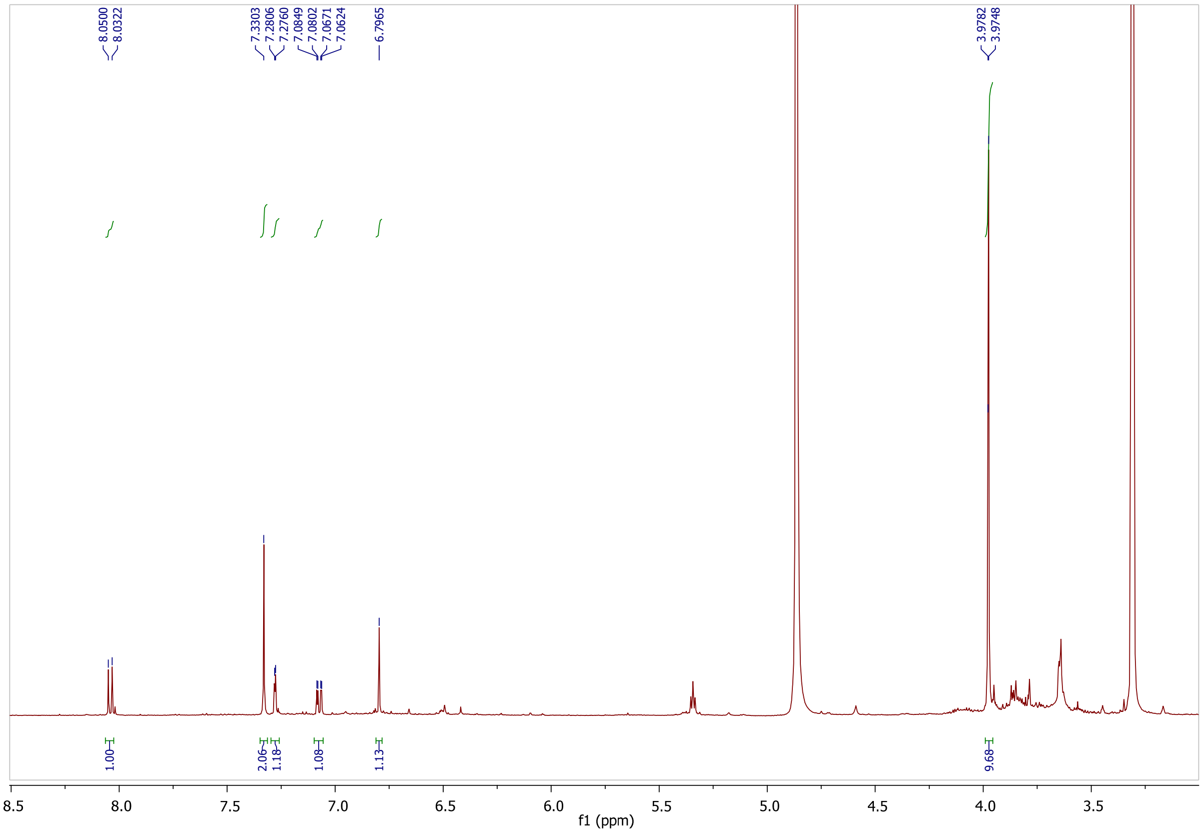

Figure S21. ^{1}H-NMR spectrum of compound **4** (500 MHz, CD_3OD)

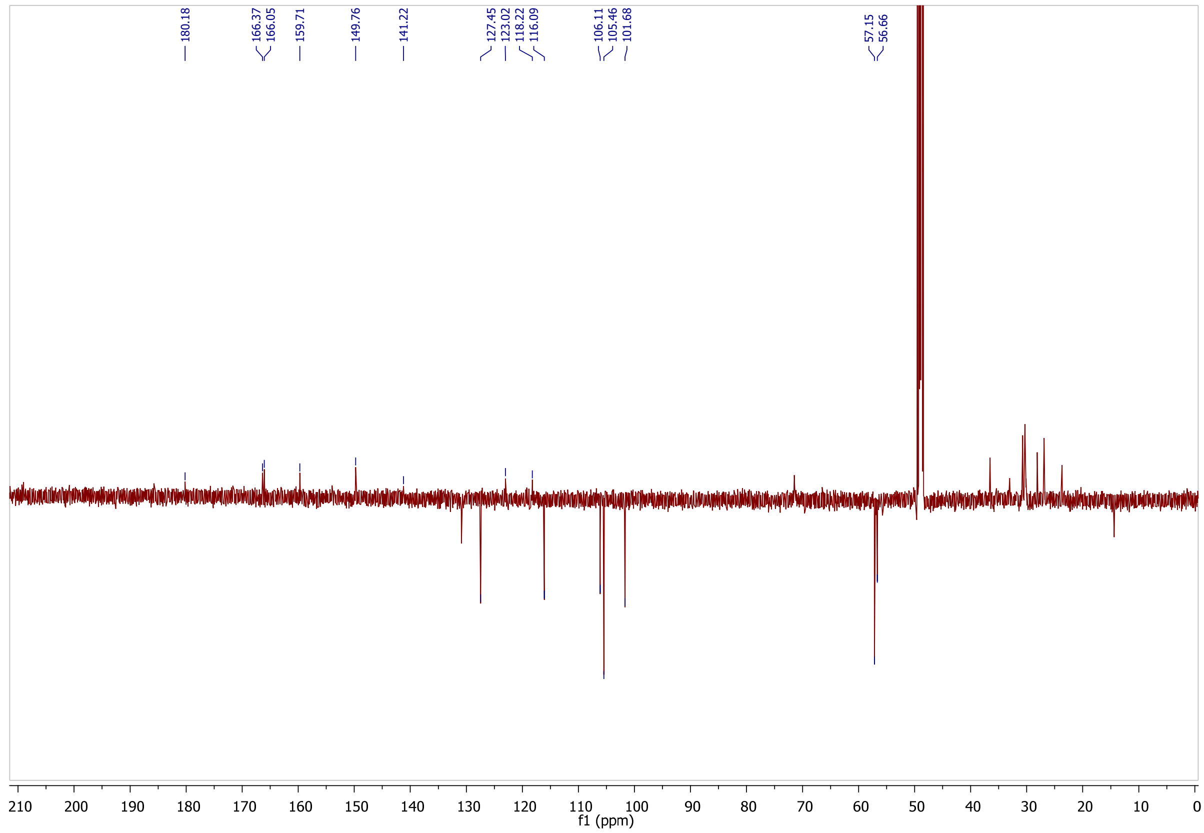

Figure S22. JMOD spectrum of compound **4** (125 MHz, CD_3OD)

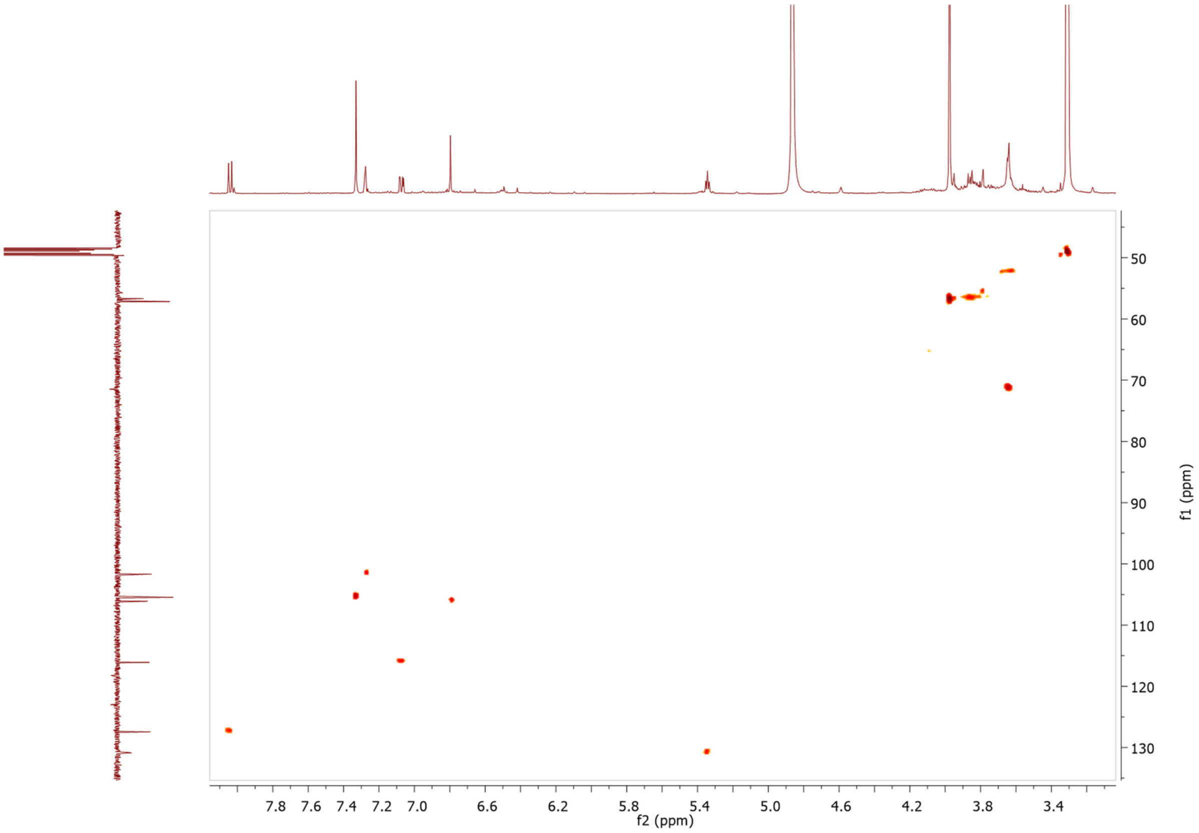

Figure S23. HSQC spectrum of compound **4**

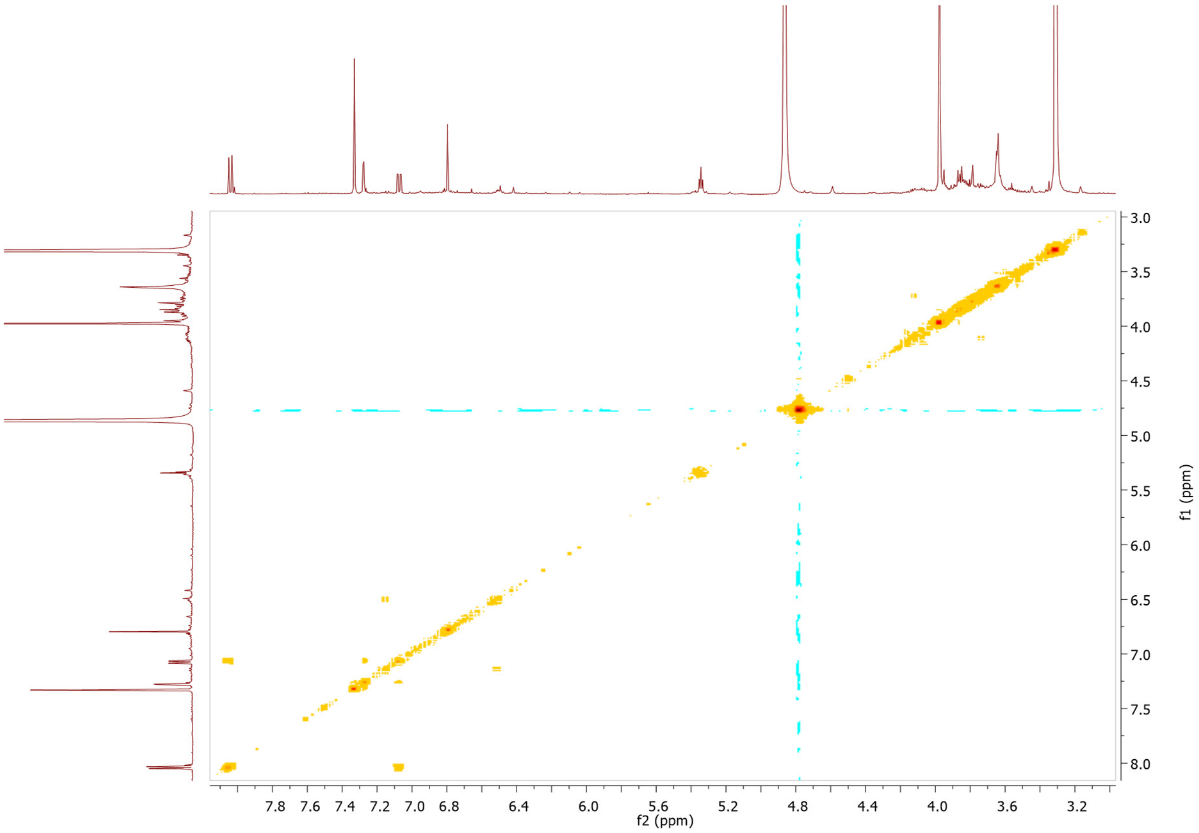

Figure S24. ^{1}H-^{1}H COSY spectrum of compound **4**

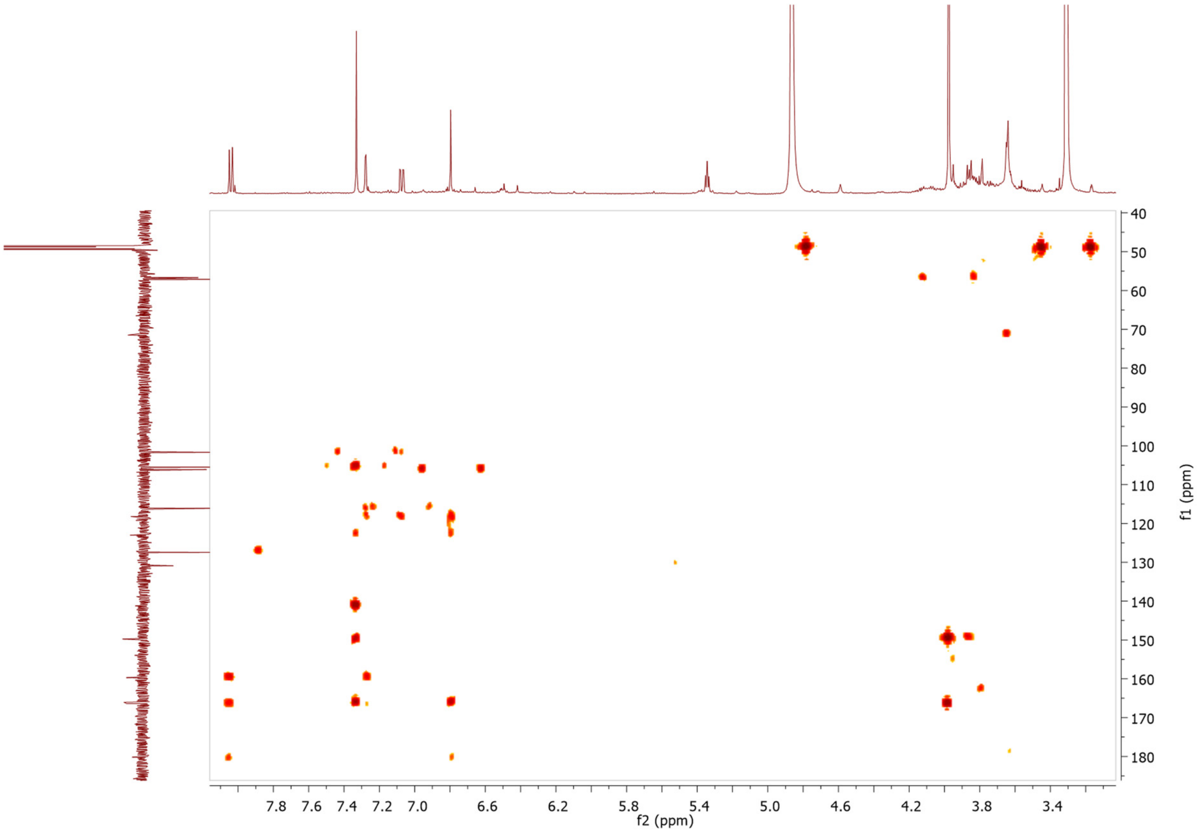

Figure S25. HMBC spectrum of compound **4**

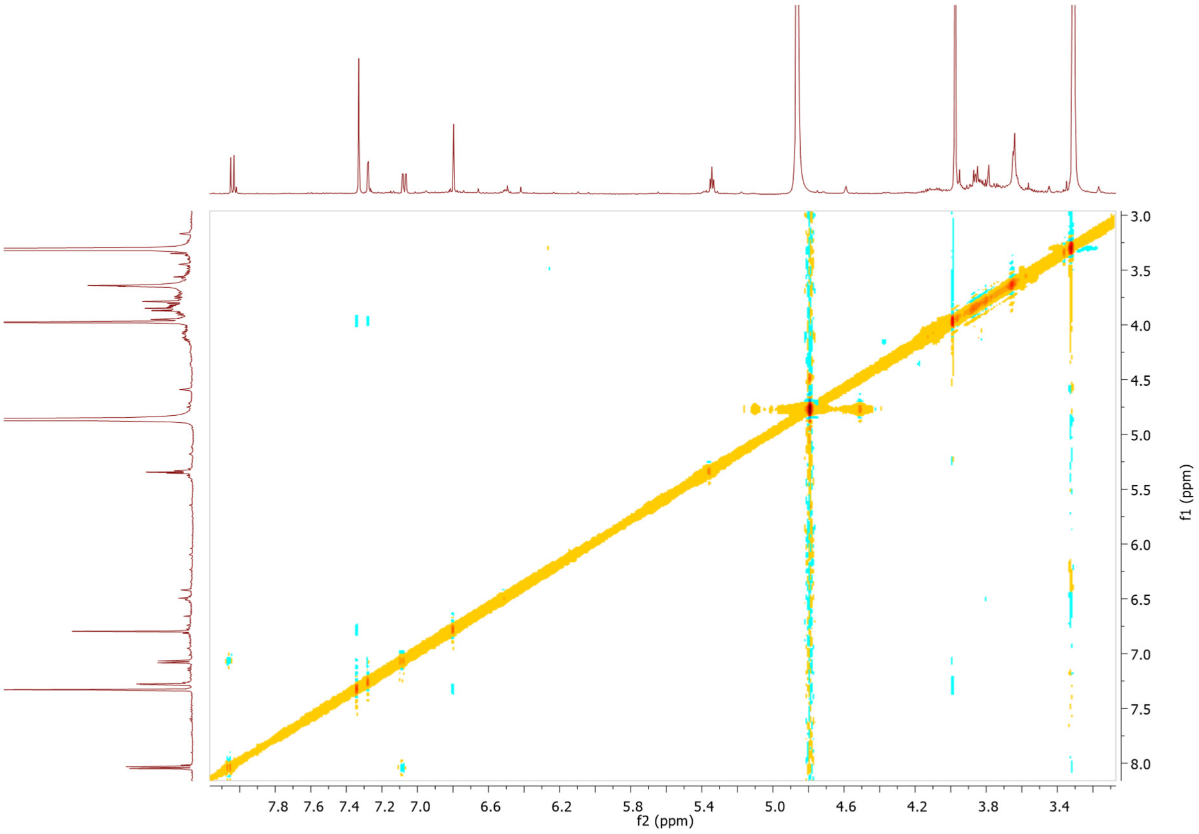

Figure S26. NOESY spectrum of compound **4**

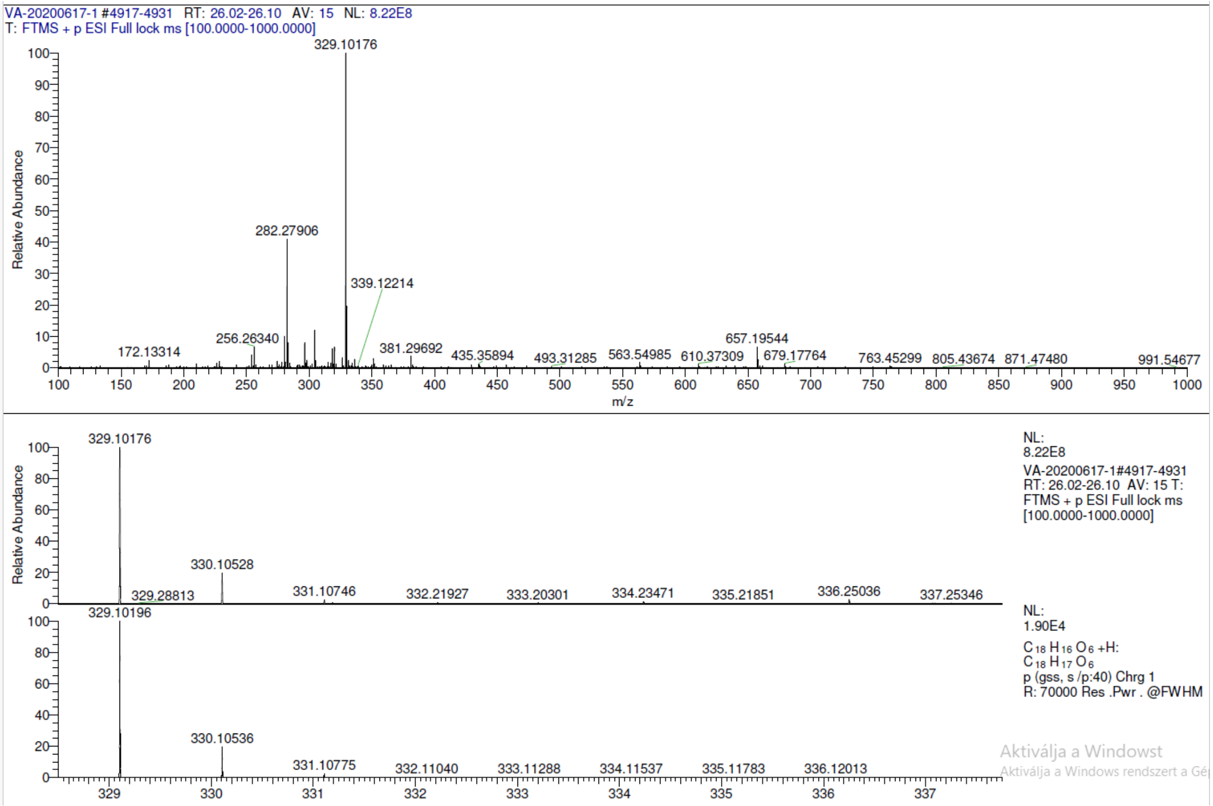

Figure S27. HRESIMS spectrum of compound **4**

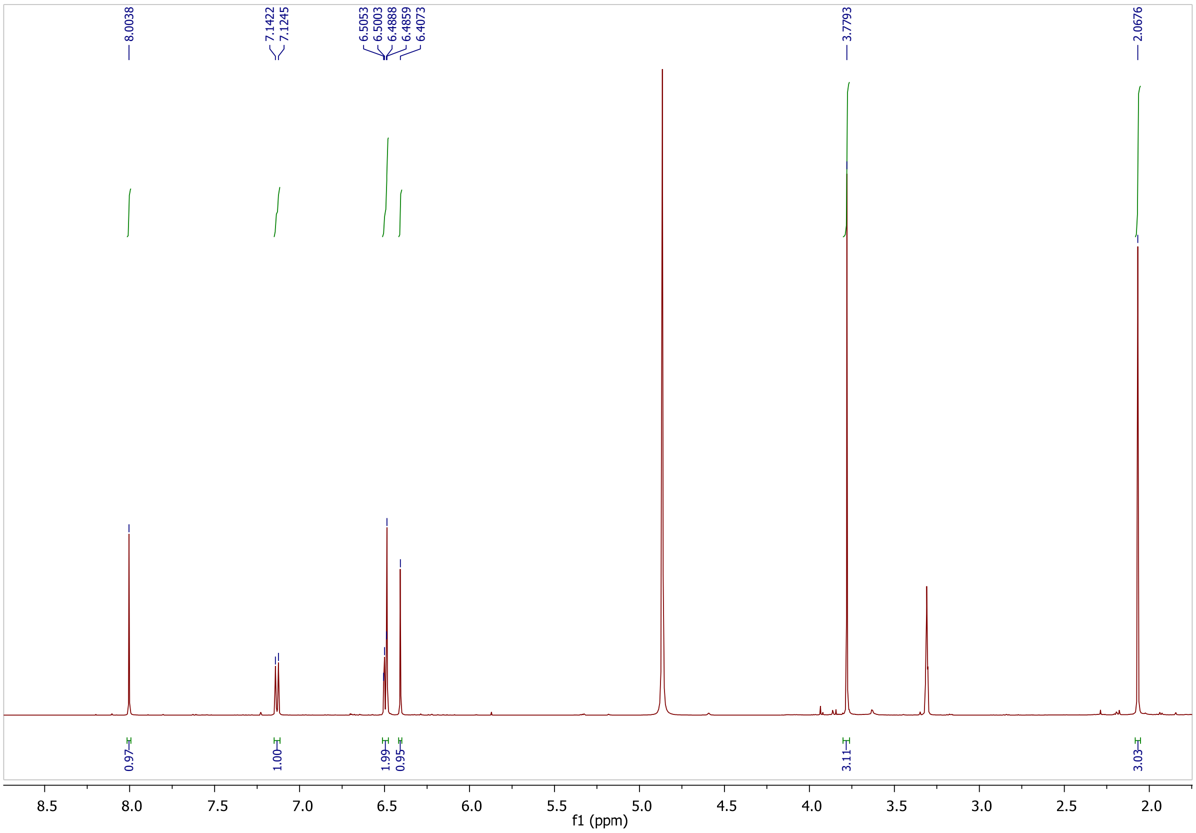

Figure S28. ^{1}H-NMR spectrum of compound **5** (500 MHz, CD_3OD)

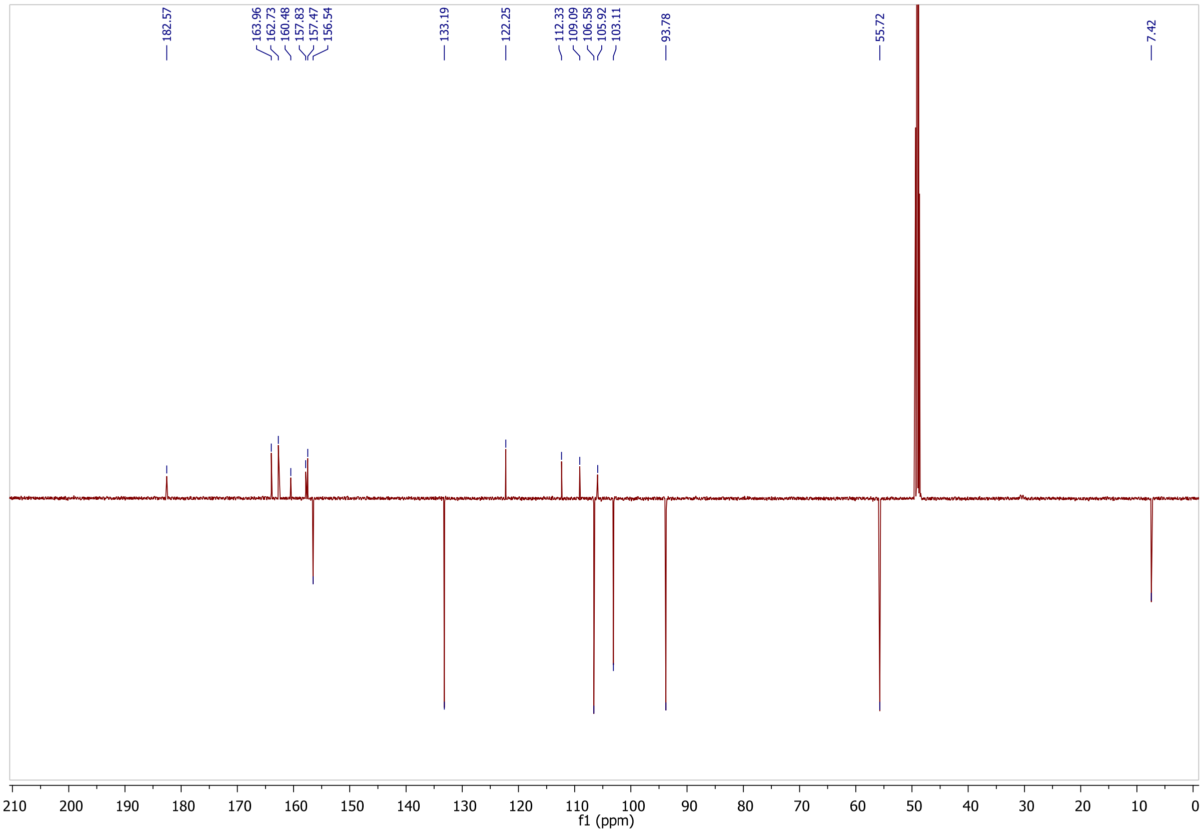

Figure S29. JMOD spectrum of compound **5** (125 MHz, CD_3OD)

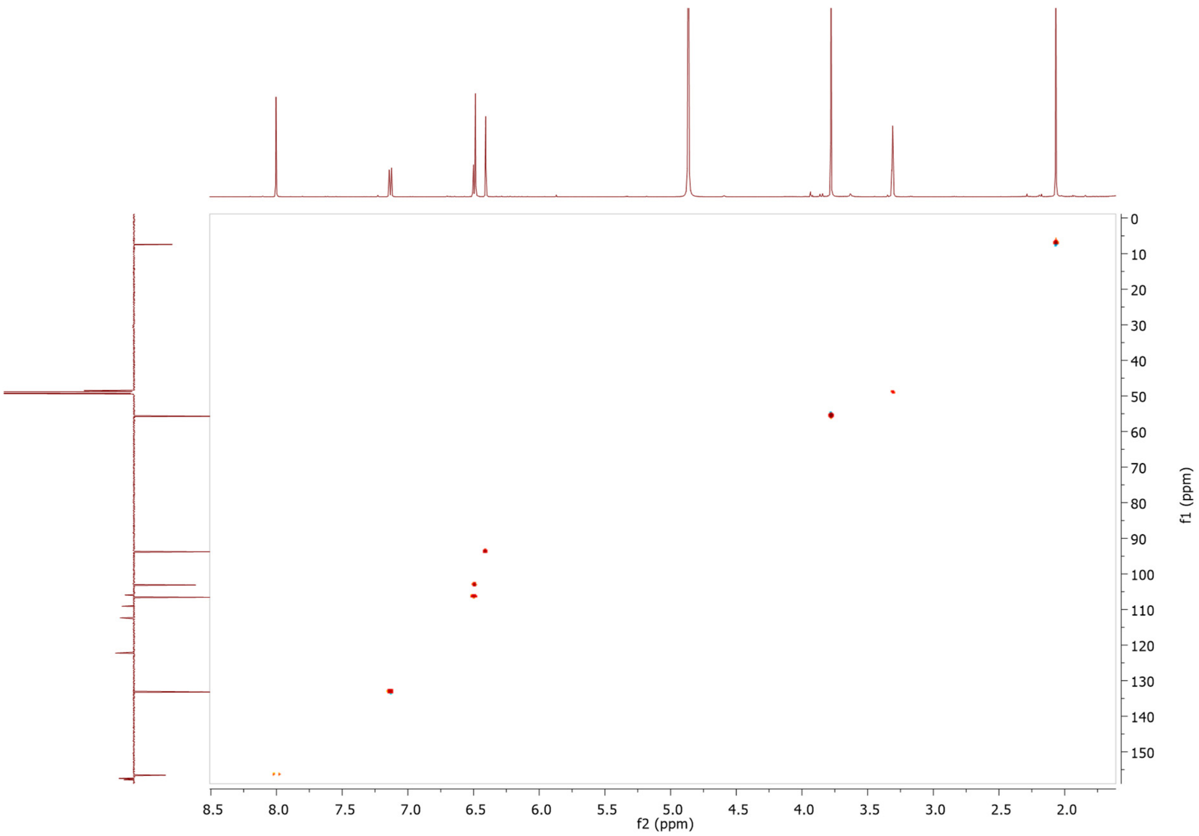

Figure S30. HSQC spectrum of compound **5**

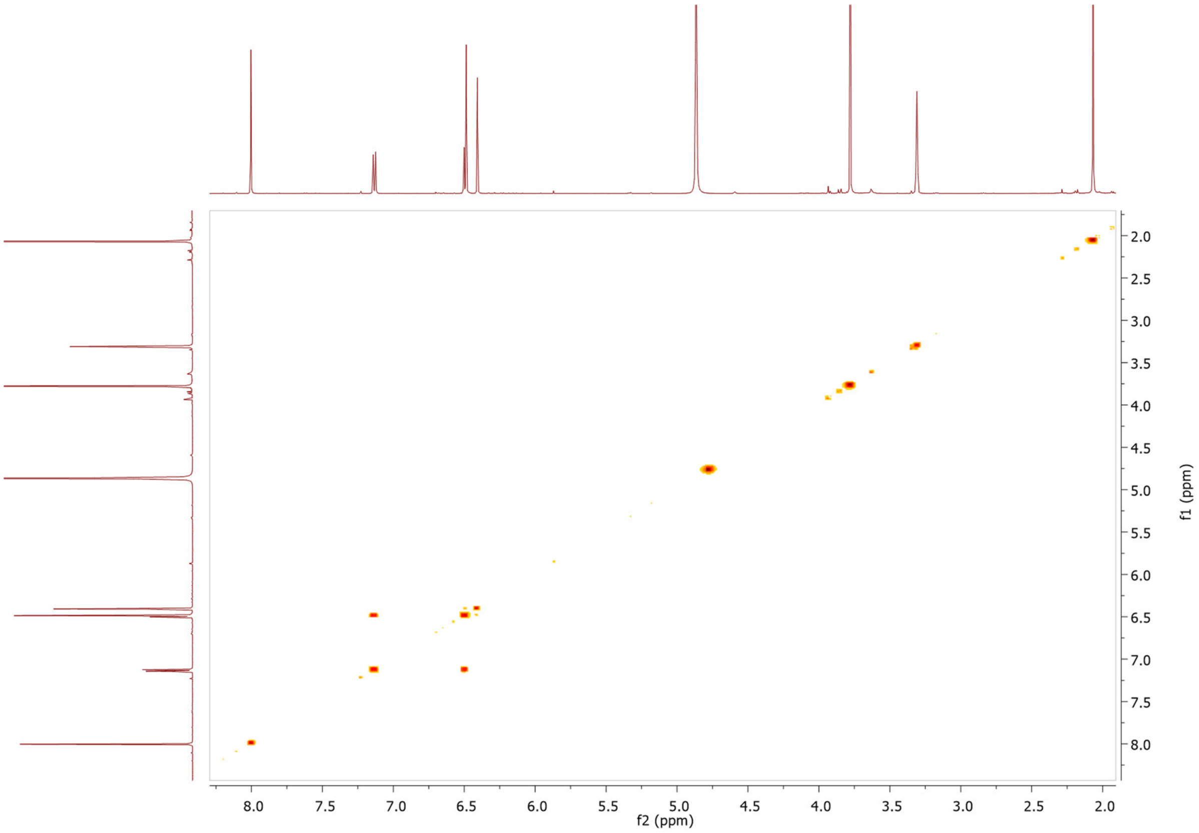

Figure S31. ^{1}H-^{1}H COSY spectrum of compound **5**

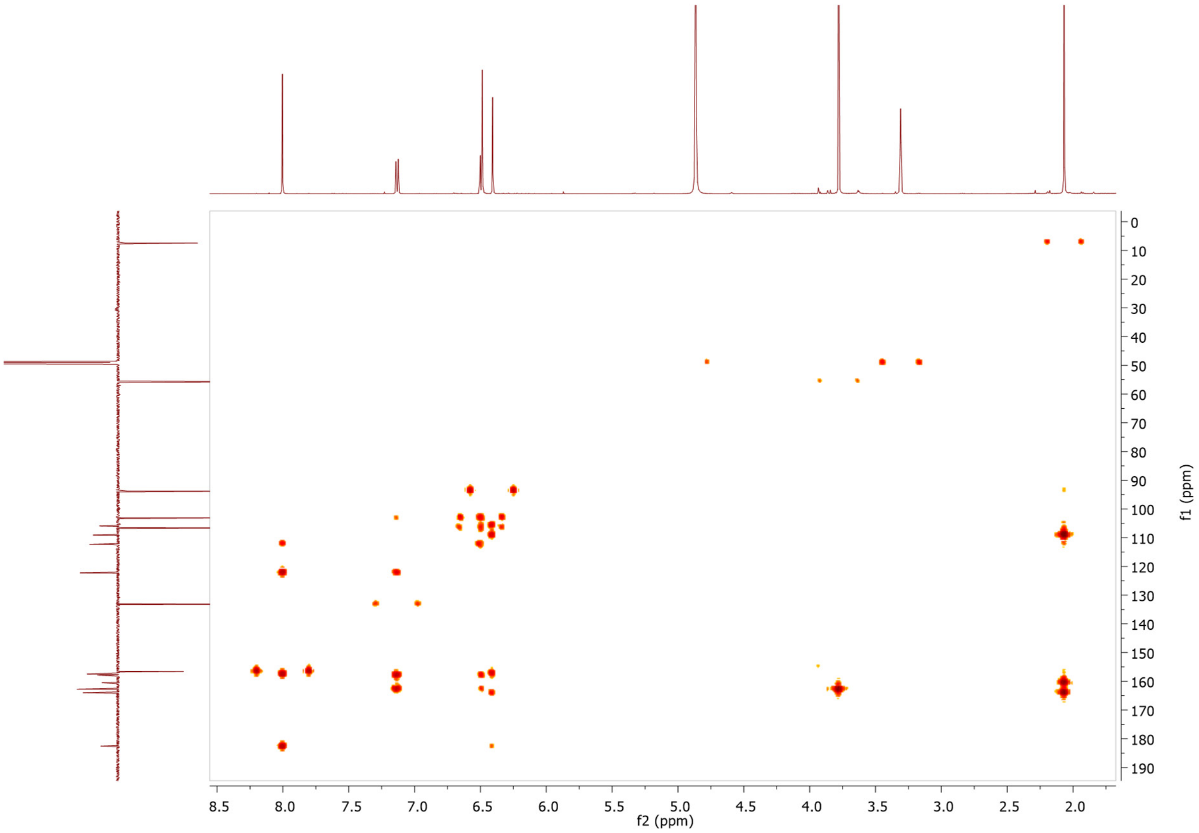

Figure S32. HMBC spectrum of compound **5**

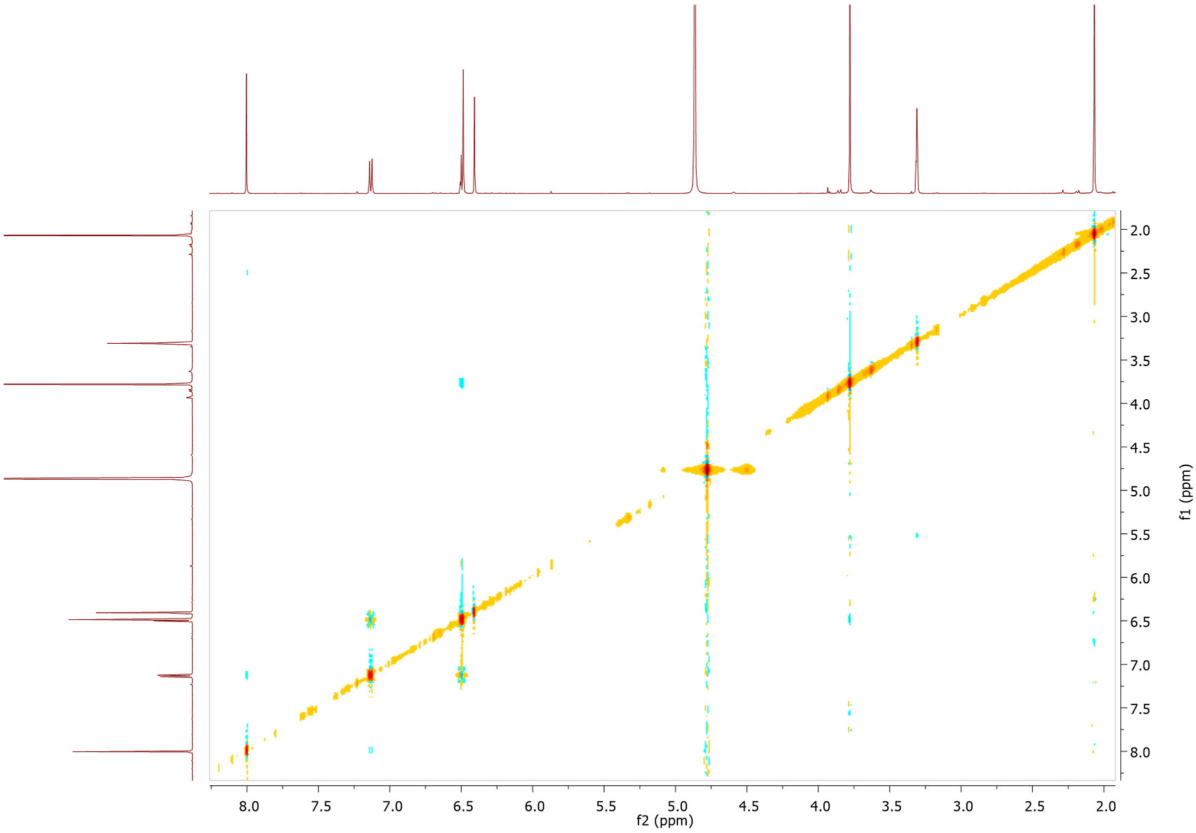

Figure S33. NOESY spectrum of compound **5**

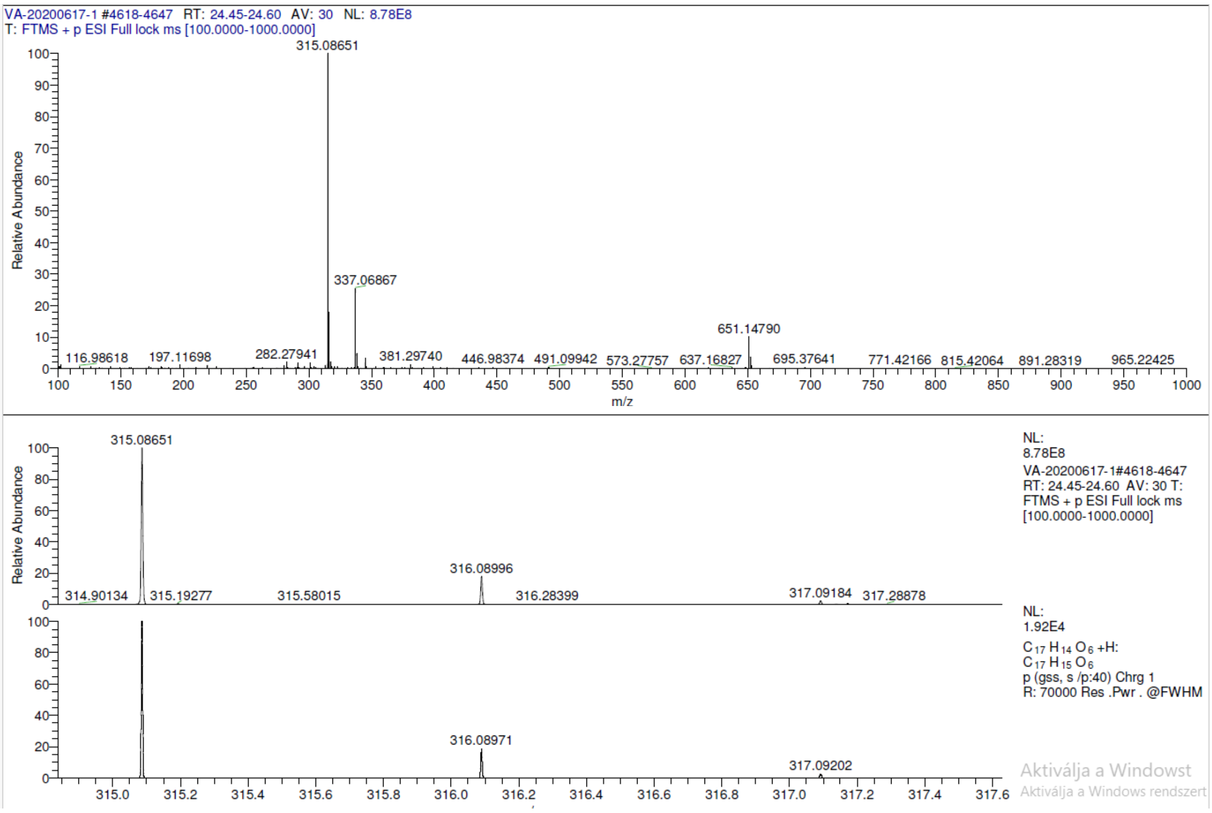

Figure S34. HRESIMS spectrum of compound **5**

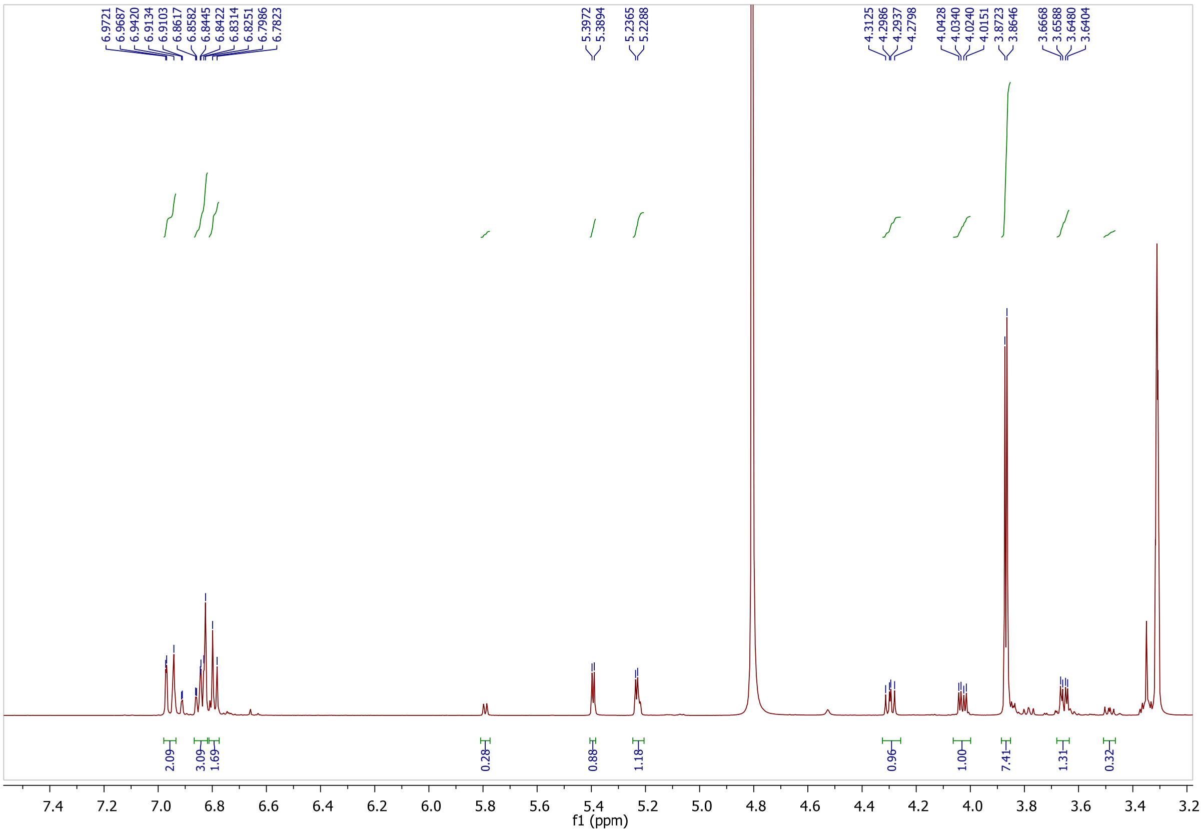

Figure S35. ^{1}H-NMR spectrum of compound **6** (500 MHz, CD_3OD)

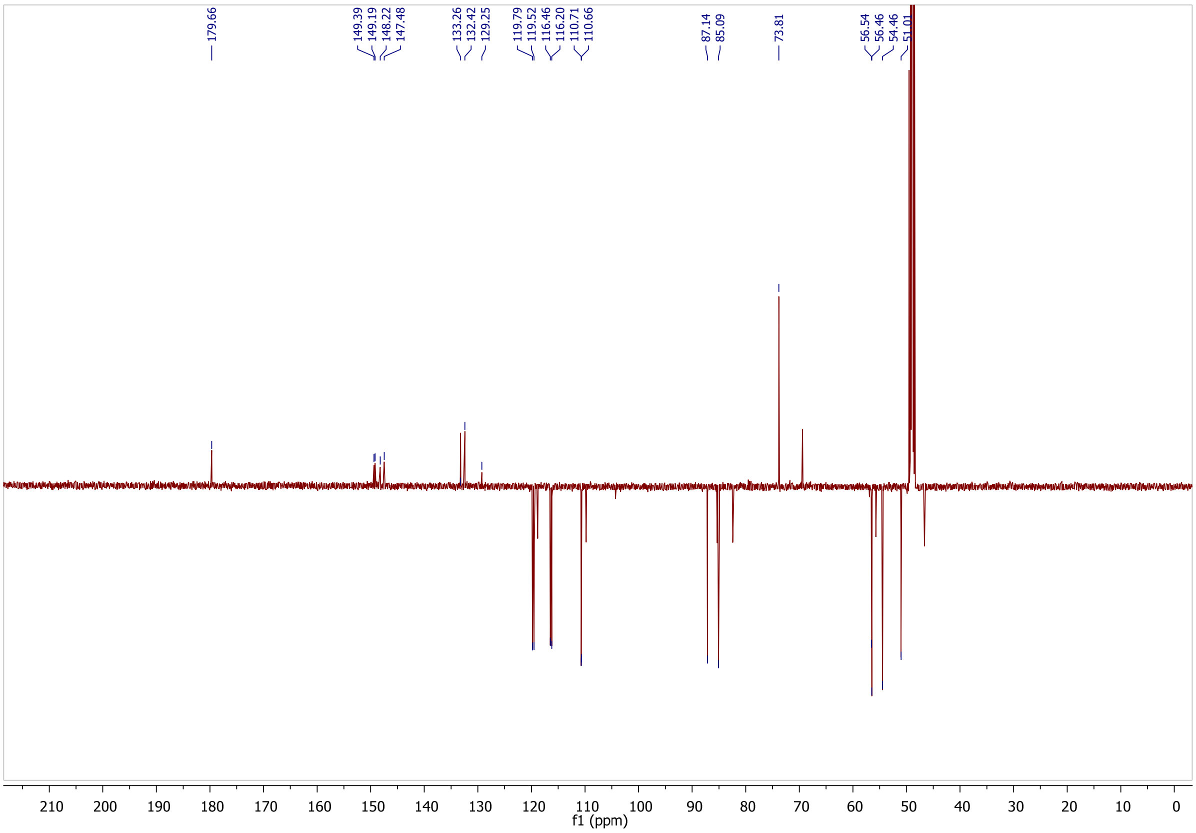

Figure S36. JMOD spectrum of compound **6** (125 MHz, CD_3OD)

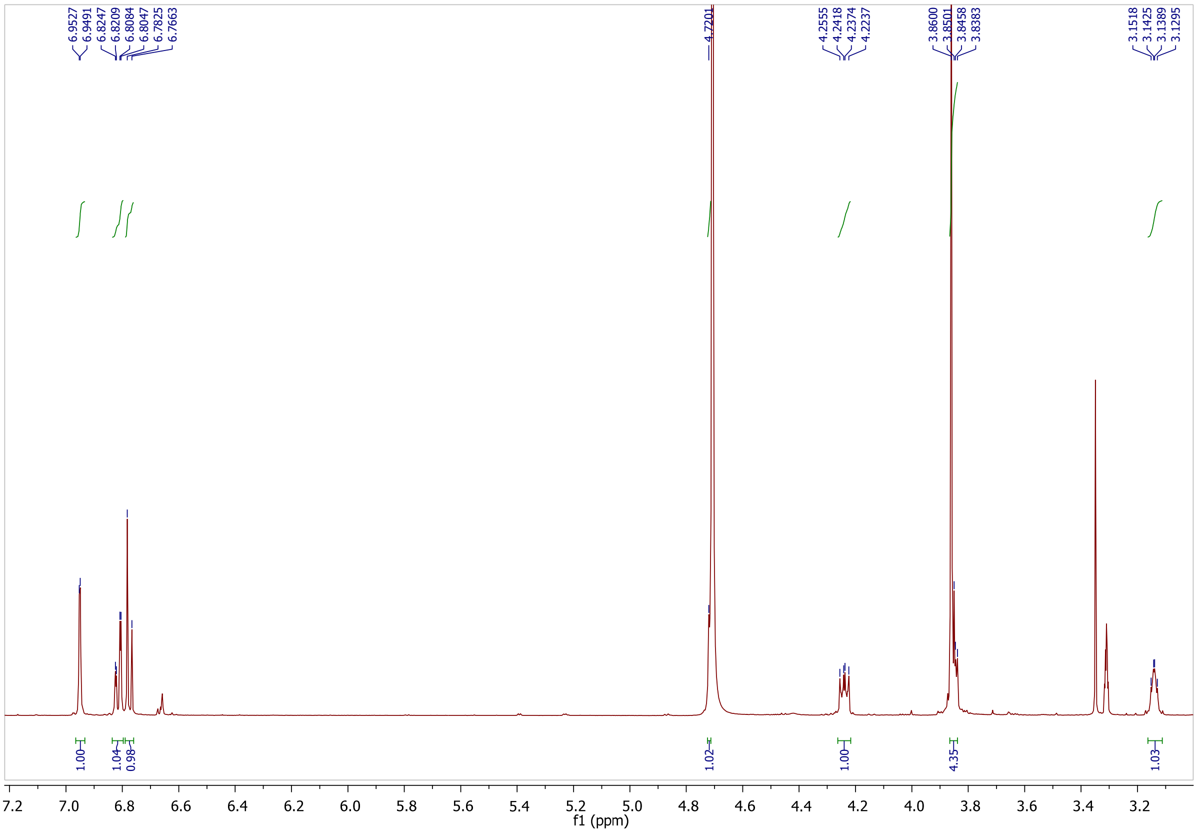

Figure S37. ^{1}H-NMR spectrum of compound **7** (500 MHz, CD_3OD)

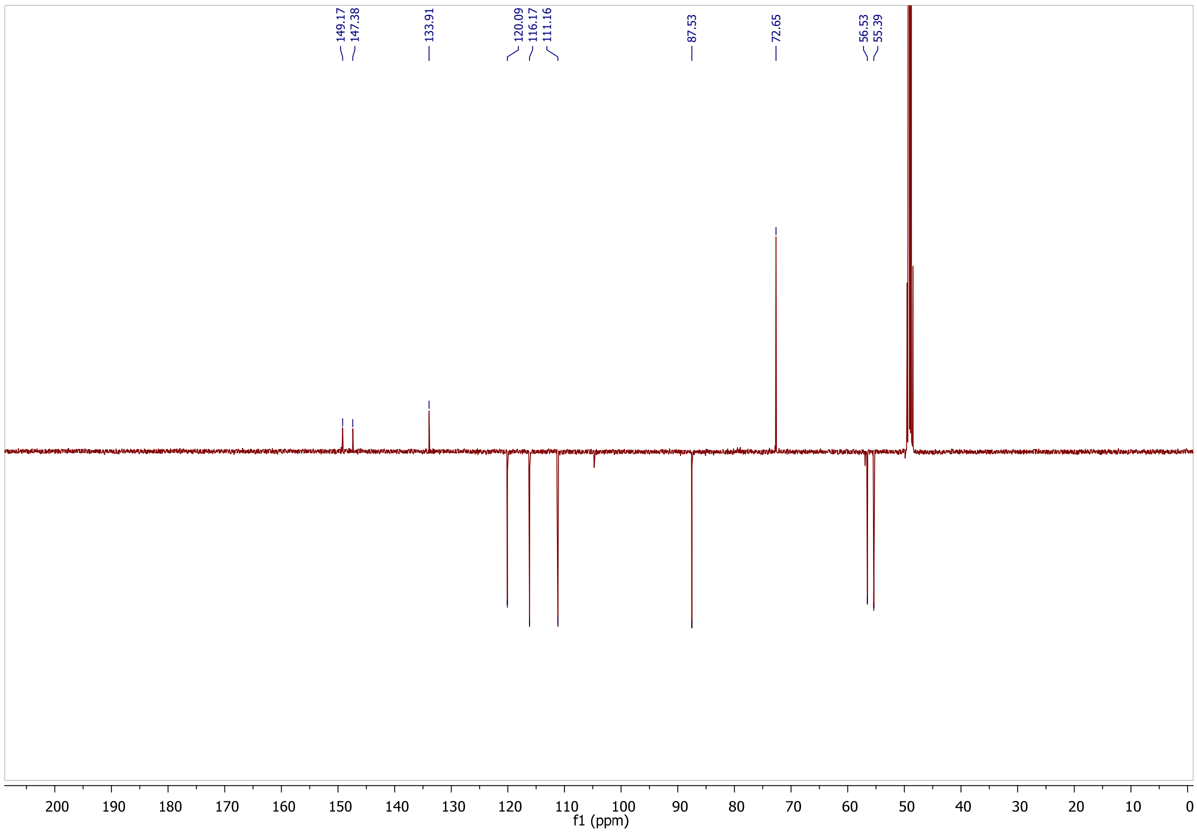

Figure S38. JMOD spectrum of compound **7** (125 MHz, CD_3OD)

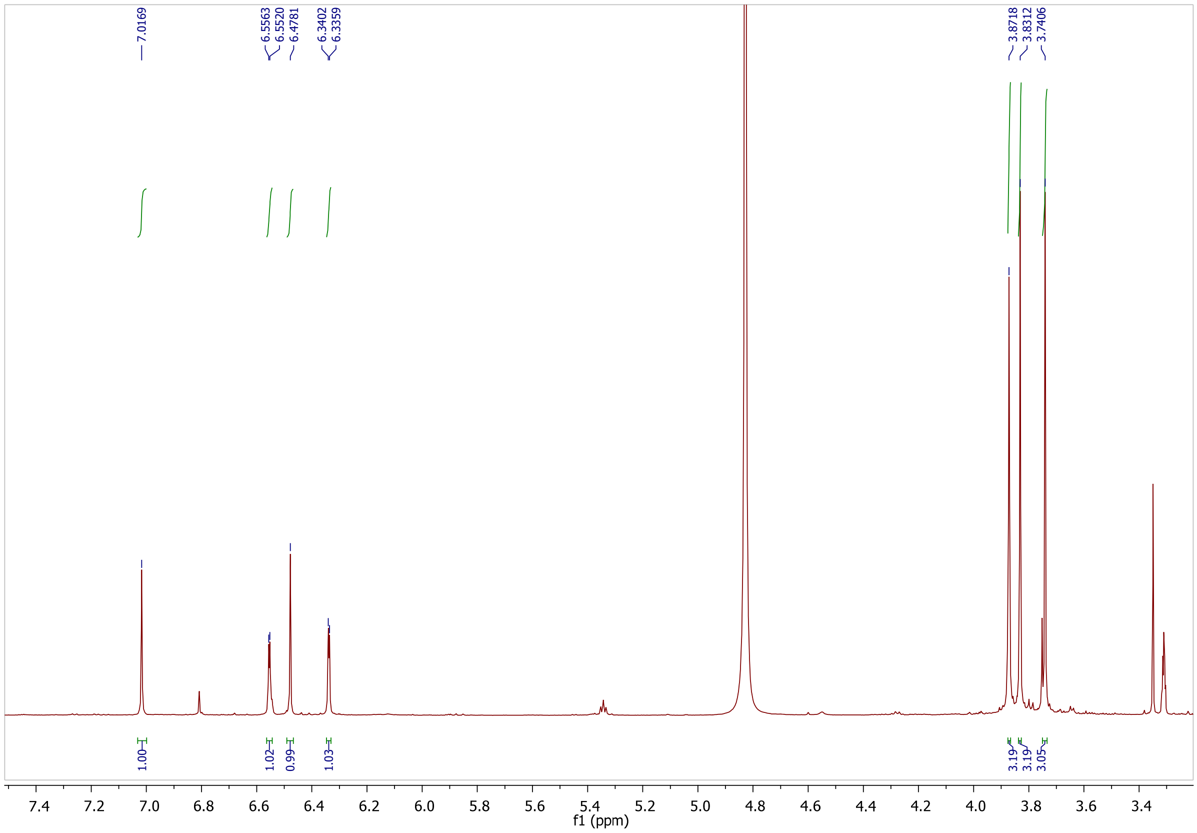

Figure S39. ^{1}H-NMR spectrum of compound **8** (500 MHz, CD_3OD)

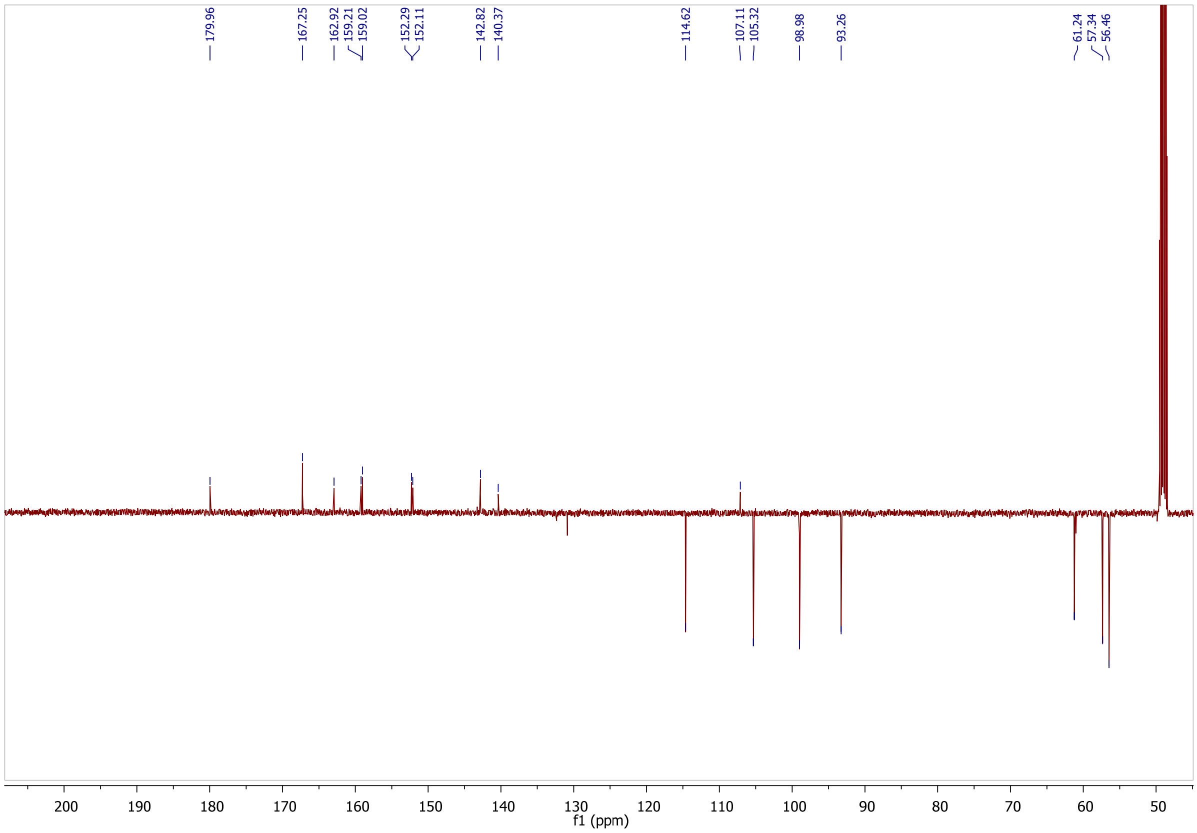

Figure S40. JMOD spectrum of compound **8** (125 MHz, CD_3OD)

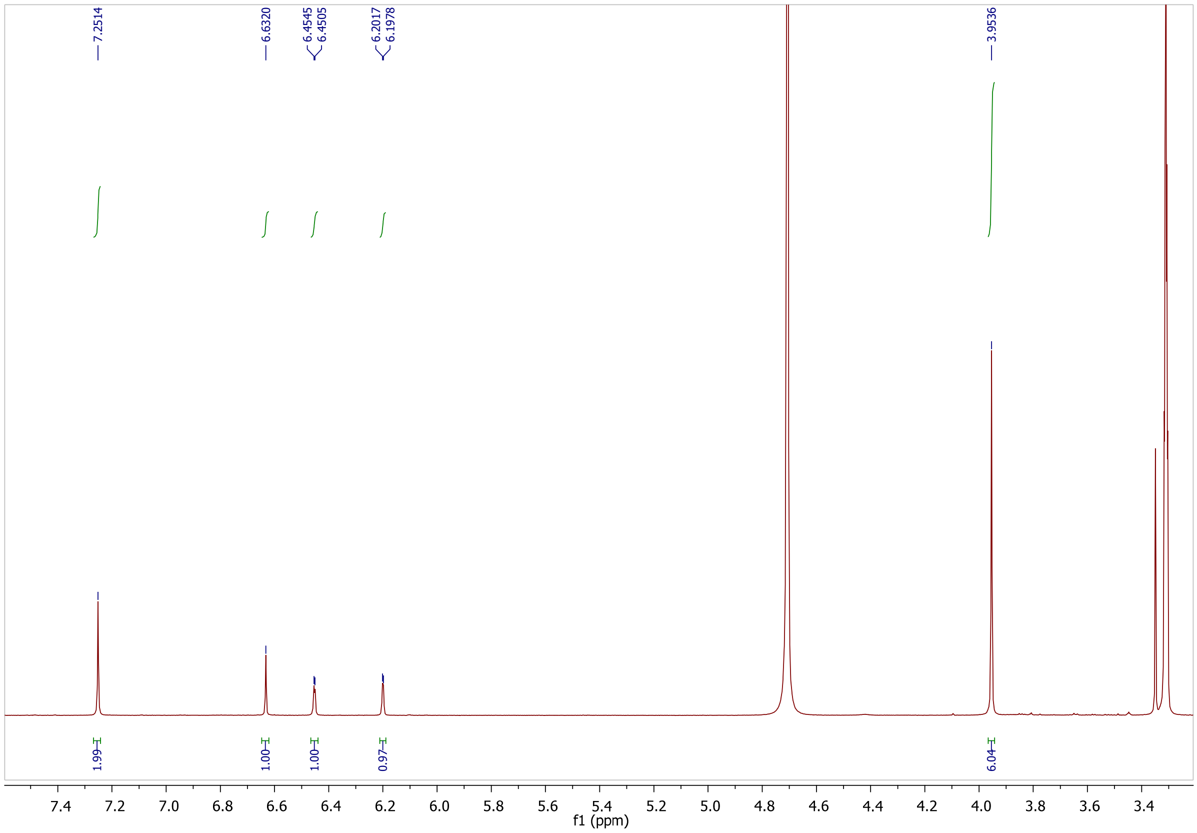

Figure S41. ^{1}H-NMR spectrum of compound **9** (500 MHz, CD_3OD)

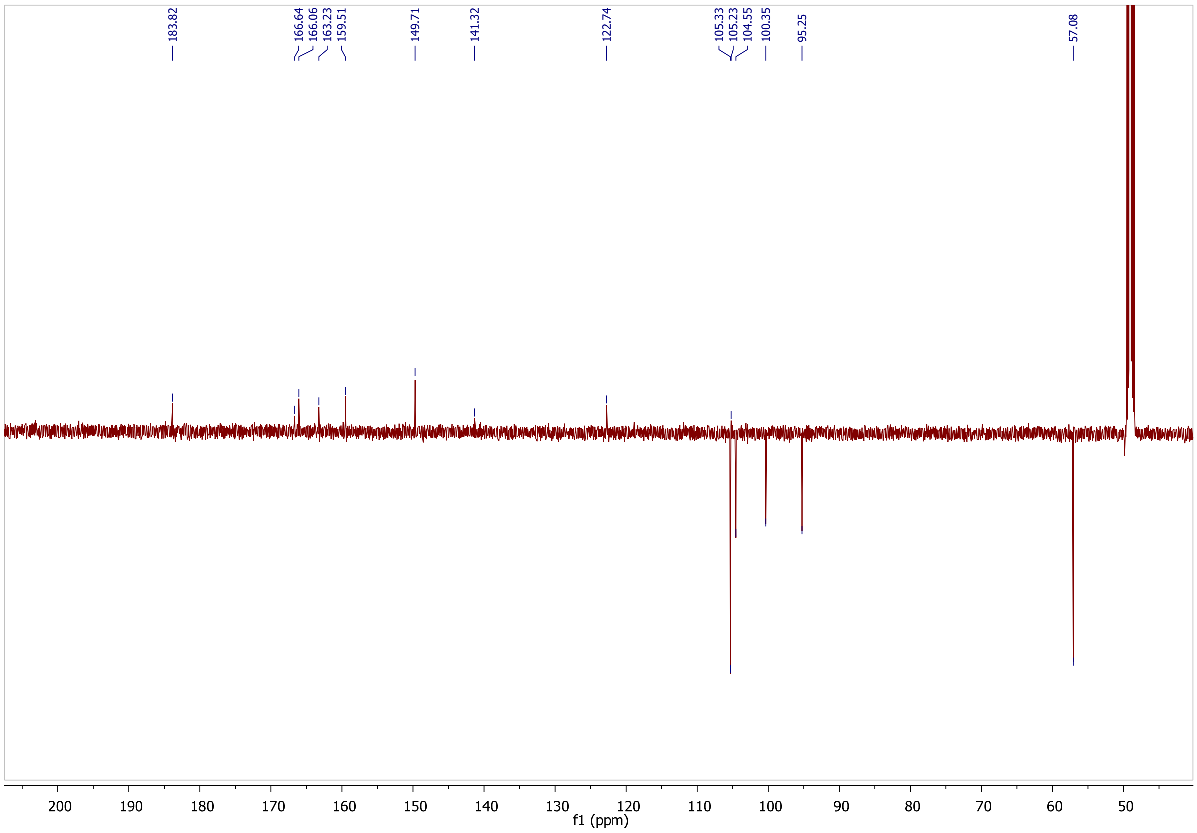

Figure S42. JMOD spectrum of compound **9** (125 MHz, CD_3OD)

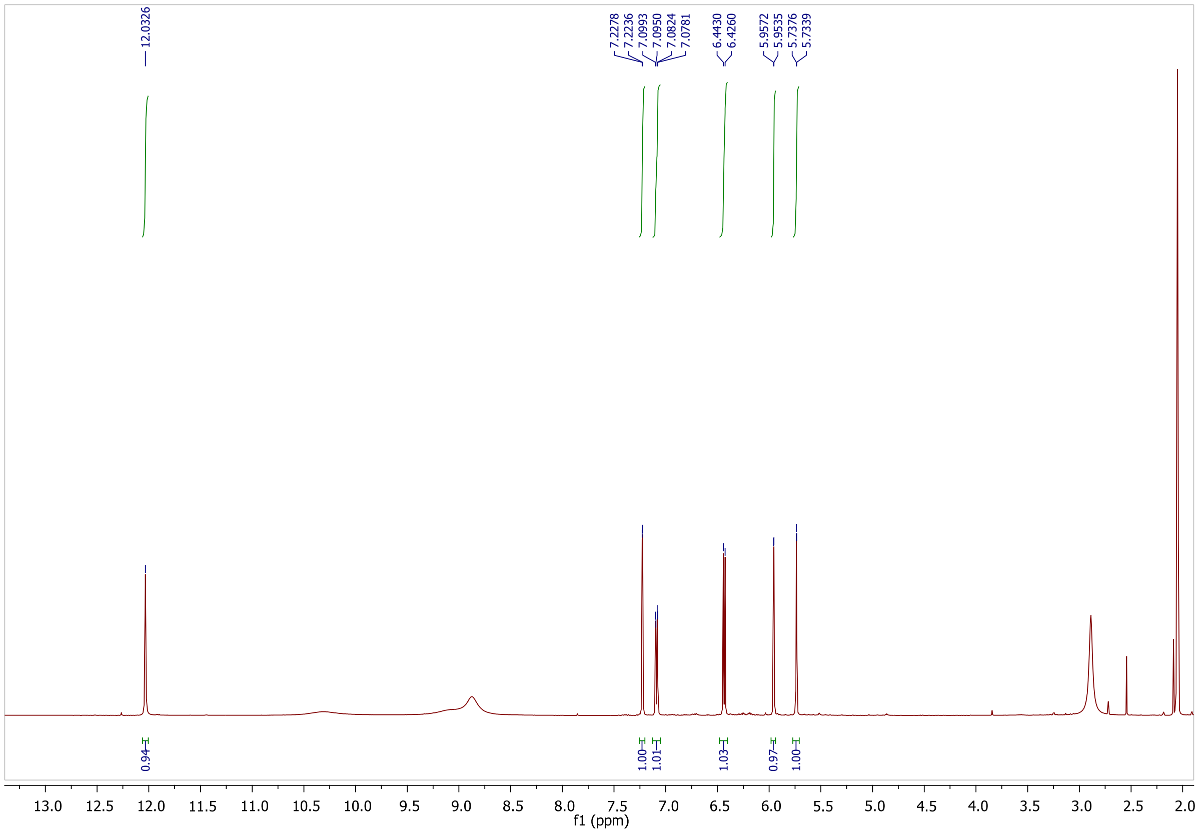

Figure S43. ^{1}H-NMR spectrum of compound **10** (500 MHz, DMSO-d_6)

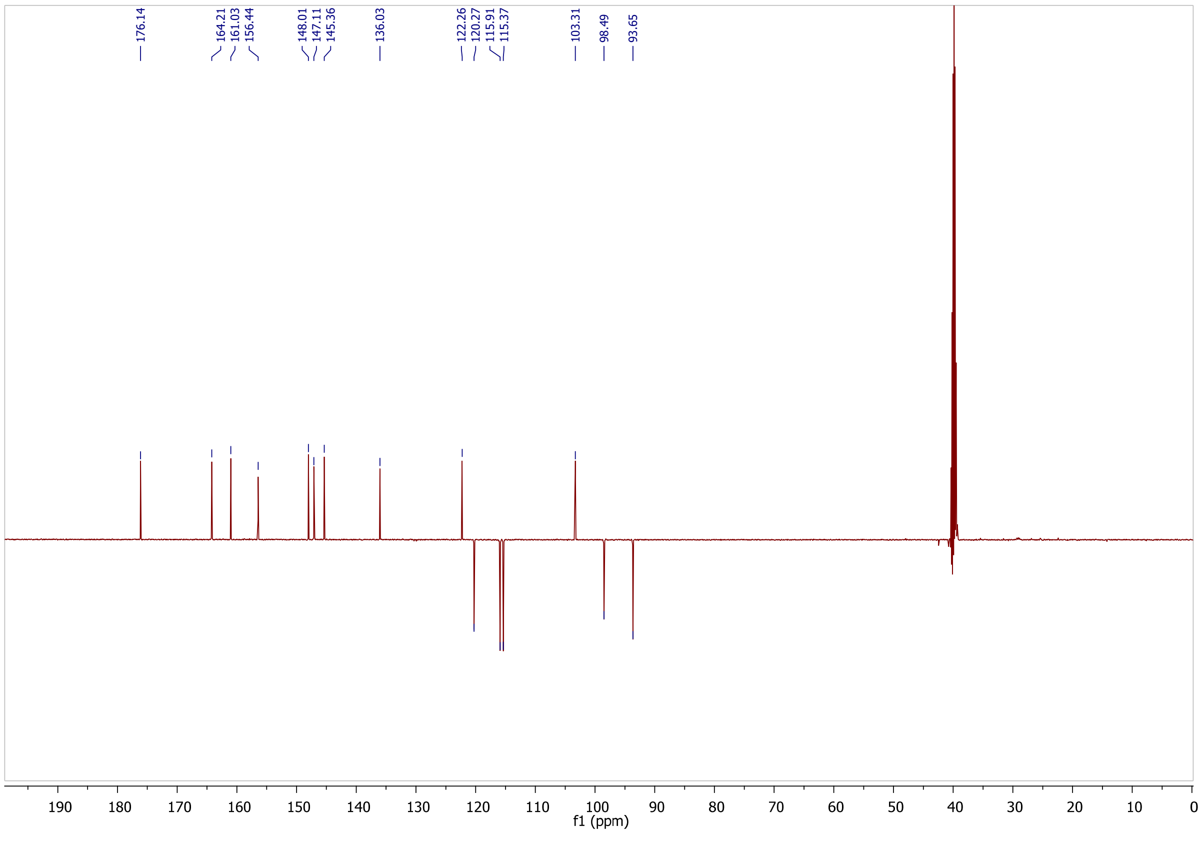

Figure S44. JMOD spectrum of compound **10** (125 MHz, DMSO-d_6)

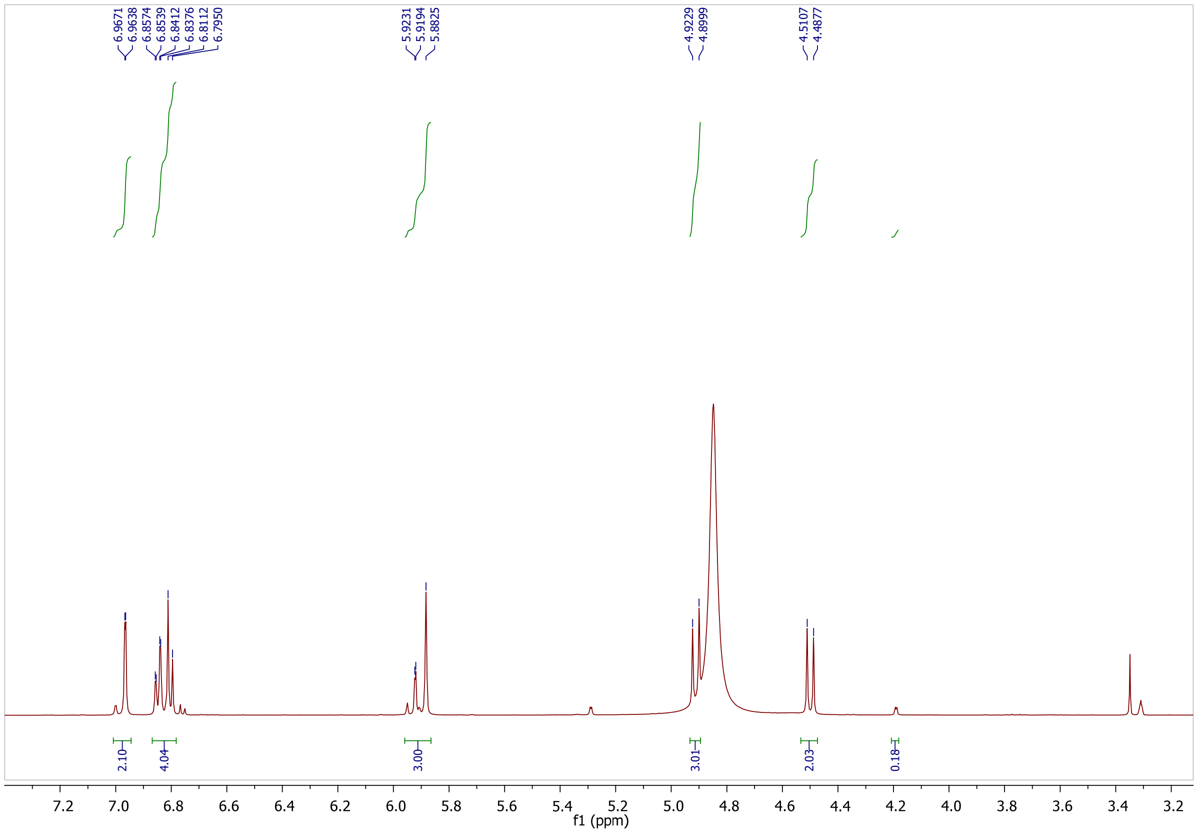

Figure S45. ^{1}H-NMR spectrum of compound **11** (500 MHz, CD_3OD)

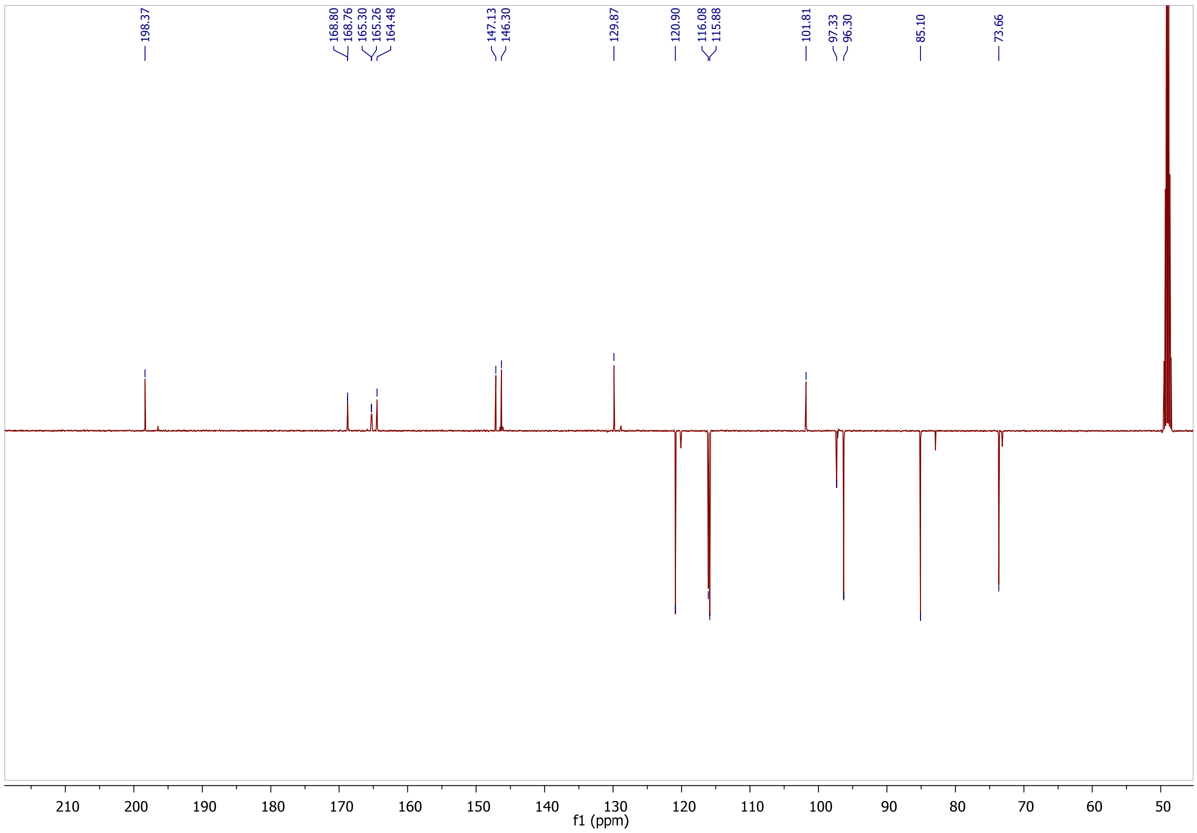

Figure S46. JMOD spectrum of compound **11** (125 MHz, CD_3OD)

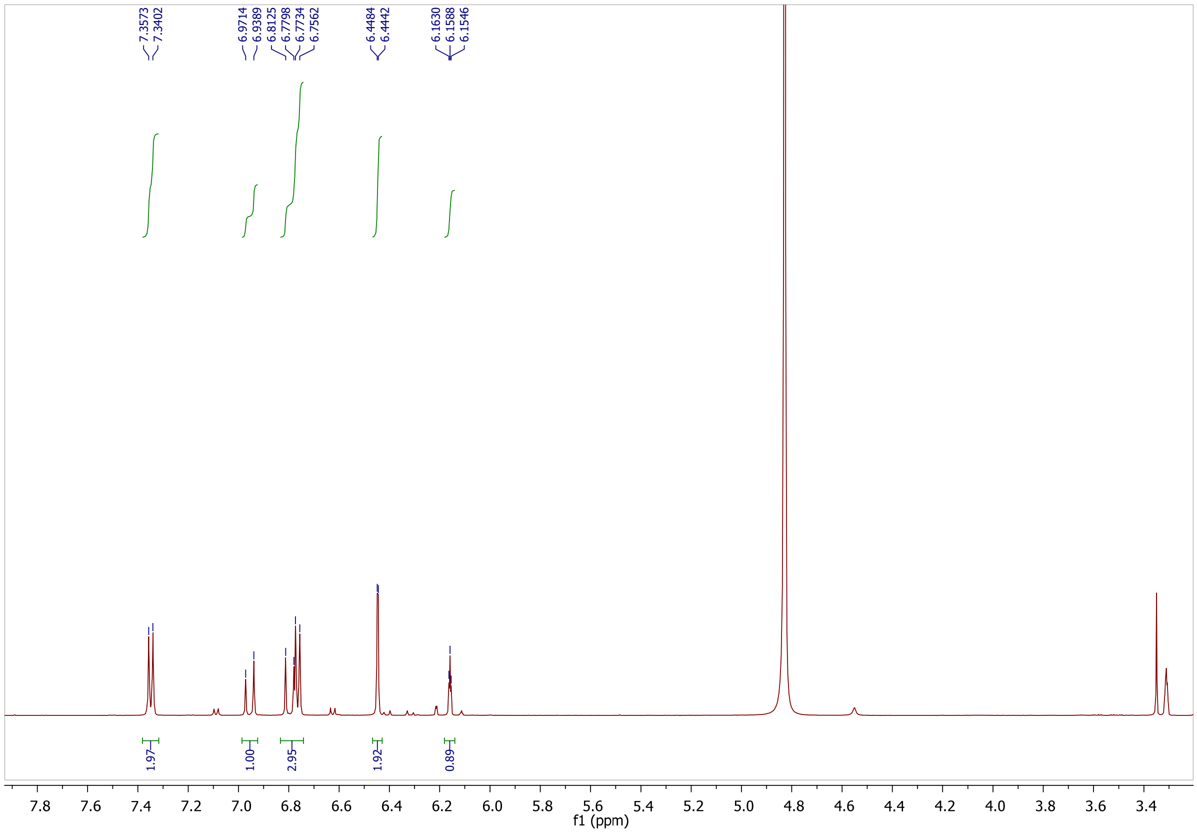

Figure S47. ^{1}H-NMR spectrum of compound **12** (500 MHz, CD_3OD)

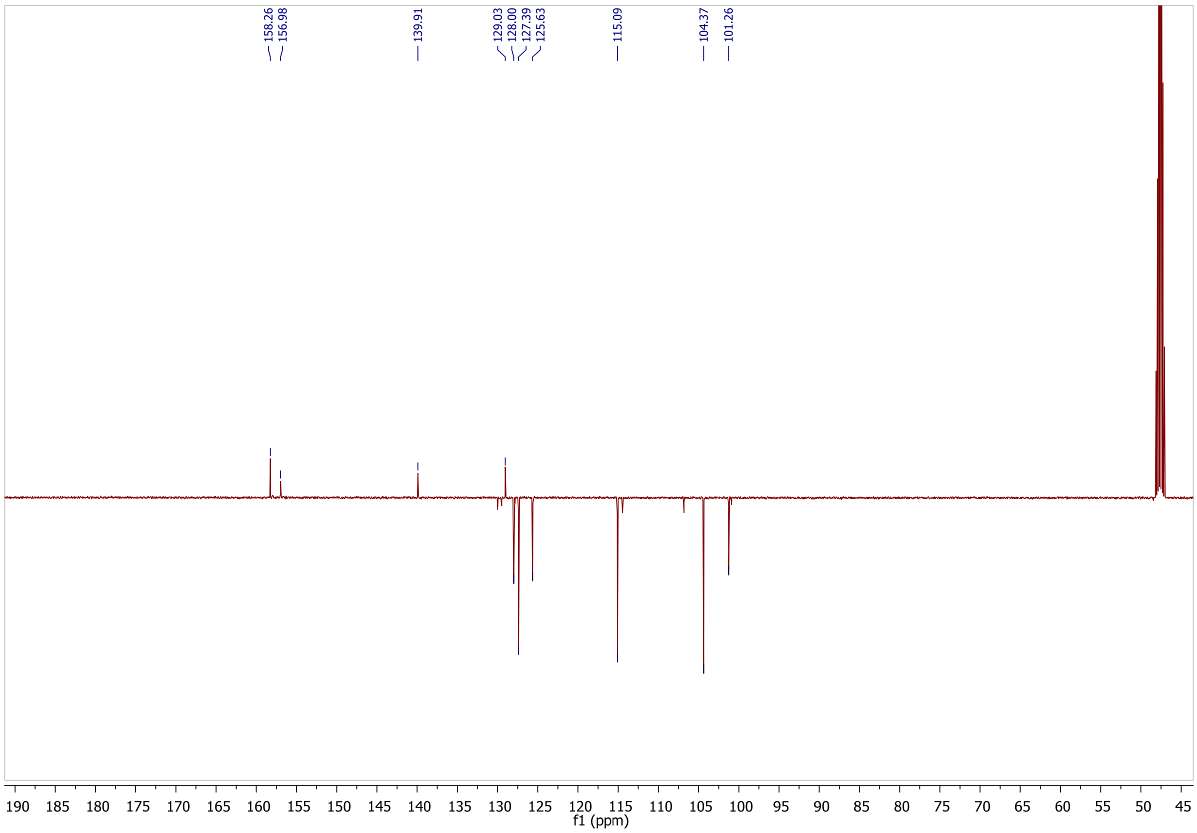

Figure S48. JMOD spectrum of compound **12** (125 MHz, CD_3OD)

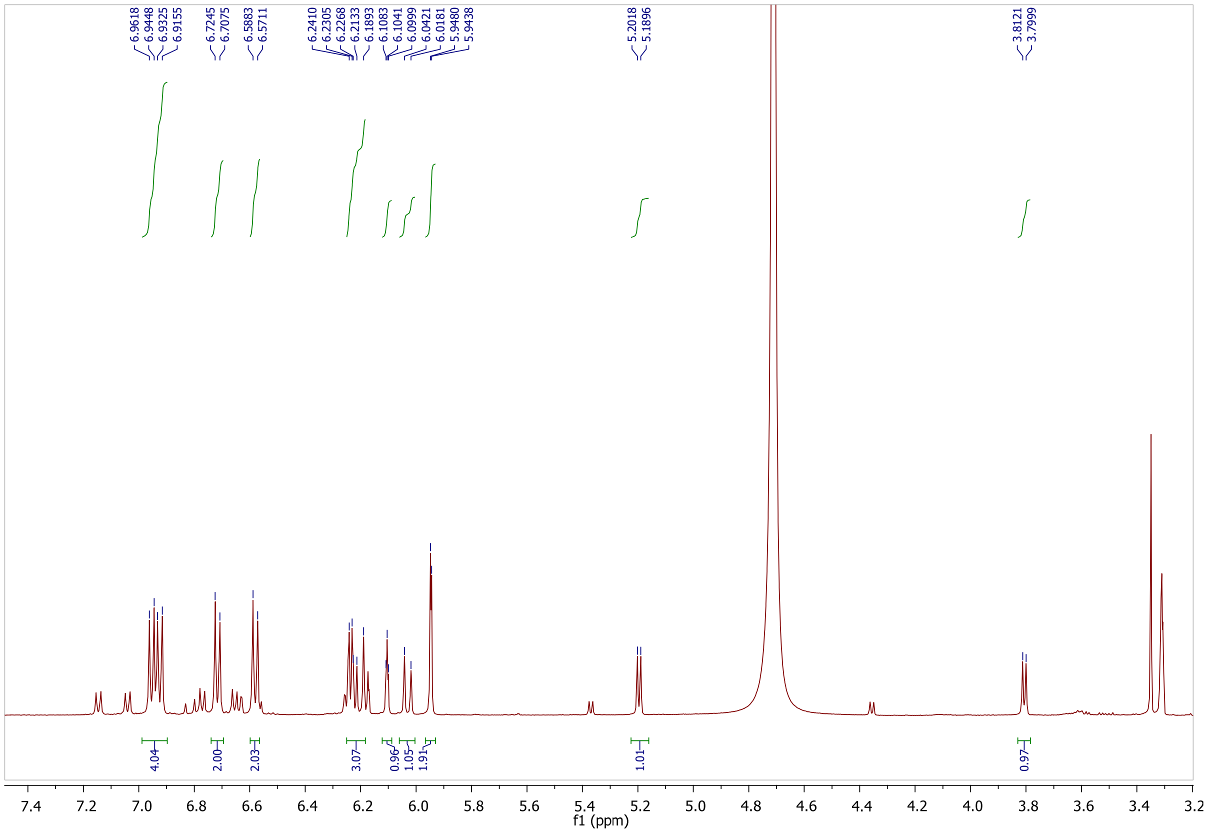

Figure S49. ^{1}H-NMR spectrum of compound **13** (500 MHz, CD_3OD)

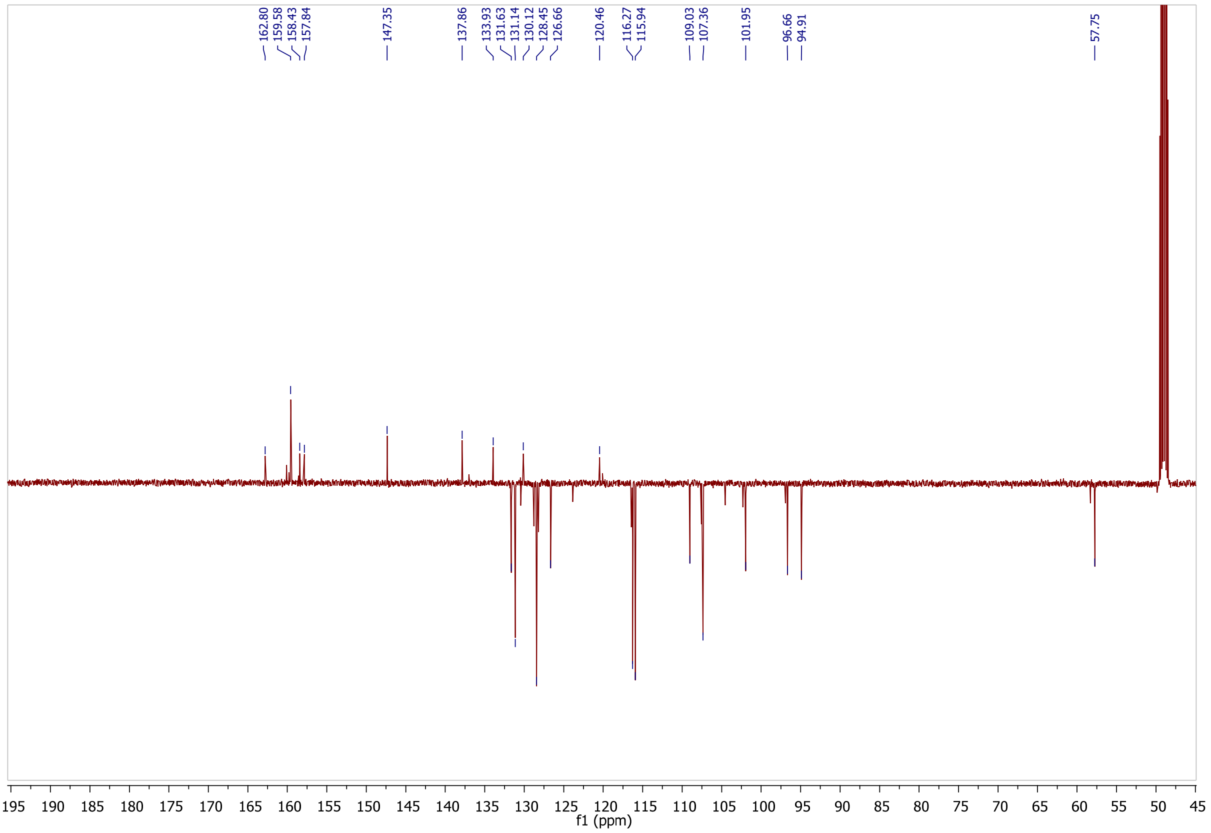

Figure S50. JMOD spectrum of compound **13** (125 MHz, CD_3OD)

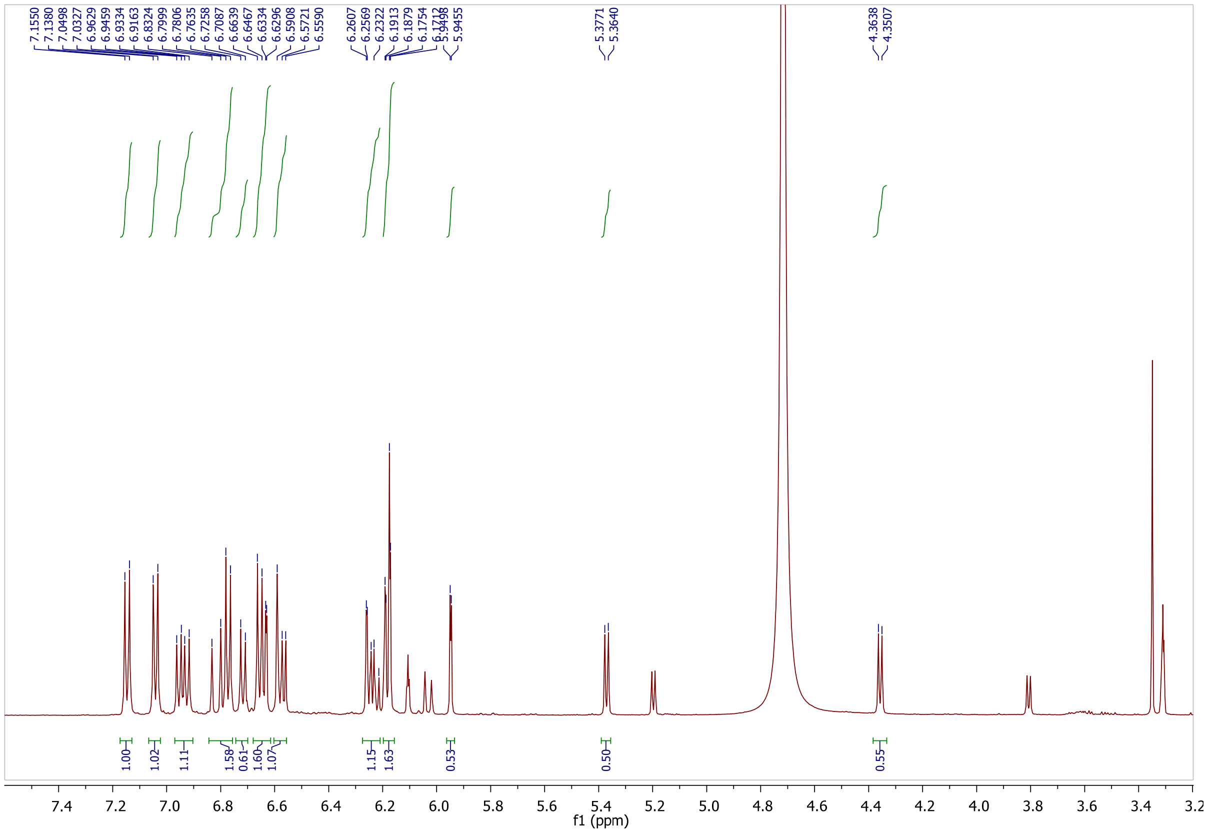

Figure S51. ^{1}H-NMR spectrum of compound **14** (500 MHz, CD_3OD)

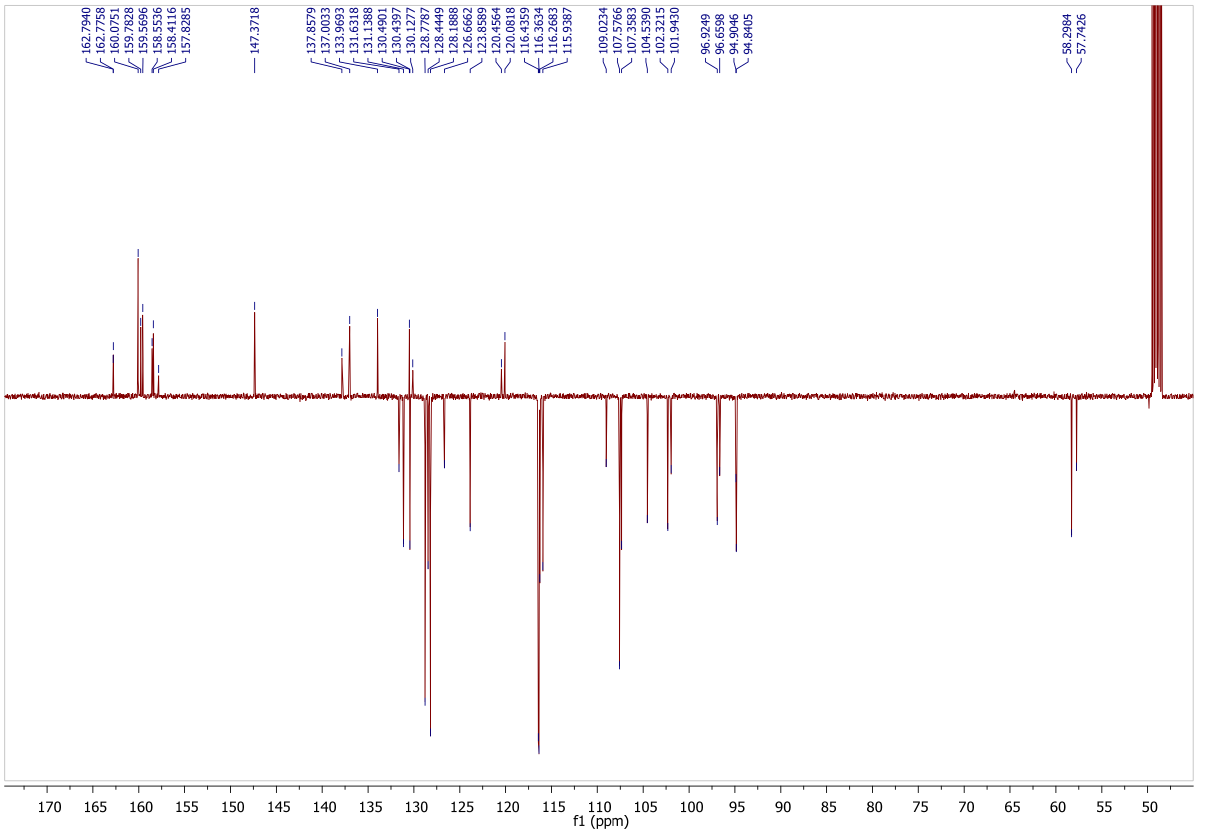

Figure S52. JMOD spectrum of compound **14** (125 MHz, CD_3OD)

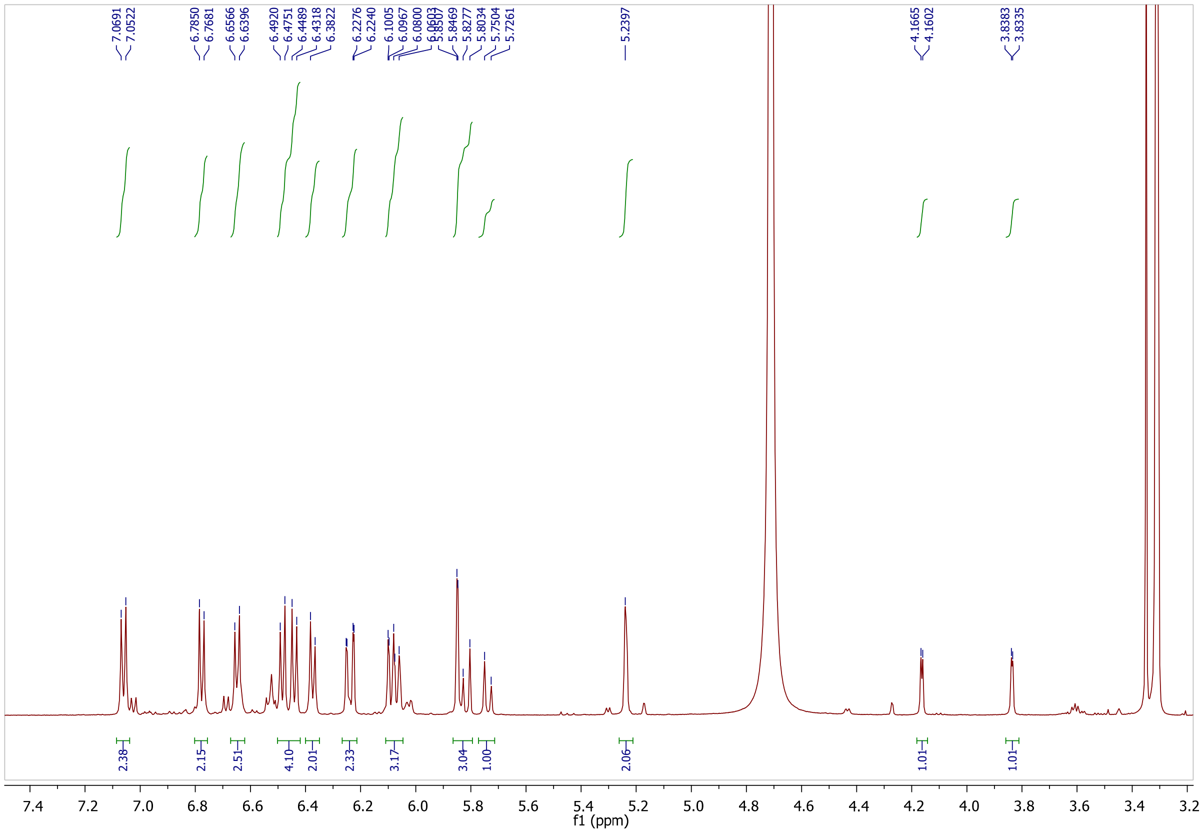

Figure S53. ^{1}H-NMR spectrum of compound **15** (500 MHz, CD_3OD)

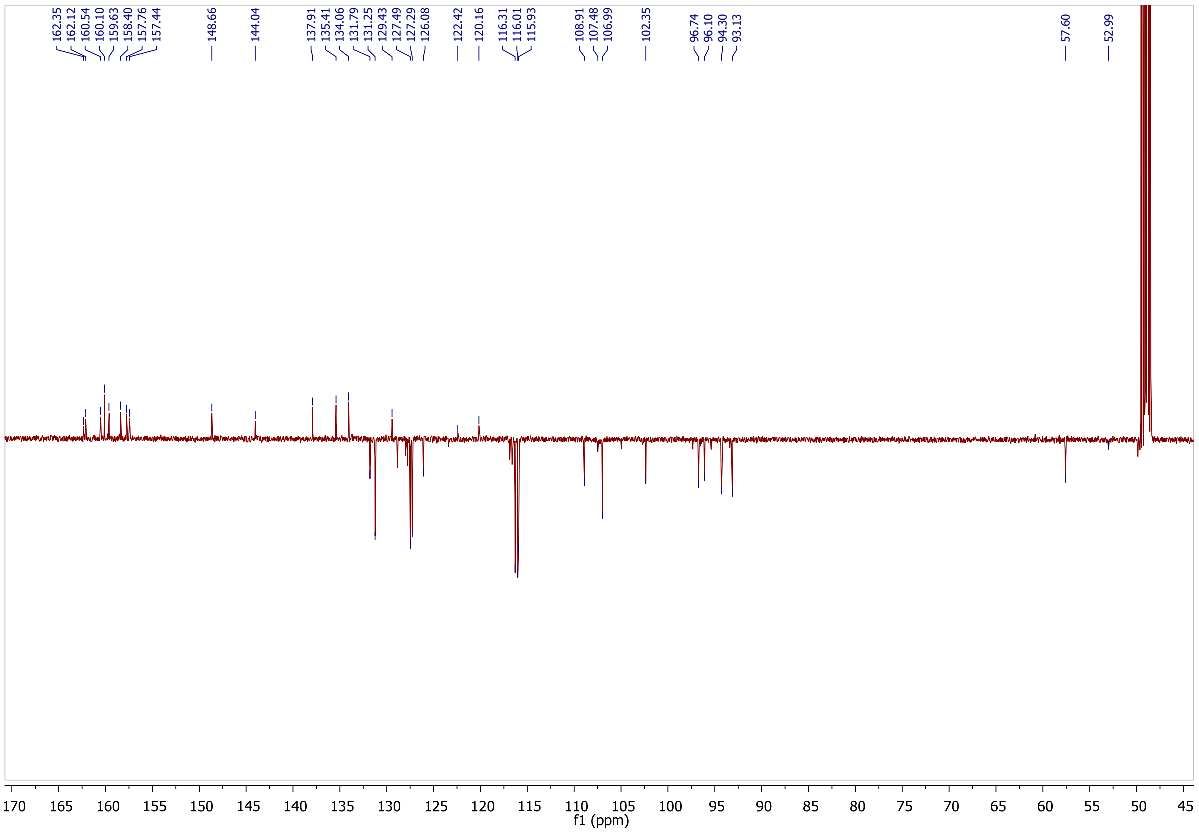

Figure S54. JMOD spectrum of compound **15** (125 MHz, CD_3OD)

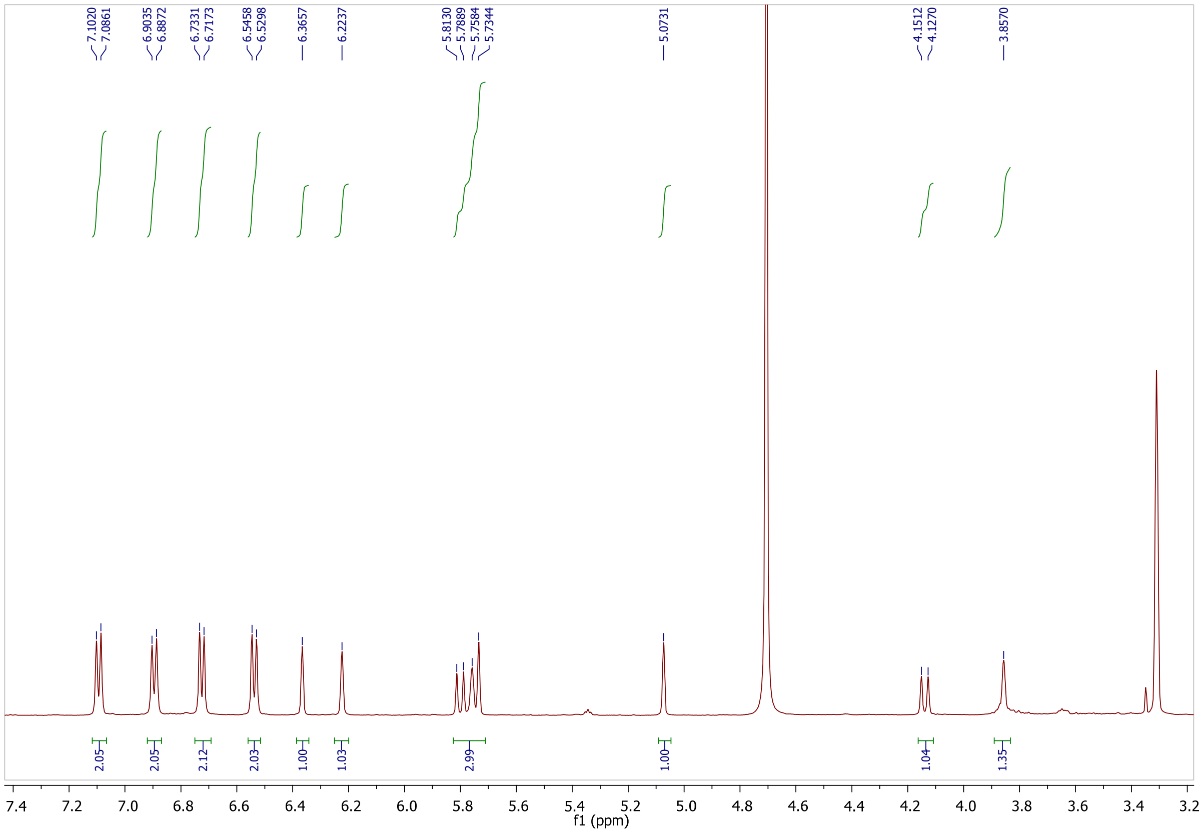

Figure S55. ^{1}H-NMR spectrum of compound **16** (500 MHz, CD_3OD)

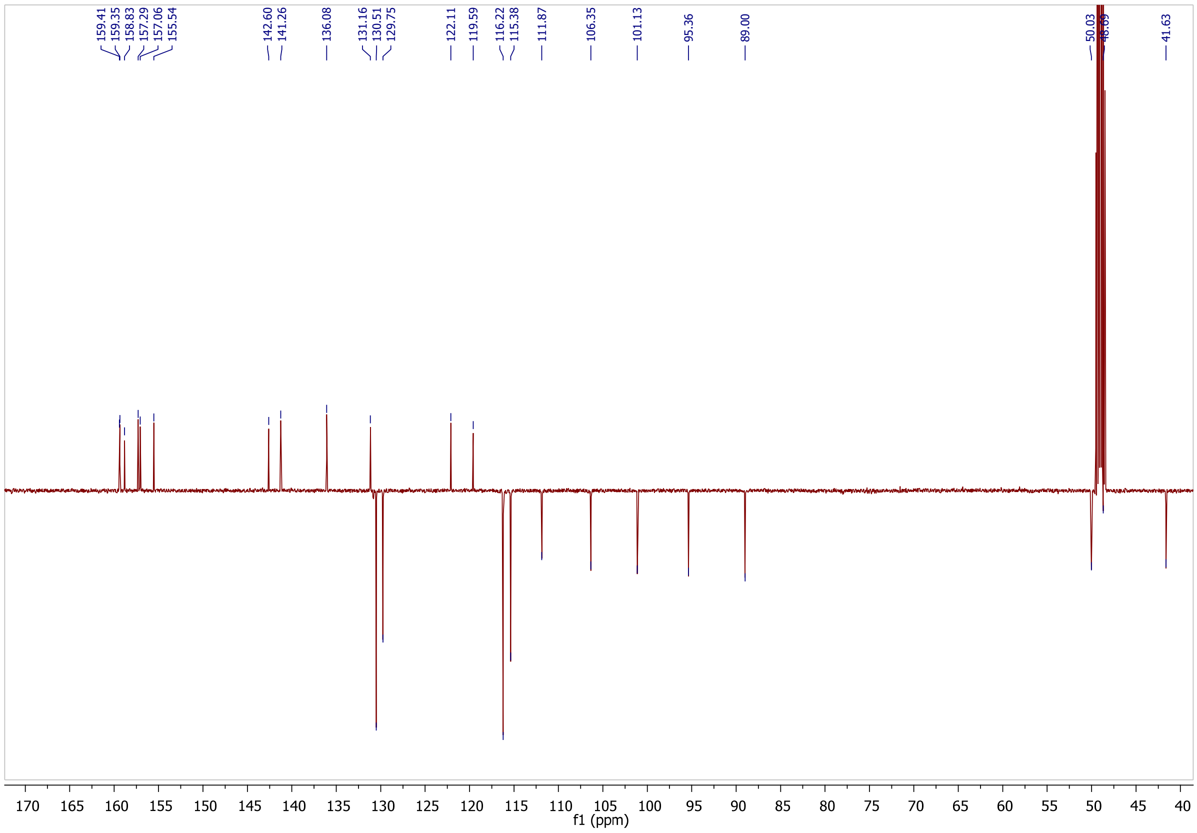

Figure S56. JMOD spectrum of compound **16** (125 MHz, CD_3OD)

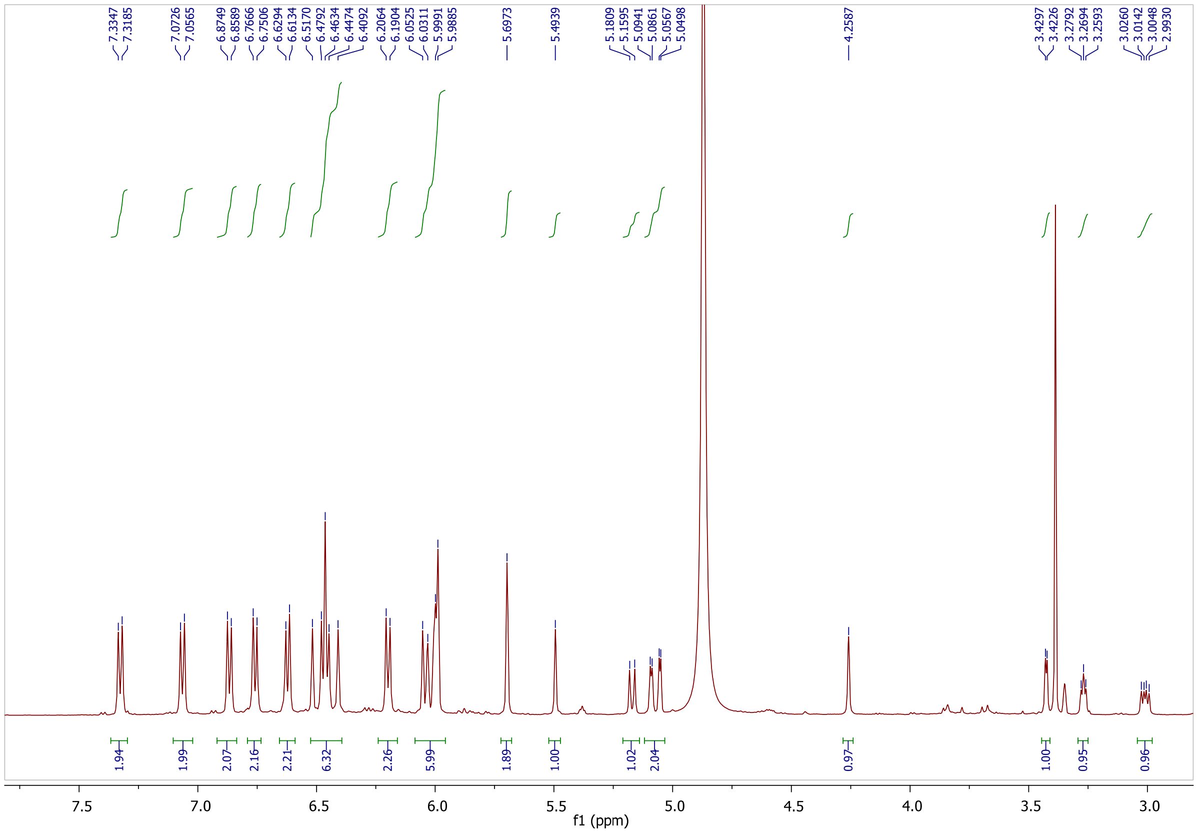

Figure S57. ^{1}H-NMR spectrum of compound **17** (500 MHz, CD_3OD)

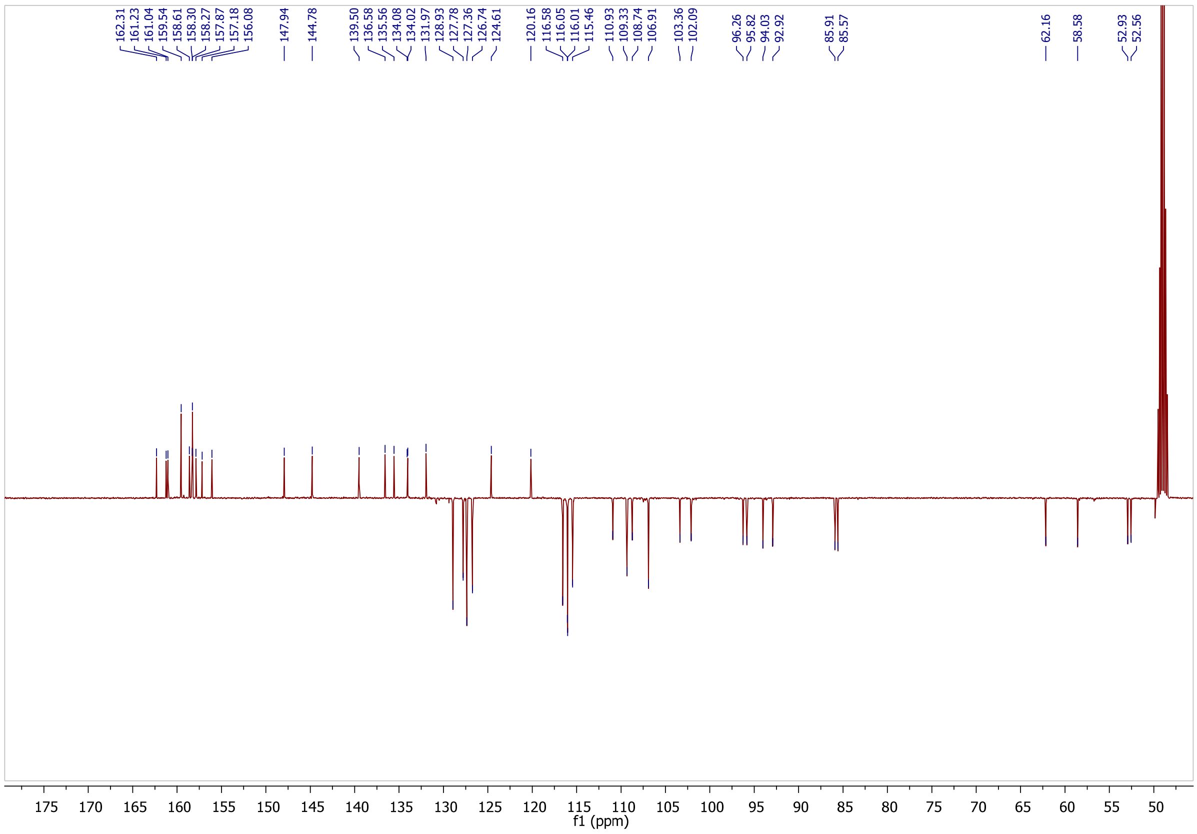

Figure S58. JMOD spectrum of compound **17** (125 MHz, CD_3OD)

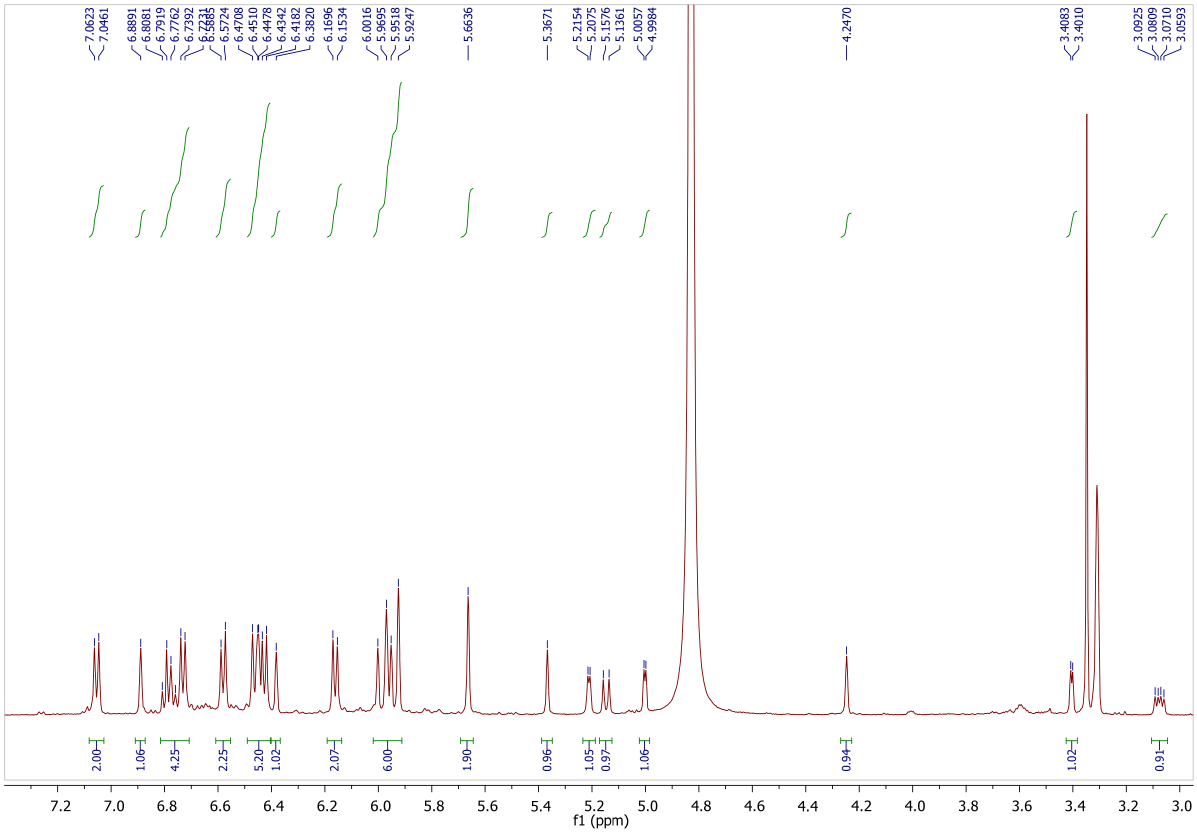

Figure S59. ^{1}H-NMR spectrum of compound **18** (500 MHz, CD_3OD)

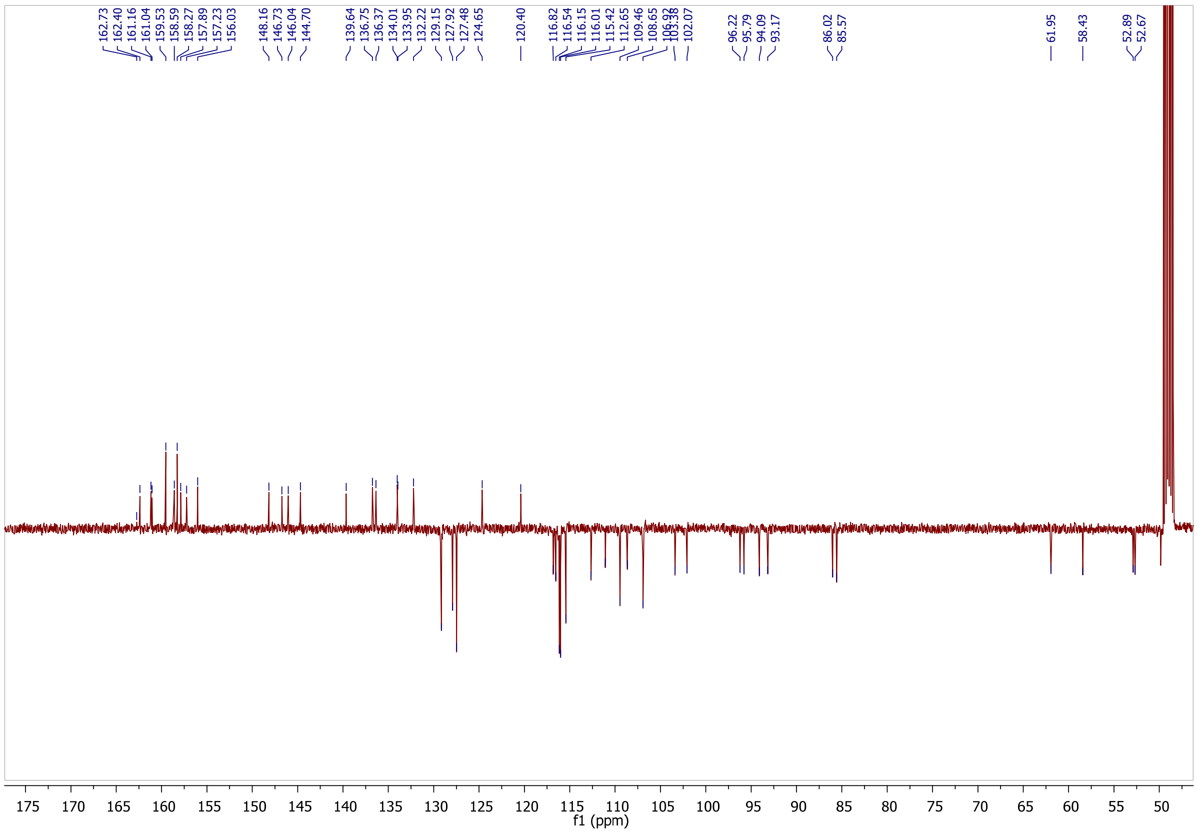

Figure S60. JMOD spectrum of compound **18** (125 MHz, CD_3OD)

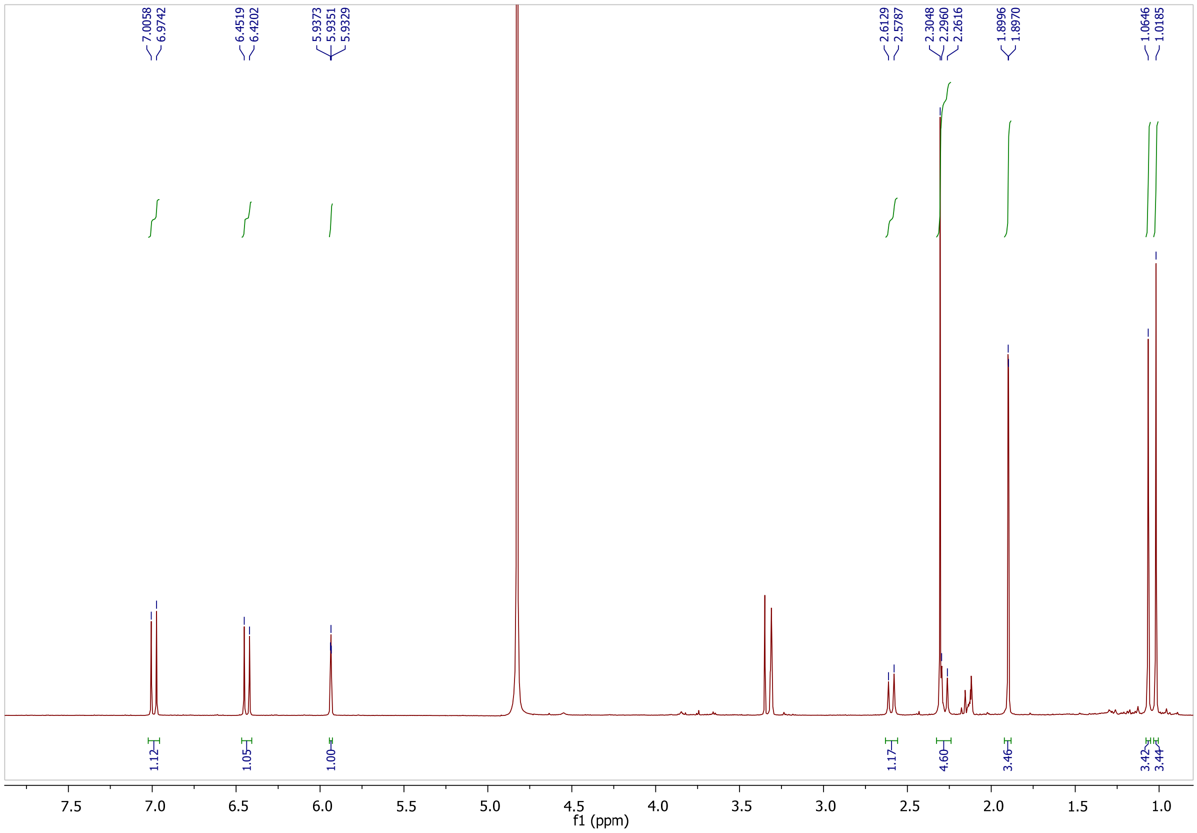

Figure S61. ^{1}H-NMR spectrum of compound **19** (500 MHz, CD_3OD)

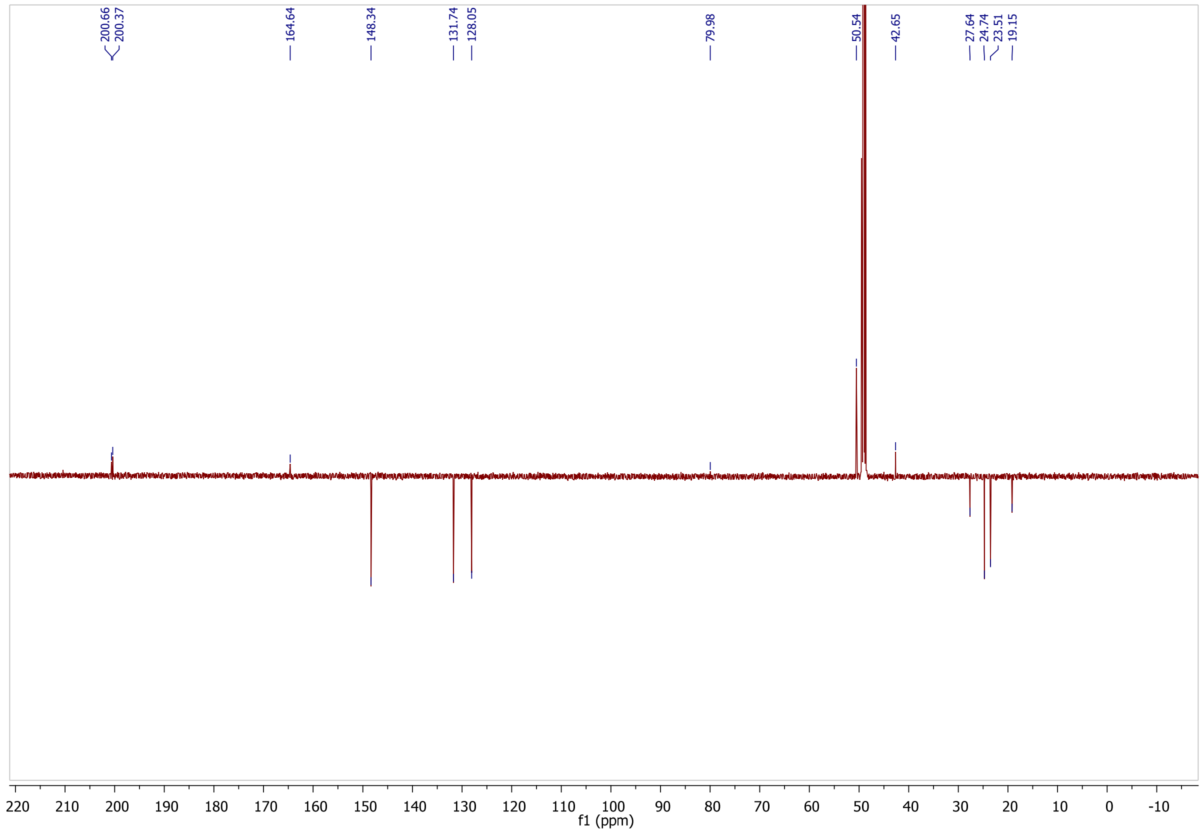

Figure S62. JMOD spectrum of compound **19** (125 MHz, CD_3OD)

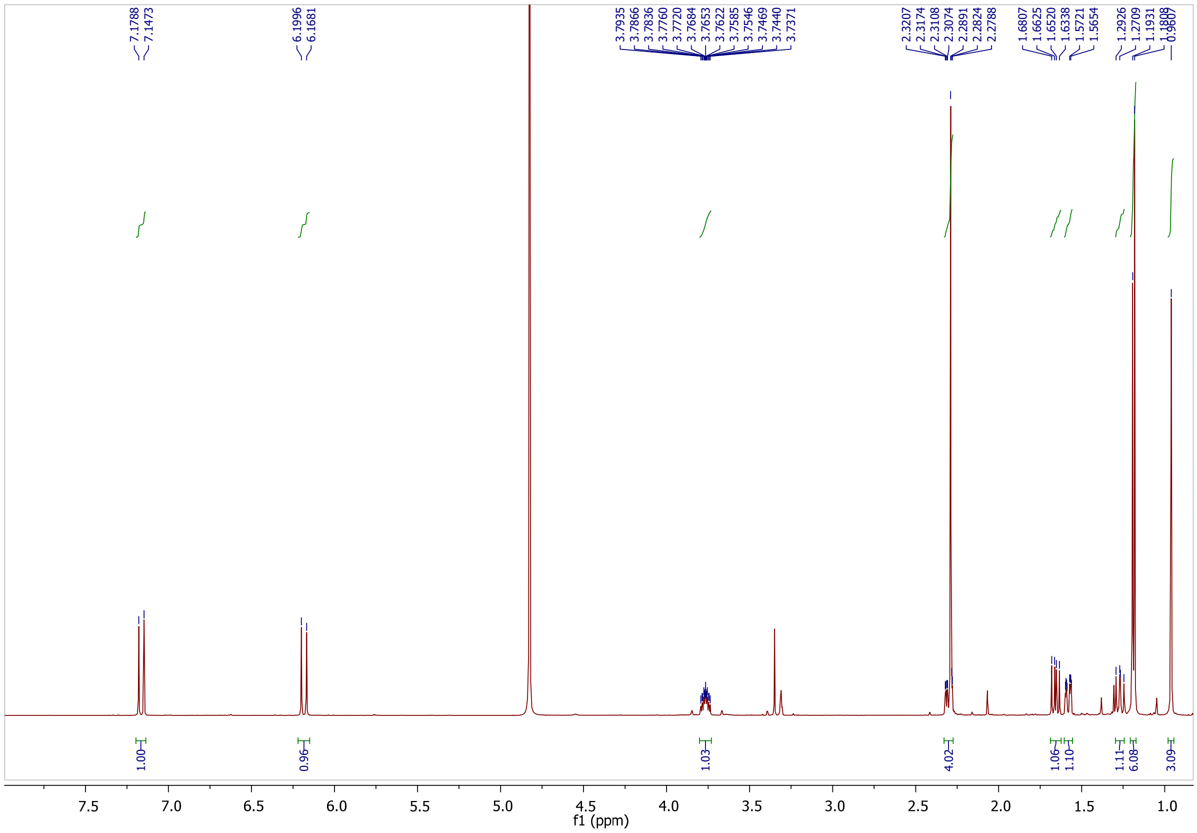

Figure S63. ^{1}H-NMR spectrum of compound **20** (500 MHz, CD_3OD)

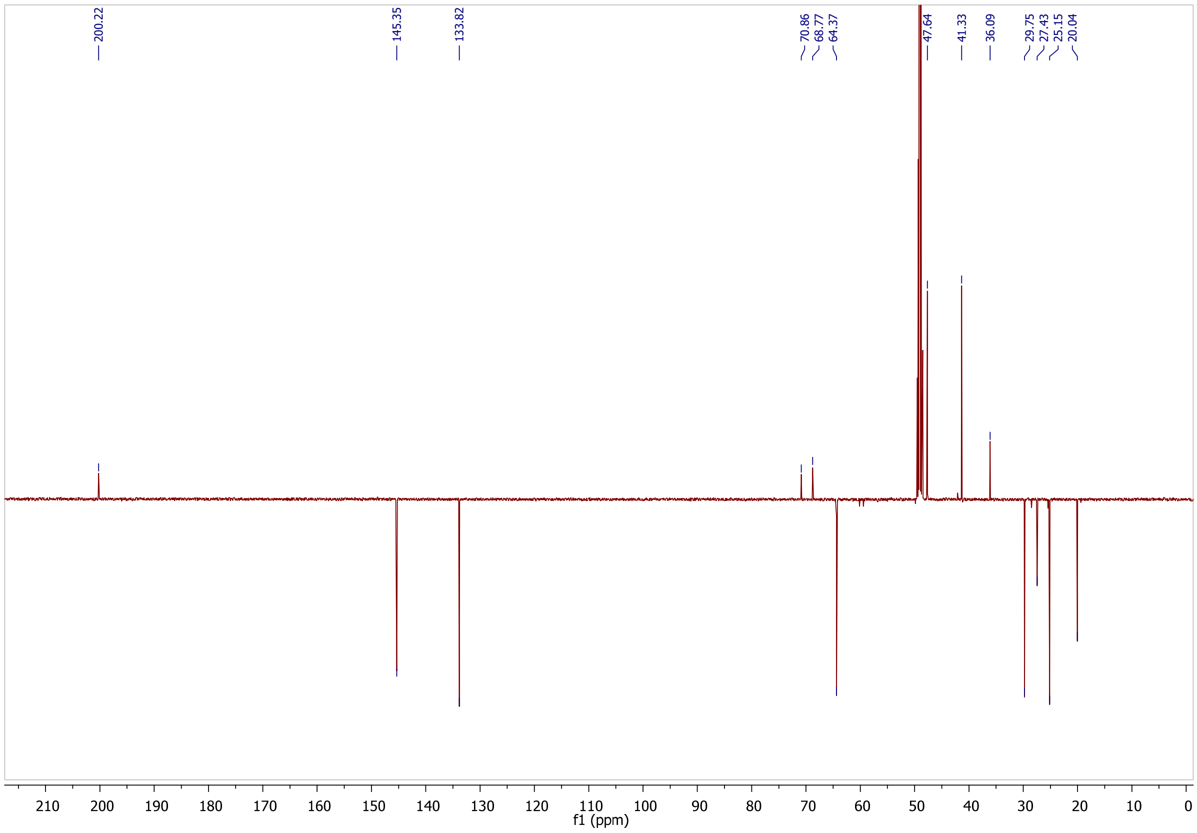

Figure S64. JMOD spectrum of compound **20** (125 MHz, CD_3OD)

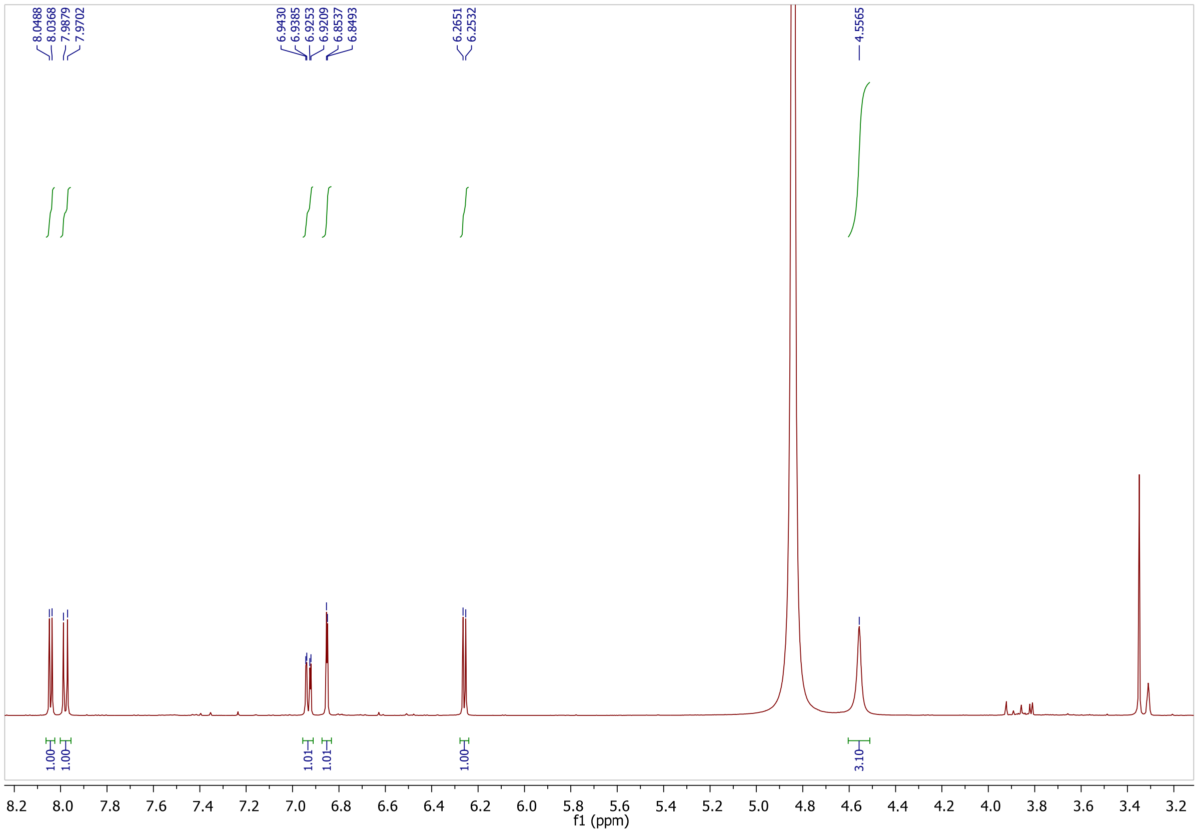

Figure S65. ^{1}H-NMR spectrum of compound **21** (500 MHz, CD_3OD)

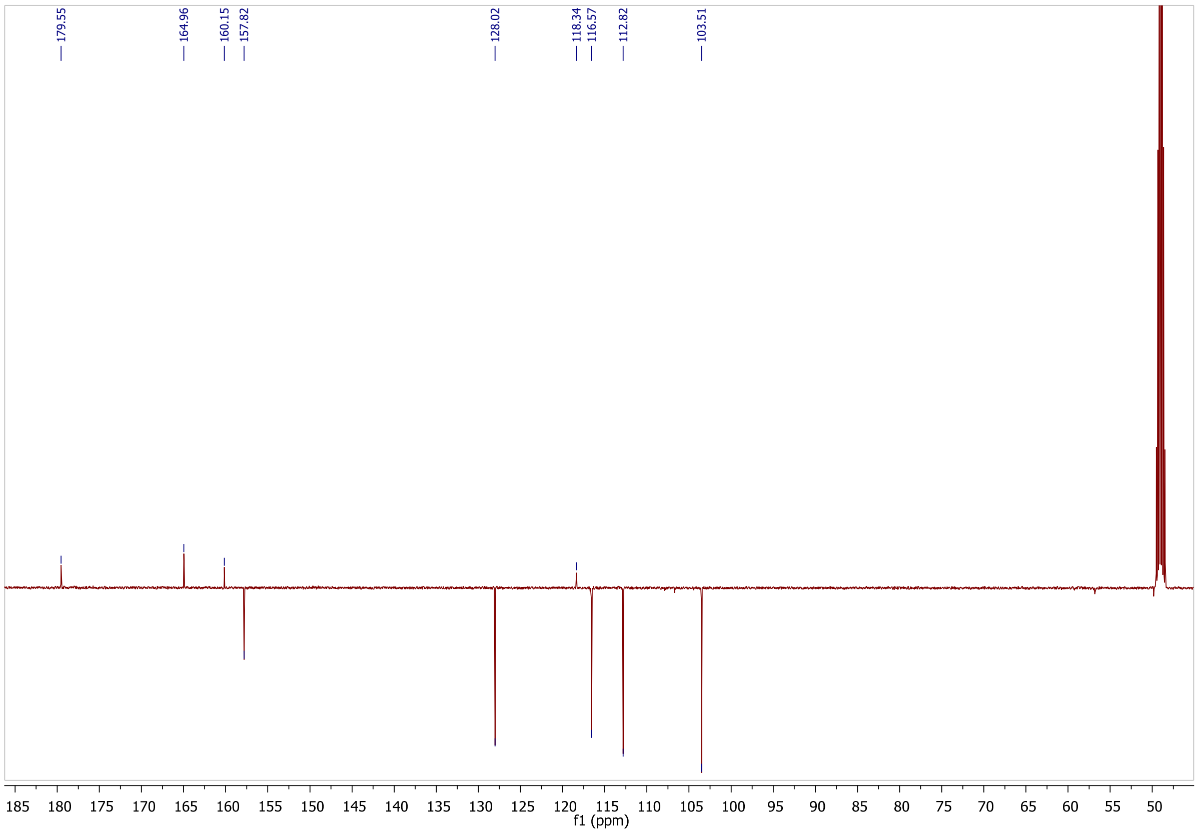

Figure S66. JMOD spectrum of compound **21** (125 MHz, CD_3OD)

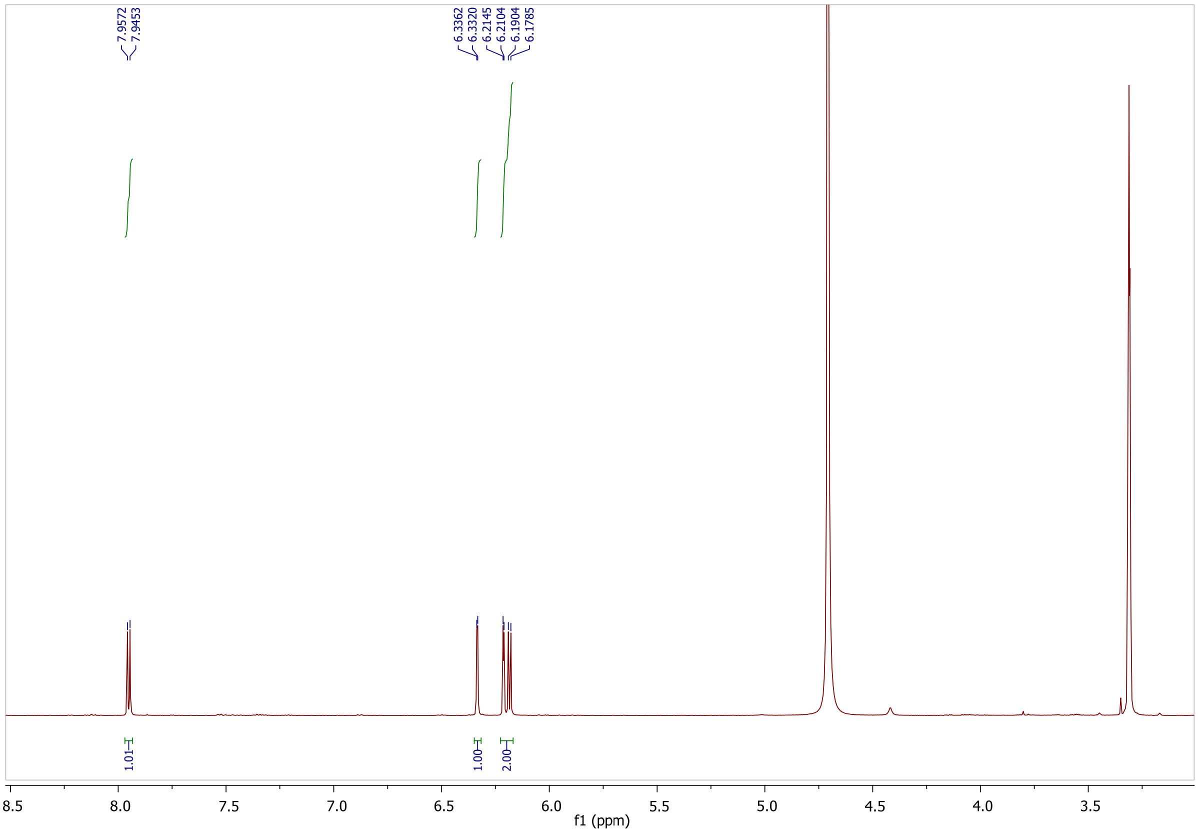

Figure S67. ^{1}H-NMR spectrum of compound **22** (500 MHz, CD_3OD)

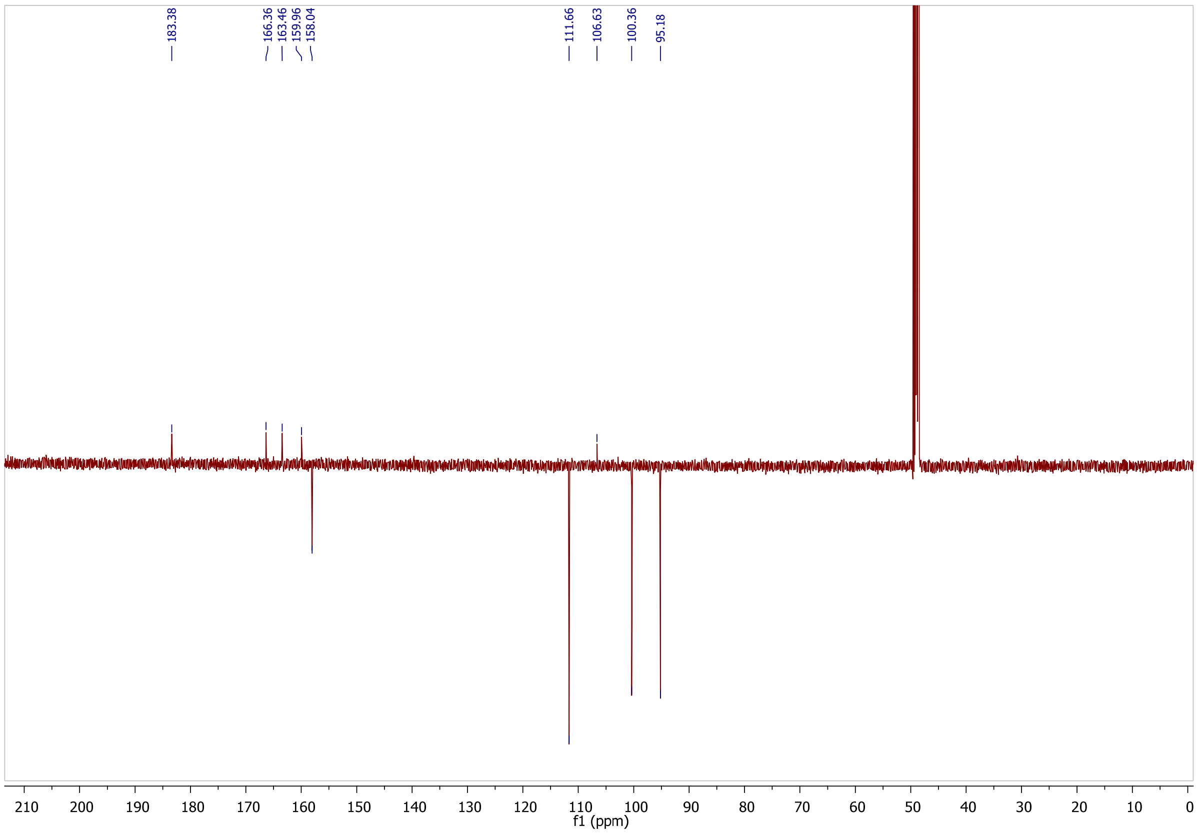

Figure S68. JMOD spectrum of compound **22** (125 MHz, CD_3OD)

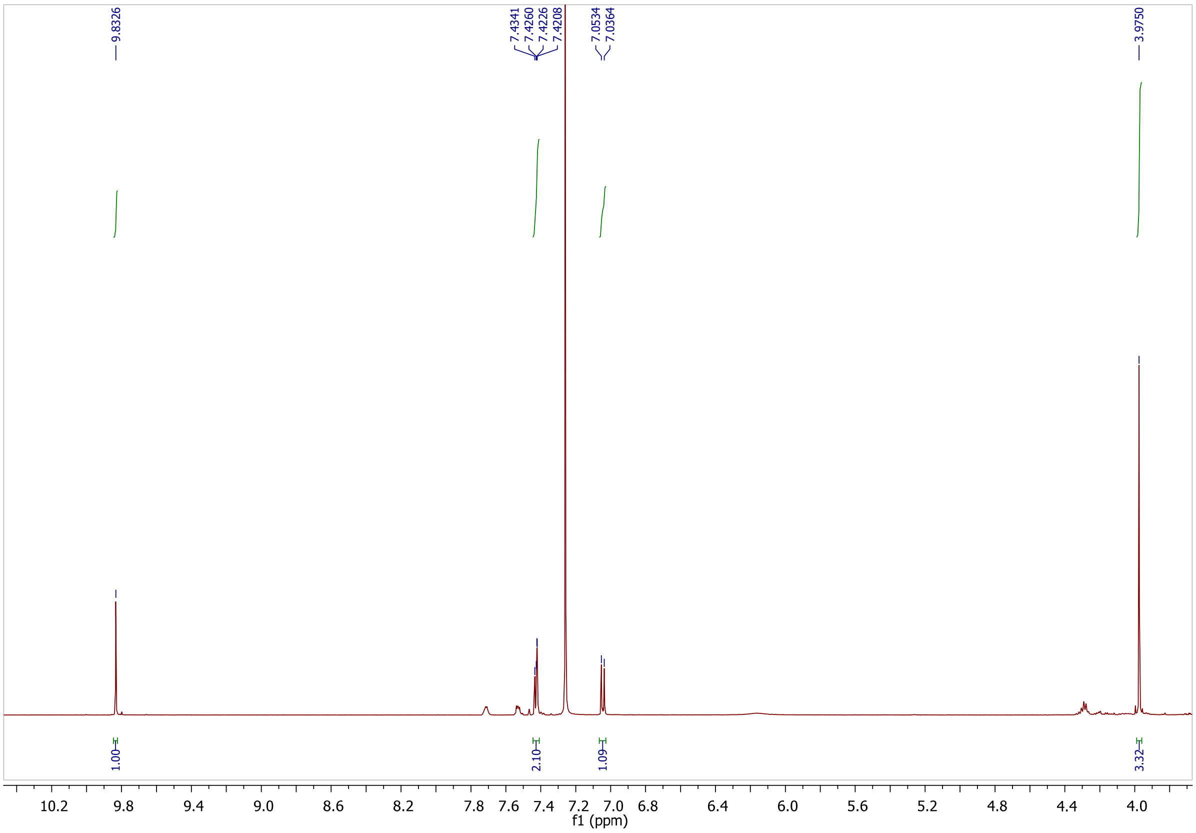

Figure S69. ¹H-NMR spectrum of compound **23** (500 MHz, $CDCl_3$)

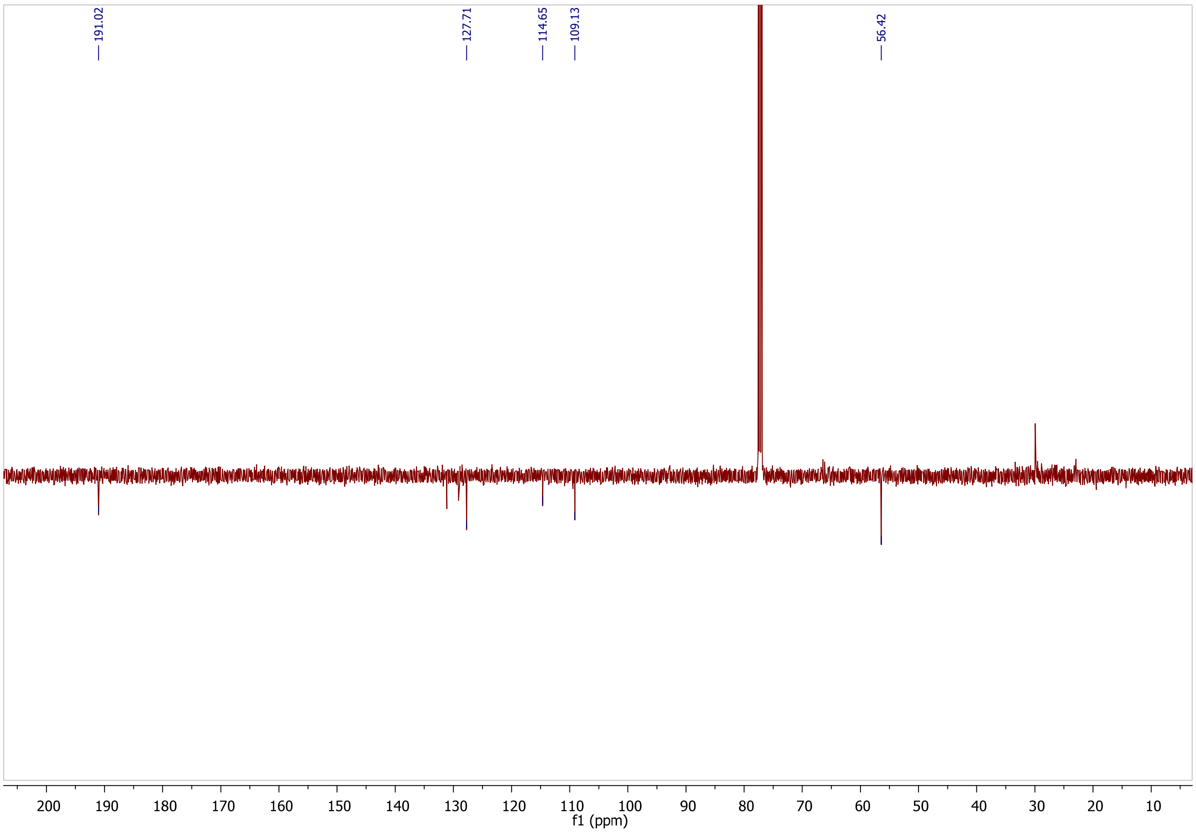

Figure S70. JMOD spectrum of compound **23** (125 MHz, $CDCl_3$)

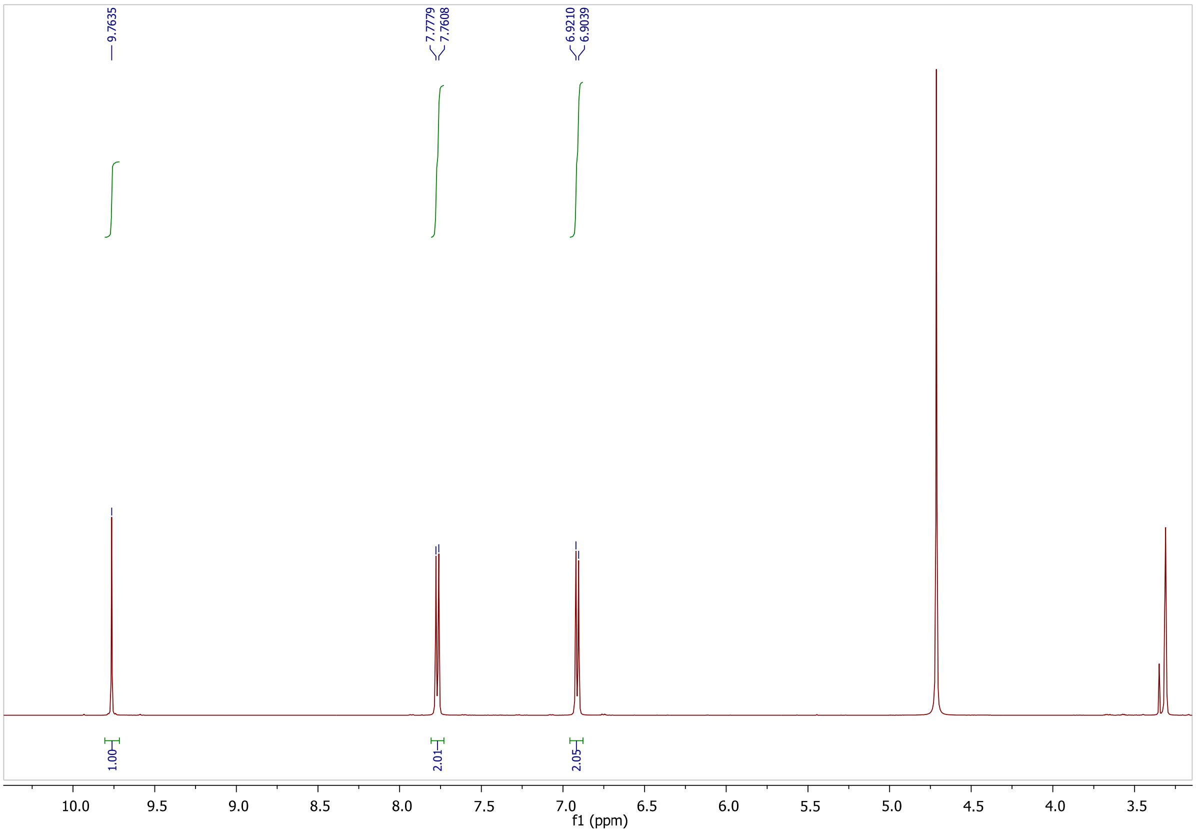

Figure S71. ^{1}H-NMR spectrum of compound **24** (500 MHz, CD_3OD)

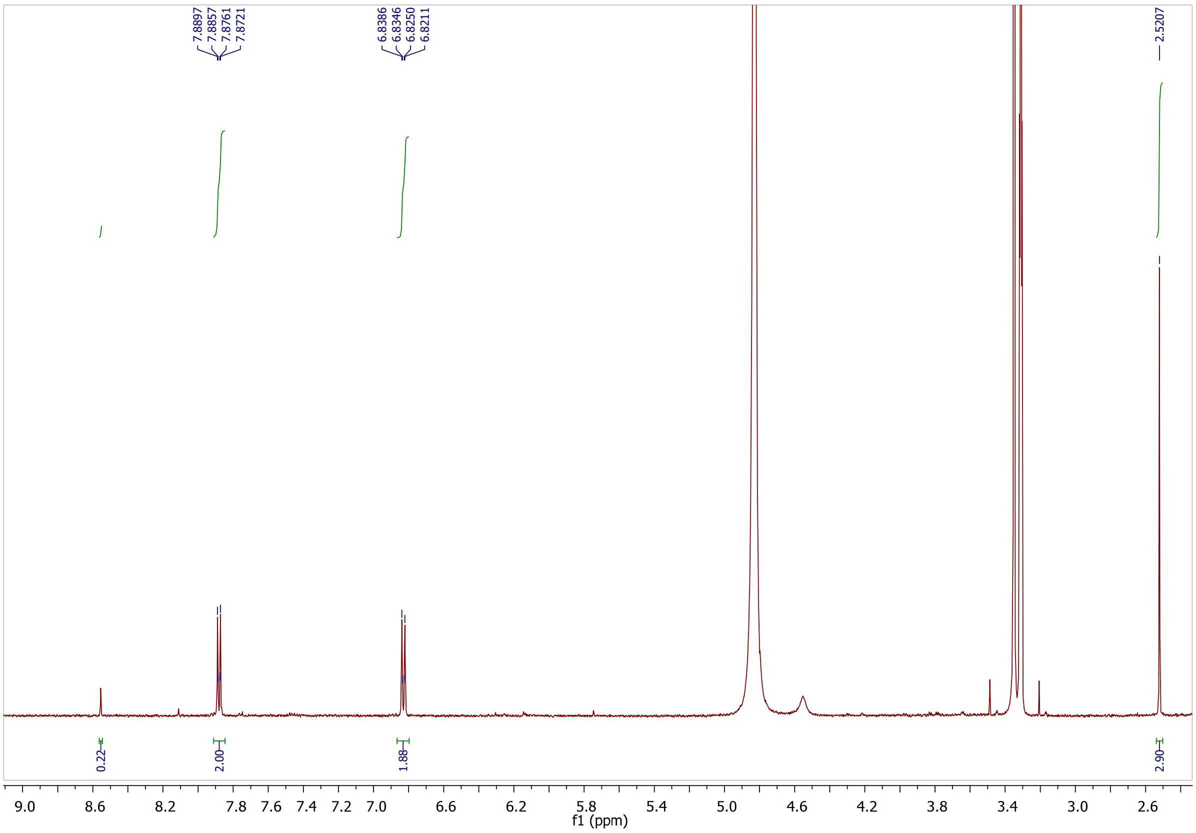

Figure S72. ^{1}H-NMR spectrum of compound **25** (500 MHz, CD_3OD)

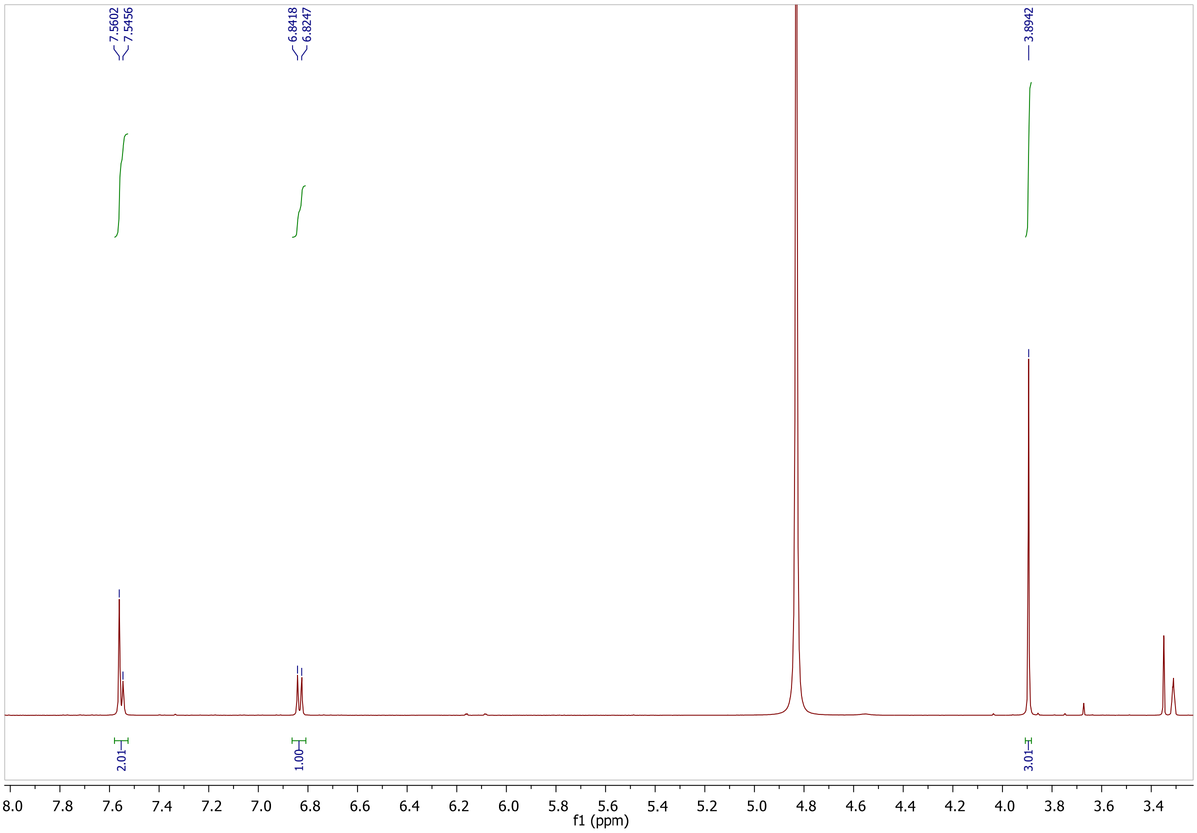

Figure S73. ^{1}H-NMR spectrum of compound **26** (500 MHz, CD_3OD)
